# Sterically Tuned *Ortho*-Phenylene-Linked Donor–Acceptor Benzothiazole-Based Boron Difluoride Complexes as Thermally-Activated Delayed Fluorescence Emitters for Organic Light-Emitting Diodes

**DOI:** 10.1021/acsami.4c12662

**Published:** 2024-10-22

**Authors:** Stepan Kutsiy, Dmytro Volyniuk, Smruti Ranjan Sahoo, Magdalena Ceborska, Agnieszka Wisniewska, Pavlo Stakhira, Juozas Vidas Grazulevicius, Glib V. Baryshnikov, Mykhaylo A. Potopnyk

**Affiliations:** † Institute of Organic Chemistry, Polish Academy of Sciences, Kasprzaka 44/52, 01-224 Warsaw, Poland; ‡ Department of Electronic Devices, Lviv Polytechnic National University, 1 Sviatoho Yura sq., Lviv 79013, Ukraine; § Department of Polymer Chemistry and Technology, Kaunas University of Technology, Barsausko 59, LT-51423 Kaunas, Lithuania; ∥ Laboratory of Organic Electronics, Department of Science and Technology, Linköping University, Norrköping SE-60174, Sweden; ⊥ Department of Physics and Astronomy, Uppsala University Box 516, SE-75120 Uppsala, Sweden; # Faculty of Mathematics and Natural Sciences, Cardinal Stefan Wyszynski University in Warsaw, K. Woycickiego 1/3, 01-938 Warsaw, Poland; ¶ Institute of Physical Chemistry, Polish Academy of Sciences, Kasprzaka 44/52, 01-224 Warsaw, Poland; ∇ Institute of Organic Chemistry, National Academy of Sciences of Ukraine, Akademika Kuharya Str. 5, 02000 Kyiv, Ukraine

**Keywords:** organoboron dye, carbazole, spin−orbit coupling, aggregation-induced emission, solid-state luminescence

## Abstract

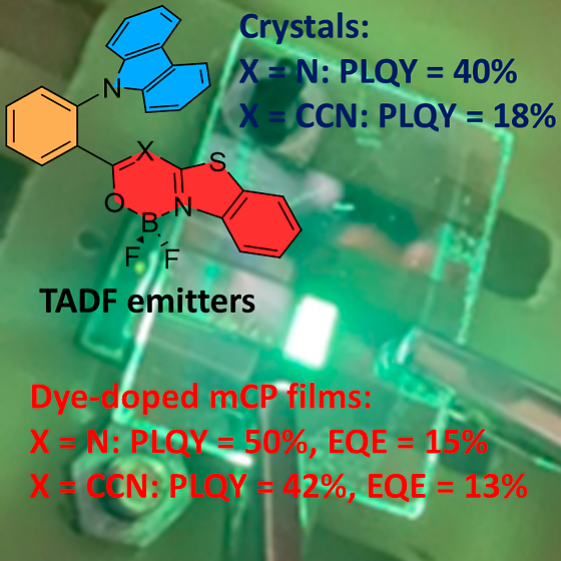

Two donor–acceptor dyes with an *ortho*-phenylene-linked carbazole electron donor and a benzothiazole-fused boron heterocyclic acceptor were designed, synthesized, and spectroscopically investigated. Due to the steric effects of boron heterocyclic units, the dyes demonstrate different conformations in the crystalline state. The presence of numerous hydrogen-bonding intermolecular interactions and the very weak π–π stacking in the molecular packing results in intense solid-state emission with photoluminescence quantum yields of 40 and 18% for crystals and 50 and 42% for host-based light-emitting layers. The compounds show aggregation-induced emission and thermally activated delayed fluorescence (TADF). The received ionization potential and electron affinity values suggested good charge-injecting ability and bipolar charge-transporting properties of the developed dyes. Transport of holes and electrons was detected in layers of one dye by the time-of-flight measurements. The benzothiazole-based boron difluoride complexes showed high electron mobility of 1.5 × 10^–4^ and 0.7 × 10^–4^ cm^2^ V^–1^ s^–1^ at an electric field of 1.35 × 10^6^ V cm^–1^. Therefore, these dyes were successfully applied as emitters in organic light-emitting diodes with external quantum efficiencies of 15 and 13%, respectively. Our study marks a critical advancement in the area of solid-state emissive boron difluoride dyes, which can be applied as TADF emitters into organic light-emitting diodes. The obtained results reveal that the orientation of the acceptor unit in the *ortho*-phenylene-linked donor–acceptor dyes makes a significant impact on the TADF activity.

## 1 Introduction

Boron difluoride complexes belong to a highly attractive fluorescent dye family due to their numerous applications in different areas, including fluorescent probes,^1,2^ bioimaging,^3^ photodynamic therapy,^4,5^ organic lasers,^6,7^ stimuli-responsive materials,^8−10^ and organic photovoltaic devices.^11^ Their applicability is attributed to the synthetic variability and tunable photophysical properties of these dyes.^12−15^ However, notwithstanding the great significance, most of the research works on boron difluoride complexes have been focused on prompt fluorescence emitters. Meanwhile, the investigation of analogues exhibiting delayed fluorescence reported rarely, mostly describing *O*,*O*-chelated boron difluoride complexes.^16−22^

Organic materials exhibiting thermally activated delayed fluorescence (TADF) have been intensively investigated due to their ability to harvest triplet excitons, thereby enhancing electroluminescence efficiency.^23−28^ Such an ability enables the increase of internal and, as a consequence, external quantum efficiencies (EQE’s) of optoelectronic devices based on these emitters. Despite the significant progress made in the past decade, there remains much room for improvement of EQE, which prompts scientists to discover new TADF-active chromophores. Thereby, many TADF-active organic compounds with highly twisted molecular structures were investigated.^29^ Among them, donor–acceptor-type chromophores with *ortho*-configuration via a benzene ring are a particularly interesting group, due to their ability to exhibit two types of intramolecular charge transfer (ICT): through-bond intramolecular charge transfer and through-space intramolecular charge transfer.^30,31^ Thus, *ortho*-phenylene-linked donor–acceptor (D–A)-type dyes usually exhibit TADF^32,33^ or/and room-temperature phosphorescence.^34,35^ The most efficient donor groups in such D–A-type luminescent compounds are carbazole,^32,36,37^ triphenylamine,^38^ and 9,9-dimethyl-9,10-dihydroacridine,^39,40^ while ketones,^38^ sulfones,^39,40^ pyridines,^37^ 1,3,5-triazine,^36^ or triarylborons^32^ have been used as the acceptor units. Meanwhile, as far as we know, boron difluoride complexes have never been used in the design of *ortho*-phenylene-linked D–A dyes.

In this context, recently, we studied *N*,*O*-chelated boron dyes based on benzothiazole-containing ligands.^41,42^ We also demonstrated that such donor–acceptor dyes with *para*-phenylene-linked carbazole donor units exhibit intensive solid-state emission.^43^ The *ortho*-phenylene-linked D–A molecules demonstrated bulky steric hindrance in comparison to their *para*-phenylene-linked analogues. Therefore, the modification of these dye structures via changing the carbazole donor position looks attractive in terms of successful TADF emitter discovery.

**Figure 1:**
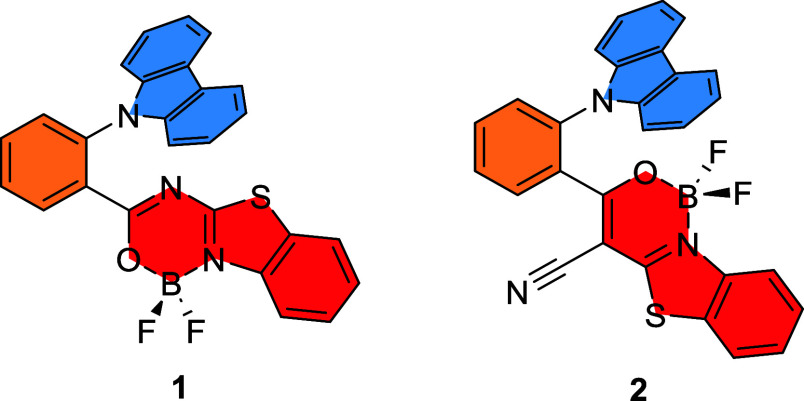
Boron difluoride complexes **1** and **2**.

Herein, we describe the facile low-cost synthesis of two *ortho*-phenylene-linked donor–acceptor boron dyes **1** and **2** based on a carbazole donor and benzothiazole-fuzed oxadiazaborinine and oxazaborinine acceptors (Figure 1). Compound **2** includes a cyano group, which increases the acceptor strength of the benzo[4,5]thiazolo[3,2-*c*][1,3,2]oxazaborinine moiety and also impacts the steric effect to twist this acceptor unit comparatively to the *ortho*-linked phenylene. The synthesized dyes have been thoroughly characterized, including single-crystal analysis and thermal and electrochemical measurements. Then, comprehensive studies, which combine theoretical calculations and photophysical investigation, both in solution and the solid state, were performed. Finally, we studied the applications of dyes **1** and **2** in organic light-emitting diodes (OLEDs).

## 2 Results and Discussion

### 2.1 Synthesis and Characterization

**Scheme 1:**
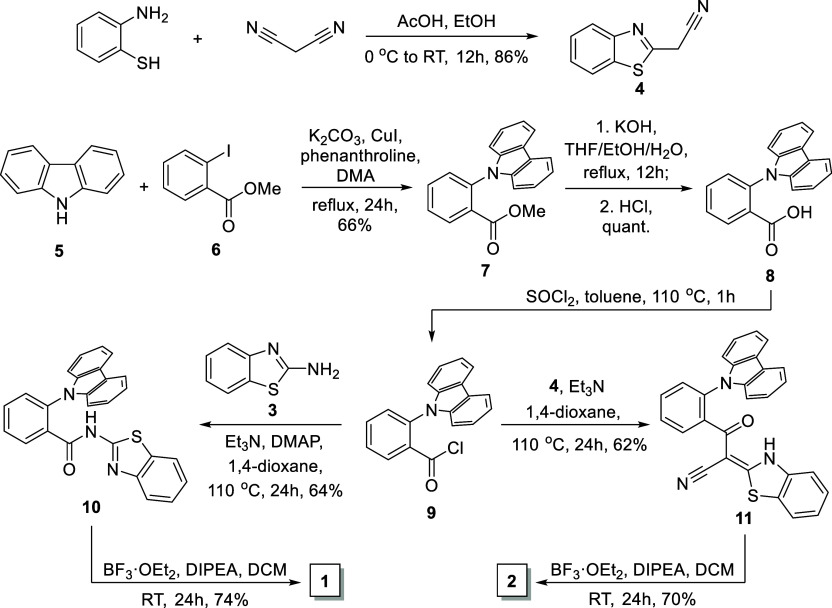
Synthesis of *ortho*-Phenylene-Linked Donor–Acceptor Boron Difluoride Complexes **1** and **2**

The synthesis of compounds **1** and **2** is presented in Scheme 1. Benzo[*d*]thiazol-2-amine (**3**) and 2-(benzo[*d*]thiazol-2-yl)acetonitrile (**4**) were selected as the starting benzothiazole building blocks. Amine **3** was commercially available, while cyano derivative **4** was obtained by the reaction of 2-aminothiophenol with malononitrile in high yield (86%).

The Ullmann amination reaction of carbazole (**5**) with methyl 2-iodobenzoate (**6**) results in methyl 2-(9*H*-carbazol-9-yl)benzoate (**7**) in 66% yield, which was hydrolyzed, giving 2-(9*H*-carbazol-9-yl)benzoic acid (**8**) in a quantitative yield. Acid **6** was treated with thionyl chloride in toluene, giving the corresponding *ortho*-substituted benzoyl chloride **9**. Compound **9** was used without purification in the acylation reaction with amine **3** and nitrile **4** in basic conditions, giving products **10** and **11** in 64 and 62% yield, respectively. Compounds **10** and **11** were used as ligands in the reaction with boron trifluoride diethyl etherate in the presence of diisopropylethylamine (DIPEA) to let the final boron difluoride complexes **1** and **2** be obtained in 74 and 70% yield, respectively. All synthesized compounds were thoroughly characterized by nuclear magnetic resonance (^1^H, ^13^C, and ^19^F NMR) spectroscopy, as well as by high-resolution mass spectrometry (HRMS).

**Figure 2:**
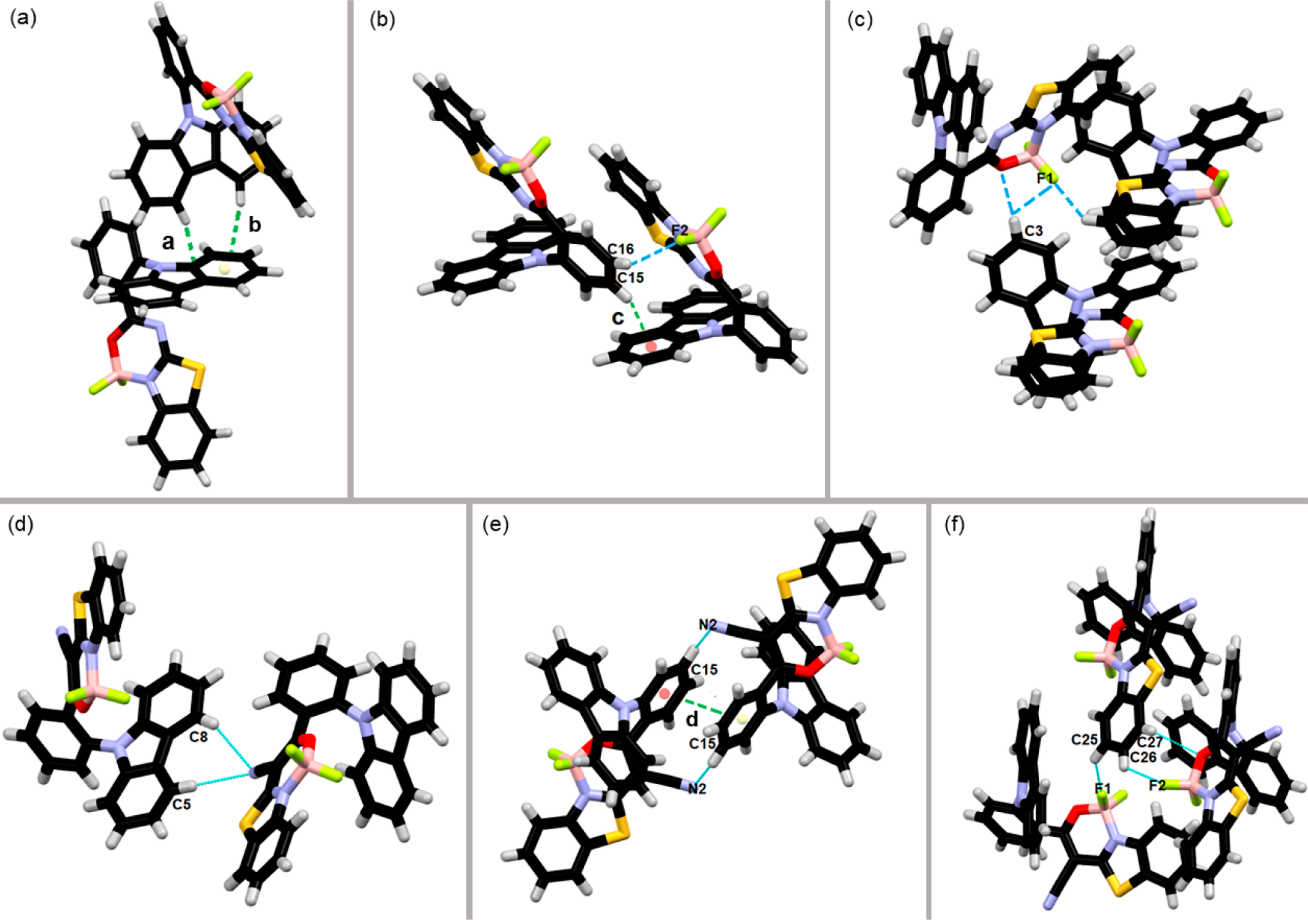
Crystal packing of compounds **1** (a–c) and **2** (d–f): (a) CH···π interaction stabilizing the dimer of dye **1**; (b) CH···π and CH···F interactions of dye **1**; (c) CH···F and CH···O interactions; (d) two symmetrical weak intermolecular CH···N-type interactions of dye **2**; (e) π–π and CH···π interactions between symmetrically related molecules of compound **2**; (f) CH···F and CH···O interactions of dye **2**.

In order to determine the structural geometry and molecular packing of boron difluoride complexes **1** and **2** in the crystalline state, a single-crystal X-ray diffraction analysis was performed (Figures S1 and S2, Table S1 in the Supporting Information). Oxadiazaborinine derivative **1** crystallizes in the tetragonal space group *P*-42_1_*c*. The whole molecule is bent, which is characterized by the torsion angles equal to 99.7° (C1–N1–C13–C18) and −17.03° (C13–C18–C19–N2). This conformation is stabilized by weak N···π interaction (2.926 Å, Figure S3 in the Supporting Information). Two molecules of compound **1** form dimers via CH···π interactions (***a*** = 3.875 Å, ***b*** = 3.849 Å, Figure 2a). The whole structure is further stabilized by another CH···π interaction (***c*** = 3.741 Å, Figure 2b), as well as CH···F (C3H3···F1 = 3.332 Å, C16H16···F2 = 3.403 Å, C26H26···F1 = 3.307) and CH···O interactions (C3H3···O1 = 3.658) (Figure 2b,c). A detailed analysis of weak intermolecular interactions is presented in Table S2.

Oxazaborinine derivative **2** crystallizes in the monoclinic space group *P*2_1_/*c*. The molecule adapts bent conformation described by torsion angles, which are equal to −11.63° (C1–N1–C13–C18) and −17.03° (C13–C18–C19–N2). The N2 nitrogen atom from the cyano group forms two symmetrical weak CH···N-type interactions with CH groups from two phenyl rings of the carbazole moiety of a symmetrically related molecule **2** (C3H3···N2 = 3.500 Å and C8H8···N2 = 3.511 Å, Figure 2d). Another symmetrically related molecule is held by π–π interactions between two phenyl rings (C13–C18 and C13′-C18′, π–π = 3.941 Å), Figure 2e. The crystal structure of complex **2** is further stabilized by CH···F (C25H25···F1 = 3.292 Å and C26H26···F2 = 3.170 Å) and CH···O (C27H27···O1 = 3.317 Å) interactions, Figure 2f. A detailed analysis of weak intermolecular interactions is presented in Table S3.

The structurally related compounds **1** and **2** adapt a similar “bent” conformation (Figure S4 in the Supporting Information), although the incorporation of a nitrile group into the **2** moiety induces some changes in the molecule conformation, which translates to different types of weak interactions occurring in the structure. The torsion angles (C13–C18–C19–N2) are the same for both compounds **1** and **2**, while the torsion angles (C1–N1–C13–C18) are different (99.7° for structure **1** and −11.63° for analogue **2),** which render reverse positioning of the BF_2_-containing moiety, as presented in the structural overlay in Figure S5 in the Supporting Information.

### 2.2 Thermal, Electrochemical, and Charge-Transporting Properties

To evaluate the thermal properties of difluoroboron dyes **1** and **2**, thermogravimetric analysis (TGA) and differential scanning calorimetry (DSC) measurements were performed. TGA curves (Figure S6 in the Supporting Information) show that the decomposition temperature (*T*_d_, corresponding to a 5% weight loss) of compounds **1** and **2** is approximately of 342 and 357 °C, respectively. This indicates the robust thermal stability of the investigated dyes. DSC experiments combined heating from −40 to +280 °C, cooling from +280 to −40 °C, and second heating from −40 to +280 °C (Figure S7 in the Supporting Information). During the first heating, compounds **1** and **2** demonstrated one strong peak at 170 and 263 °C, respectively, which correspond to the melting point temperature. During cooling, the materials show no phase transitions. However, during the second heating, there was no peak characteristic of melting appearing; only a drop suggesting a glass transition was observed at 96 and 116 °C for compounds **1** and **2**, respectively. This indicates that boron complex **2** should have better morphological stability in the solid state, compared to dye **1**.

The electrochemical properties of boron difluoride complexes **1** and **2** were measured by cyclic voltammetry (CV) (Figure S8 and Table S4 in Supporting Information). The values of ionization potentials (IPs) and electron affinities (EAs) were calculated using the equilibriums: IP = *E*_ox_ + 4.8 and EA = *E*_red_ + 4.8, where *E*_ox_ and *E*_red_ are the oxidation and reduction potentials, respectively. Boron difluoride dye **1** demonstrates slightly lower values of both IP and EA (5.70 and 3.06 eV, respectively), compared to the cyano analogue **2** (IP = 5.75 eV, EA = 3.13 eV).

Additionally, we measured the electron photoemission spectra of neat films of compounds **1** and **2** by ultraviolet photoelectron spectroscopy (UPS) in air (Figure S9 in the Supporting Information). The obtained values of IPs (5.76 eV for boron complex **1** and 5.81 eV for dye **2**) are comparable to those received from the CV measurements.

To examine the charge-transporting properties of boron difluoride complexes **1** and **2**, we fabricated samples with the structure of an indium tin oxide (ITO)/thin vacuum-deposited layer of dye/Al and used the time-of-flight (time of flight (TOF)) method. Compound **1** demonstrates an electron-transporting ability. In contrast, cyano derivative **2** shows both electron- and hole-transporting properties (Figure S10 in Supporting Information). The electron mobility values of 1.5 × 10^–4^ and 0.7 × 10^–4^ cm^2^ V^–1^ s^–1^ at an electric field of 1.35 × 10^6^ V cm^–1^ were obtained for the vacuum-deposited layers of dyes **1** and **2** by TOF measurements, respectively (Table S5 in the Supporting Information). These electron mobility values are comparable with the hole mobility values of the widely used functional materials of OLEDs, e.g., *N*,*N*′-dicarbazolyl-3,5-benzene (mCP) (4 × 10^–4^ cm^2^ V^–1^ s^–1^ at an electric field of ca. 4.2 × 10^5^ V·cm^–1^).^44^ In addition, a hole mobility value of 1 × 10^–6^ cm^2^ V^–1^ s^–1^ was detected for dye **2** at the same electric field. OLED hosts typically have higher hole mobility values than their electron mobility values.^45^ Keeping this in mind, our invention of TADF emitters with high electron mobility values have the potential to improve the hole–electron balance in the light-emitting layers of OLEDs based on the conventional hosts.

### 2.3 Photophysical Properties of the Solutions

**Figure 3:**
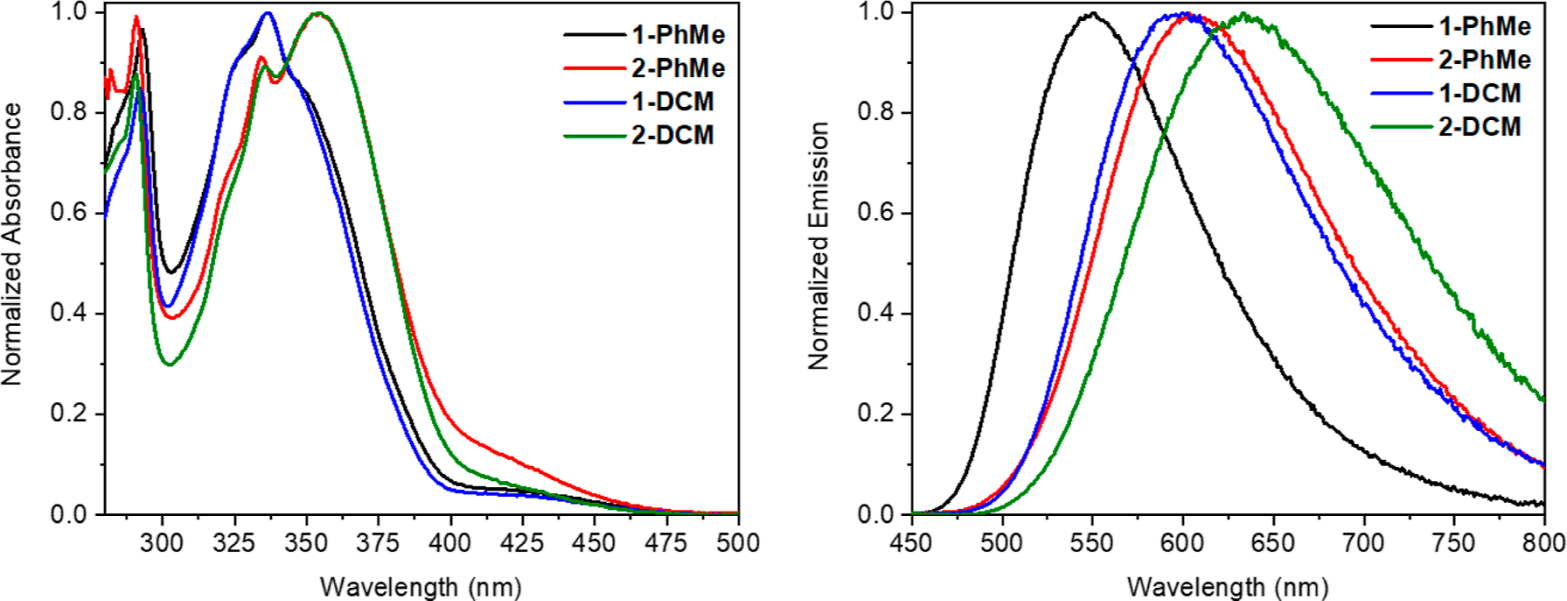
Absorption (left) and photoluminescence (right) spectra of dilute solutions of boron difluorides **1** and **2**.

First, absorption and photoluminescence properties of compounds **1** and **2** were measured in diluted toluene (PhMe) solutions (at the concentration of ∼10^–5^ M). The compounds demonstrate the lowest-energy (S_0_→ S_1_ transition) absorption bands peak at 337 nm (ε = 2.25 × 10^4^ M^–1^ cm^–1^) and 355 nm (ε = 1.77 × 10^4^ M^–1^ cm^–1^) for dyes **1** and **2**, respectively (Figure 3). Dye **1** exhibits a broad emission peak maximized at 551 nm and a photoluminescence quantum yield of 0.13, while the CN analogue **2** demonstrates a bathochromically shifted emission peak (λ_em_ = 600 nm) with decreased PLQY (∼0.01). Both compounds demonstrate biexponential fluorescence decay with the first component of 13.3 and 6.5 ns and the second component of 345.5 and 127.6 ns for dyes **1** and **2**, respectively (Table 1, Figure S13 in the Supporting Information). This suggests that in diluted toluene solutions, the investigated boron dyes exhibit both prompt and delayed fluorescence. The latest originated from triplet states. The intersystem crossing (ISC) and reverse intersystem crossing (RISC) processes of the dyes are very efficient since their emissions are very sensitive to the presence of oxygen. Thus, the high ratio of 2:1 was obtained between PL intensities of deoxygenated and nondeoxygenated toluene solutions for dye **1**, while in the case of corresponding solutions of dye **2,** this ratio was of 1.13:1 (Figure S14 in the Supporting Information).

**Table 1:** Photophysical Properties of the Solutions of Boron Difluoride Complexes 1 and 2

dye	solvent	λ_abs_, nm^a^	ε, M^–1^ cm^–1^^b^	λ_em_, nm^c^	Δυ, cm^–1^^d^	PLQY	τ_1_, ns^e^	τ_2_, ns^e^
**1**	PhMe	337	22 500	551	11 524	0.13	13.3	345.5
	DCM	337	26 300	603	13 007	0.02		
**2**	PhMe	355	17 700	600	11 585	0.01	6.5	127.6
	DCM	355	23 300	633	12 371	<0.01		

a Wavelength of absorption maximum.

b Molar absorption coefficient.

c Wavelength of emission maximum.

d Stokes shift.

e Excited-state lifetime.

To study the influence of the environment polarity on the dye photophysical properties, we also performed measurements for dilute dichloromethane (DCM) solutions. The absorption spectra are very similar to those in toluene with the same absorption maxima at 337 nm (ε = 2.63 × 10^4^ M^–1^ cm^–1^) for dye **1** and 355 nm (ε = 2.33 × 10^4^ M^–1^ cm^–1^) for compound **2** (Figure 3, Table 1). In contrast, the emission spectra demonstrate a bathochromic shift: λ_em_ = 603 and 633 nm for dyes **1** and **2**, respectively, accompanied by decreasing photoluminescence quantum yield (0.02 and <0.01 for boron complexes **1** and **2**, respectively). This observation confirms the ICT process in the excited state of these dyes.

To obtain the information about the energy levels of the lowest excited singlet and triplet states (S_1_ and T_1_), we measured the photoluminescence and phosphorescence (with 0.1 ms delay) spectra of the dyes in dilute tetrahydrofuran (THF) solutions at a low temperature (77 K) (Figure S15 in Supporting Information). The energies of S_1_/T_1_ states are determined as 2.67/2.66 and 2.74/2.56 eV for boron difluoride complexes **1** and **2**, respectively. As a consequence, both dyes have a very small energy gap between the S_1_ and T_1_ levels (Δ*E*_ST_ = 0.01 eV for compound **1** and 0.18 eV for analogue **2**), which clearly indicates their TADF ability. This claim is in very good agreement with spectroscopic measurements at different temperatures (Figures S16 and S17 in the Supporting Information). The increasing emission intensity with increasing temperature is seen until nonradiative relaxation processes are dominated.

### 2.4 Aggregation-Induced Emission

**Figure 4:**
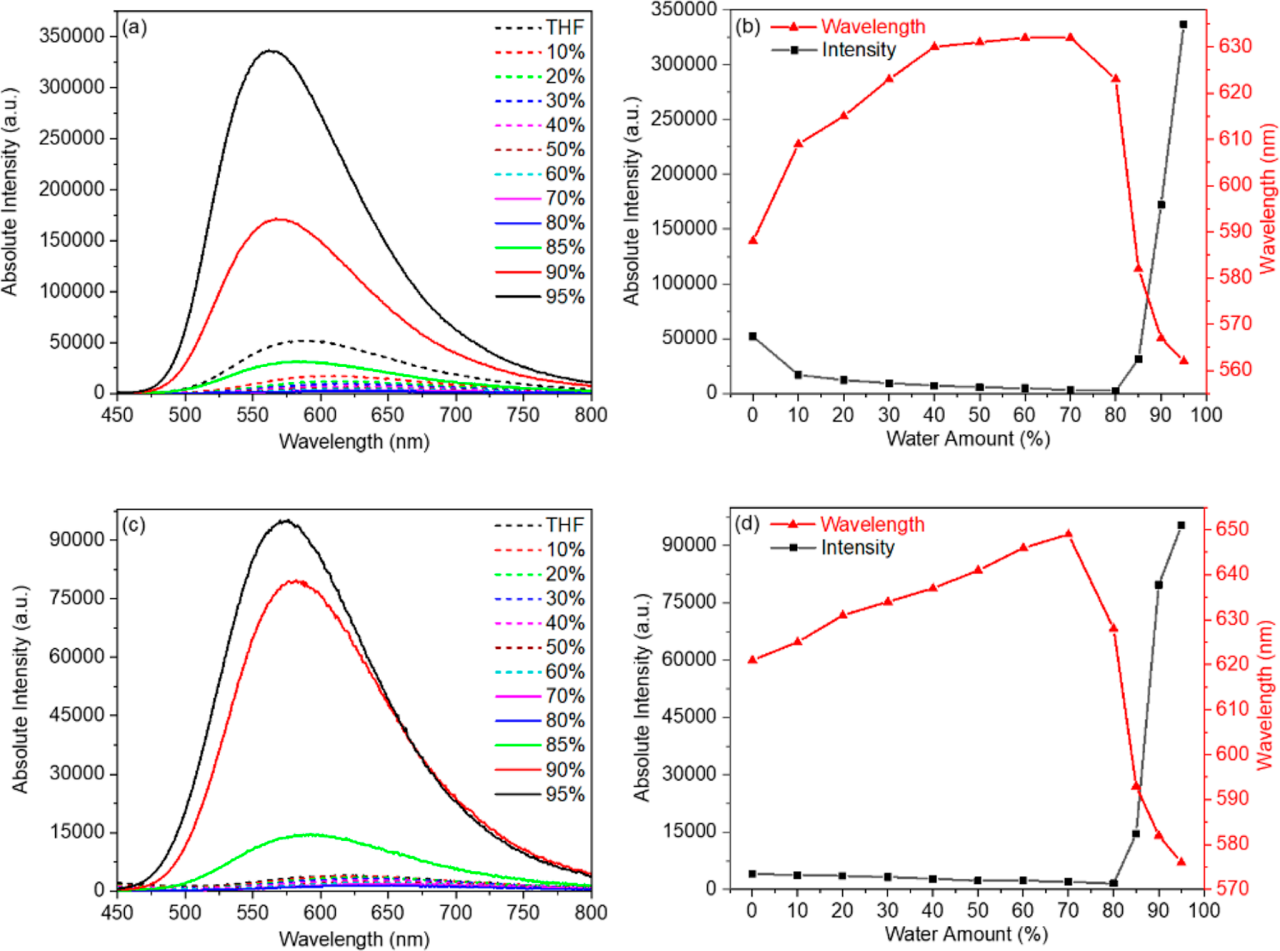
Photoluminescence spectra of the dispersions of dyes **1** (a) and **2** (c) in THF/water mixtures of varying water contents. The dye concentration used was 2.5 × 10^–6^ M, λ_ex_ = 390 nm. Plots of emission intensity (black) and emission maximum (red) of boron difluoride complexes **1** (b) and **2** (d) versus *f*_*w*_.

The observed low photoluminescence efficiency of compounds **1** and **2** in solution prompted us to investigate their aggregation-induced emission (AIE) ability. Therefore, the absorption (Figure S18 in the Supporting Information) and photoluminescence spectra (Figure 4a,c) of both dyes (*C* = 2.5 × 10^–6^ M) in THF/water mixtures with different water volume fractions (*f*_w_) were measured. In all investigated samples, both boron difluoride complexes demonstrate almost no change in the absorption spectra, with the absorption maxima of 334–337 nm for dye **1** and 352–356 nm for dye **2** (Figure S18 in the Supporting Information). This indicates that the surrounding medium does not impact the absorption properties of the investigated dyes.

The emission intensities of both dyes are weak in THF solution and decrease with the increase of the *f*_w_ value in THF/water mixtures up to *f*_w_ = 80%. Due to the increase of the solvent polarity, the emission maxima are shifted from 588 nm in THF (*f*_w_ = 0%) to 632 nm in THF/water = 3:7 (*f*_w_ = 70%) for dye **1** and from 621 nm in THF (*f*_w_ = 0%) to 649 nm in THF/water = 3:7 (*f*_w_ = 70%) for dye **2** (Figure 4b,d). The solutions with *f*_w_ = 80% are characterized by a negligible emission intensity, while the emission maxima are slightly hypsochromically shifted (λ_em_ = 623 and 628 nm for compounds **1** and **2**, respectively). In contrast, the samples with the water amount higher than 80% demonstrate an abrupt increase in the emission intensity accompanied by a significant hypsochromic shift in the emission spectra. Thus, for *f*_w_ = 95%, the emission maxima are at 562 and 576 nm for dyes **1** and **2**, respectively, and are close to the corresponding values for crystalline samples (see next section, Table 2). This observation clearly confirms the AIE behavior of both dyes.

**Table 2:** Photoluminescence Data of Complexes **1** and **2** in the Crystalline State and of Thin Films of the Dyes Doped in mCP (20% of Dye)

dye	sample	λ_em_, nm^a^	PLQY	τ_PF_, ns^b^	τ_DF_, ns^c^	*k*_PF_, × 10^6^ s^–1^^d^	*k*_DF_, × 10^5^ s^–1^^e^	*k*_ISC_, × 10^7^ s^–1^^f^	*k*_RISC_, × 10^5^ s^–1^^g^
**1**	crystals	555	0.40	16.4	986.8	8.98	4.05	3.85	1.62
**2**	crystals	578	0.18	19.3	534.2	4.85	3.37	2.50	0.61
**1**	in mCP	544	0.50	21.4	645.5	11.1	7.68	2.44	3.81
**2**	in mCP	566	0.42	17.3	610.1	16.2	6.95	1.95	2.94

a Wavelength of emission maximum.

b Lifetime of prompt emission.

c Lifetime of delayed emission.

d Radiative rate constant of prompt fluorescence.

e Radiative rate constant of delayed fluorescence.

f Rate constant of ISC.

g Rate constant of RISC.

### 2.5 Photophysical Properties in the Solid State

**Figure 5:**
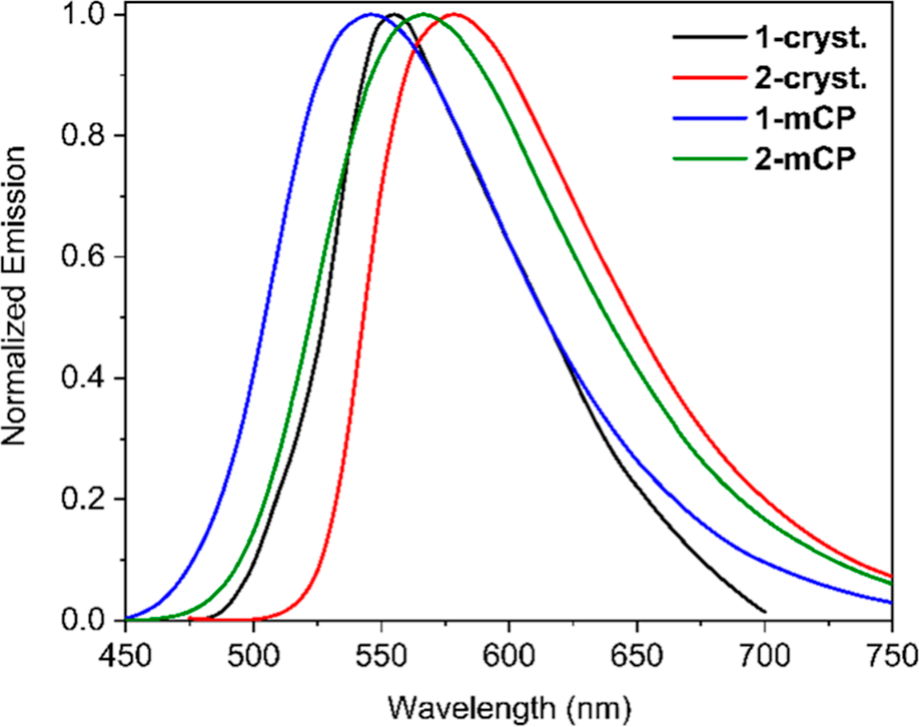
Photoluminescence spectra of dyes **1** and **2** in the crystalline state and of thin films of the dyes doped in mCP (20% of dye).

Then, we studied the photoluminescence properties of compounds **1** and **2** in the crystalline state. Crystals of dyes **1** and **2** show a broad emission peak maximized at 555 and 578 nm, respectively (Figure 5). The tested crystals of dyes **1** and **2** demonstrate one type of their conformers. This claim is supported by the similar time-resolved PL spectra at different times after excitation (Figure S21 in Supporting Information). In contrast, conformer-reach amorphous films are typically characterized by red-shifted time-resolved PL spectra with an increasing delay.^46^ The PLQY of crystalline compound **1** (0.40) is more than twice higher when compared to that of analogue **2** (0.18). Analogically to the solutions, in the crystalline state, both boron difluoride complexes demonstrate prompt and delayed lifetime decays (τ_PF_ = 16.4 ns, τ_DF_ = 986.8 ns for dye **1** and τ_PF_ = 19.3 ns, τ_DF_ = 534.2 ns for dye **2**). As a consequence, the radiative rate constants for prompt fluorescence are 8.98 × 10^6^ and 4.85 × 10^6^ s^–1^ for compounds **1** and **2**, respectively. Meanwhile the corresponding radiative rate constants (*k*_PF_) for delayed fluorescence are 4.05 × 10^5^ and 3.37 × 10^5^ s^–1^. This results in high values of rate constraint of ISC processes (*k*_ISC_ = 3.85 × 10^7^ and 2.50 × 10^7^ s^–1^ for dyes **1** and **2**, respectively, Table 2). However, the rate constant of RISC is much higher for crystal **1** (*k*_RISC_ = 1.62 × 10^5^ s^–1^) when compared with the crystalline sample of dye **2** (*k*_RISC_ = 0.61 × 10^5^ s^–1^). Evidently, the difference in photoluminescence efficiency of dyes **1** and **2** is caused by the geometrical orientation of the molecules in the crystalline state (see Figure 2).

Finally, to investigate the photoluminescence properties of dyes **1** and **2** in a semiconducting matrix, we examined the dye-doped thin films using *N*,*N*′-dicarbazolyl-3,5-benzene (mCP) as a host with 1:4 ratio of dye/mCP. In this matrix, the investigated emitters demonstrate hypsochromically shifted emission spectra (λ_em_ = 544 and 566 nm for dyes **1** and **2**, respectively, Figure 5) with increased PLQY values (50 and 42%, Table 2) when compared to those of crystalline samples. Additional hypsochromic shifts of PL spectra (λ_em_ = 518 and 552 nm for dyes **1** and **2**, respectively, Figure S19 in the Supporting Information) were observed when the concentration of guests was reduced to 1 wt % in an inert polymeric matrix ZEONEX. The mCP-based films exhibited biexponential fluorescence decays (τ_PF_ = 21.4 ns, τ_DF_ = 645.5 ns for dye **1** and τ_PF_ = 17.3 ns, τ_DF_ = 610.1 ns for dye **2**). In comparison with the crystals, the dye-doped films show enhanced values of *k*_PF_ (1.11 × 10^7^ and 1.62 × 10^7^ s^–1^ for dyes **1** and **2**, respectively) as well as *k*_DF_ (7.68 × 10^5^ and 6.95 × 10^5^ s^–1^ for compounds **1** and **2**, respectively) and, as a consequence, higher values of *k*_RISC_ (3.81 × 10^5^ and 2.94 × 10^5^ s^–1^ for dyes **1** and **2**, respectively). Additionally, we measured low-temperature photoluminescence and phosphorescence spectra of the investigated dye-doped mCP films (Figure S23 in Supporting Information). Both samples demonstrate a very small energy gap between the S_1_ and T_1_ energy levels (Δ*E*_ST_ = 0.06 eV for compound **1** and 0.08 eV for the cyano analogue **2**). These Δ*E*_ST_ values are in very good agreement with the values of activation energy (*E*_a_^RISC^) of RISC processes of dyes **1** (*E*_a_^RISC^ = 0.061 eV) and **2** (*E*_a_^RISC^ = 0.069 eV) obtained by the analyses of the rate constraint of RISC at different temperatures according to the Arrhenius dependence, respectively (Figure S24 in Supporting Information). The obtained data clearly indicate the efficient TADF properties of boron difluoride complexes **1** and **2**.

### 2.6 Theoretical Calculations

To confirm our previous assumptions and better understand the characteristics of the studied boron difluoride complexes **1** and **2**, we performed both ground-state (S_0_) and singlet- and triplet-excited state (S_1_, T_1_) structure simulations at the molecular level.

#### 2.6.1 Geometrical Properties

**Figure 6:**
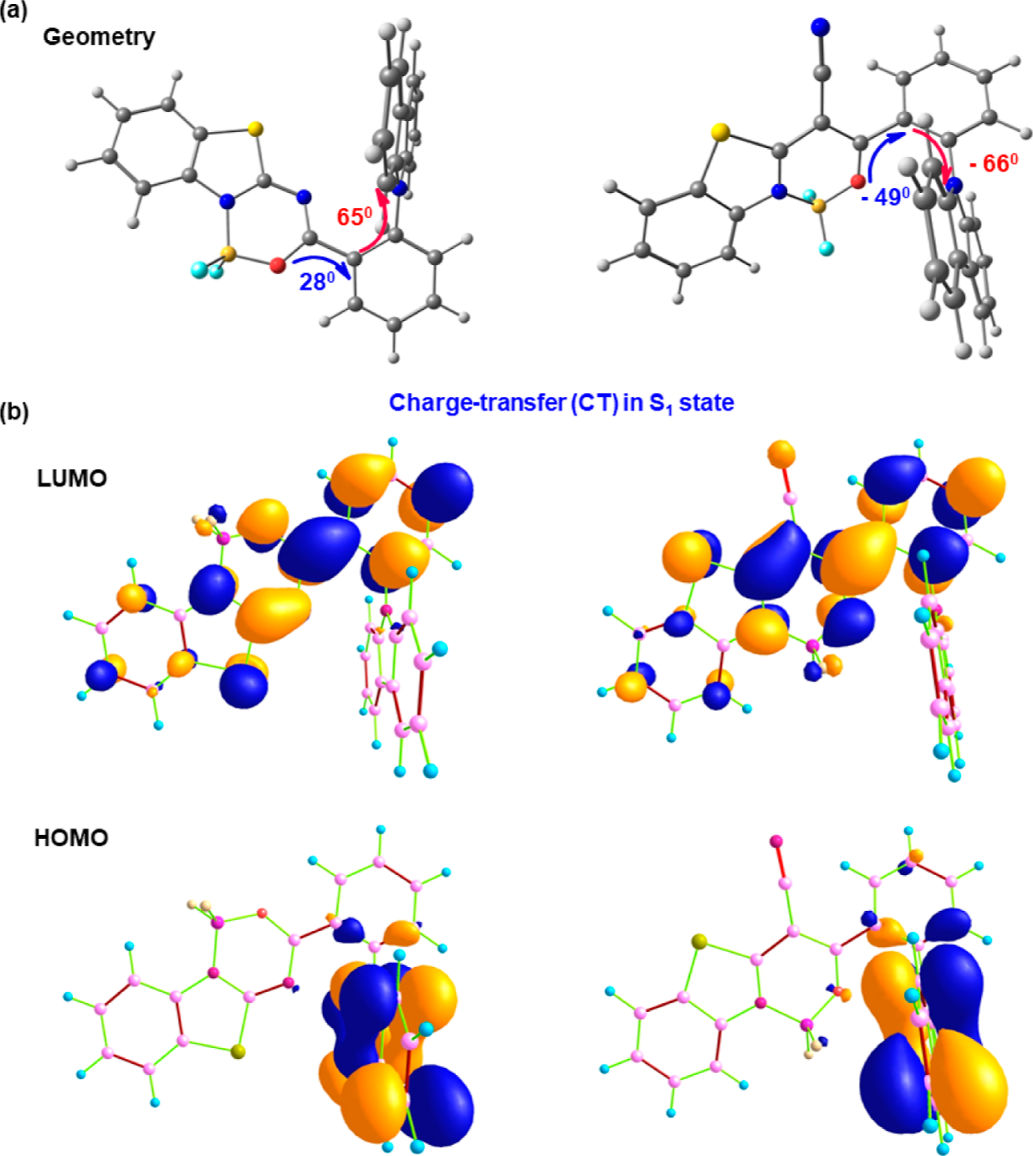
(a) DFT/B3LYP/6-31G(d)-level-simulated ground-state (S_0_) structures of boron complexes **1** (left) and **2** (right) with the computed twisting angles; (b) HOMO/LUMO charge-density distribution of the studied complexes showing the CT nature of singlet-excited S_1_ state at the TD-DFT/CAM-B3LYP/6-31G(d) theory. Herein, the calculated electronic HOMO/LUMO energy levels and HOMO–LUMO gap are −5.48/–2.43, 3.05 and −5.50/–2.49, 3.00 eV for boron complexes **1** and **2**, respectively.

Density functional theory (DFT) with a B3LYP hybrid functional and 6-31G(d) basis was used to simulate the S_0_ structure of the studied boron difluoride complexes (Figure 6). We observed that the carbazole donor is twisted with respect to the *ortho*-phenylene unit with almost identical angles in both molecules (65 and 66° for compounds **1** and **2**, respectively, Figure 6a). However, the presence of a cyano group in complex **2** led to more twisting of the acceptor unit in compound **2** (49°) as compared to molecule **1** (28°). The efficient twisting between the acceptor and donor molecules resulted in significant space separation between the highest occupied molecular orbital (HOMO) and lowest unoccupied molecular orbital (LUMO), and therefore results in the ICT character or the corresponding excited state (Figure 6b). We encountered nearly similar HOMO/LUMO energy levels and HOMO–LUMO energy gaps for complexes **1** and **2**. Thanks to the optimal tuning of the range-separated parameter (ω*) which is used in the time-dependent density functional theory (TD-DFT)/LC-ω* PBE/6-31+G(d) method, we calculated the singlet–triplet gap (Δ*E*_S1T1_) to be 0.035 and 0.121 eV for complex **1** and **2**, which successfully explains the TADF emission in the studied complexes.

#### 2.6.2 Photophysical Properties

The TD-DFT/CAM-B3LYP/6-31G(d) theory-calculated absorption and emission spectra were found to be in reasonable quantitative agreement with the experimental results. The lowest absorption and emission peaks of the studied boron difluoride complexes showed the CT nature of transition. The higher excitations such as S_0_ → S_2_ and S_0_ → S_3_ for complex **1** showed mixed CT and a locally excited emission (CT + LE) character, whereas for complex **2,** S_0_ → S_2_ and S_0_ → S_3_ excitations show LE and CT type nature, respectively (Table 3). In the UV region, the calculated strong absorption band for dye **1** corresponds to the S_0_ → S_3_ transition which is defined predominantly by HOMO – 2 → LUMO excitation with a small admixture of HOMO – 1 → LUMO electronic configurations, whereas for compound **2,** the strong absorption band is defined by S_0_ → S_2_ transition which possesses a single electronic configuration, i.e., HOMO – 2 → LUMO. The experimentally measured absorption near 425 nm for both complexes corresponds to the S_0_ → S_1_ transition identified as HOMO → LUMO single-electron excitation. The calculated emission in a toluene solution follows the experimental results. The presence of a cyano group greatly affected the emission properties of studied boron difluoride complexes. For instance, about 50 nm red-shifted emission is observed for complex **2** as compared to dye **1**, which matched the experiment well. In S_1_ excited state, the donor carbazole of complex **1** shows a comparatively larger twisting (−85.6°) than complex **2** (−65.3°), which results in a more pronounced CT character of complex **1** and hence results in emission with a decreased intensity (oscillator strength, *f* = 0.0009 for complex **1** vs *f* = 0.0129 for complex **2**).

**Table 3:** TD-DFT/CAM-B3LYP/6-31G(d) Theory-Level-Calculated Absorption (up to Third Singlet Excitations) and S_1_ → S_0_ Emission Energies (E), Wavelength (λ), and Oscillator Strength (*f*) of the Studied Dyes^a^

dye	absorption					S_1_ → S_0_ emission			
	excited state	E, eV	λ_abs_, nm	*f*	nature	*E*, eV	λ_em_, nm	*f*	nature
**1**	S_1_	3.31	375	0.0464	**CT**	2.66	465	0.0009	CT
	S_2_	3.94	314	0.1296	**CT** + LE				
	S_3_	4.05	306	0.6543	CT + **LE**				
**2**	S_1_	3.93	366	0.0108	**CT**	2.42	513	0.0129	CT
	S_2_	3.86	321	0.7467	**LE**				
	S_3_	3.97	312	0.0031	**CT**				

a The prominent nature of each transition has been marked bold. All calculations are in toluene solution state.

**Figure 7:**
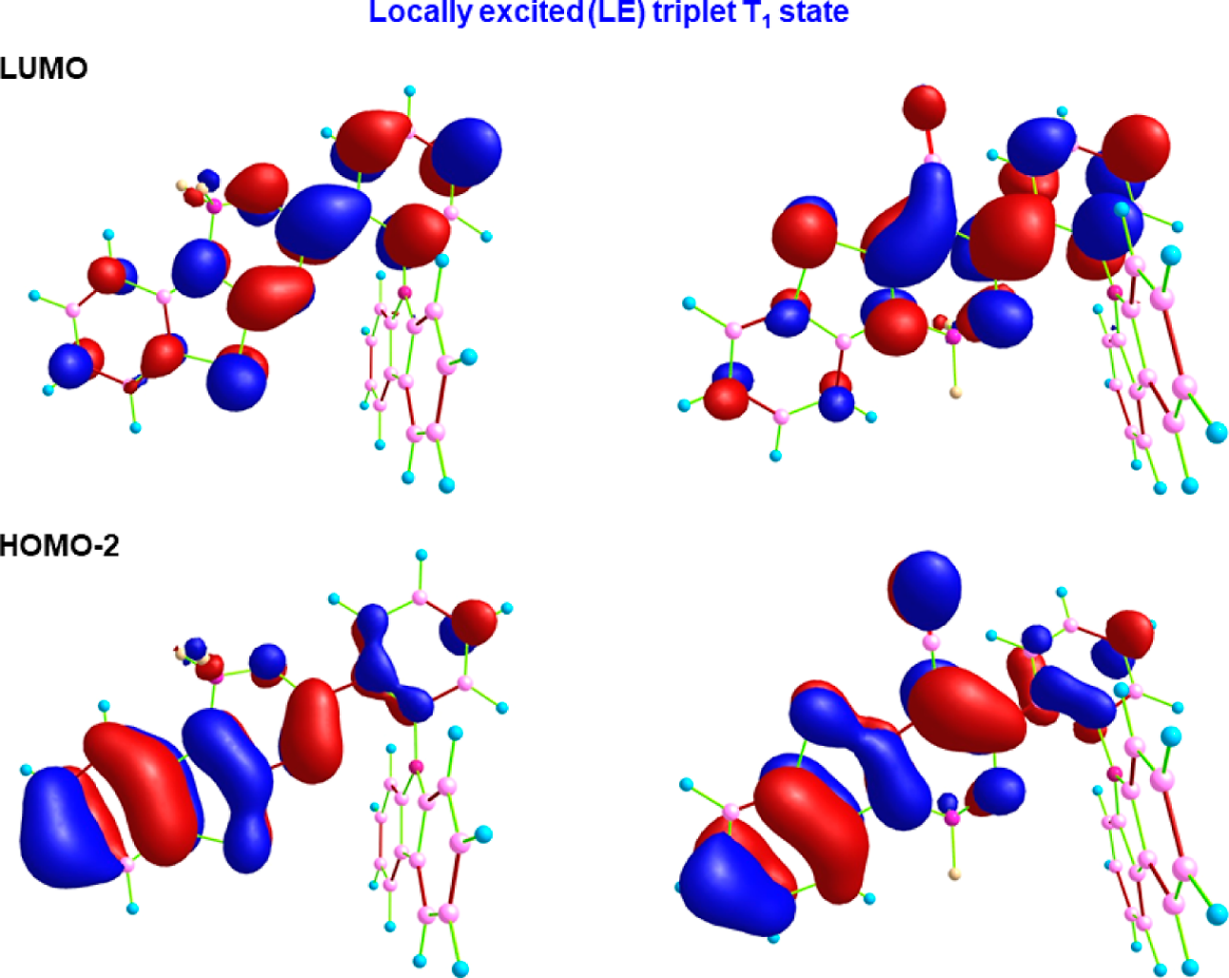
Calculated locally excited (LE) nature of lowest triplet T_1_ state of complexes **1** (left) and **2** (right). Here, T_1_ state is characterized by the major contribution from HOMO–2 → LUMO electronic transition.

A small singlet–triplet energy splitting (Δ*E*_ST_) and significantly large spin–orbit coupling (SOC) matrix elements between S_1_ and T_1_ states (SOCME_ST_) are known to result in efficient intersystem crossing, ISC (*k*_ISC_^S1T1^), and reverse ISC, RISC (*k*_RISC_^T1S1^) rates. With the optimally tuned range-separated method, we predicted sufficiently small Δ*E*_ST_ for both complexes (Table 4). Important to note is that the T_1_ state for both organoboron complexes sustains a predominantly LE character (Figure 7). Therefore, reduced Δ*E*_ST_ along with the coupling of ^1^CT singlet and ^3^LE triplet states (which successfully related to El-Sayed’s empirical rules) promoted the TADF behavior of the studied dyes. The predicted significantly larger SOCME_ST_ values at T_1_ state geometry such as 1.06 and 1.26 cm^–1^ for complexes **1** and **2** eventually resulted in higher RISC rates, i.e., 3.20 × 10^6^ and 0.658 × 10^6^ s^–1^, respectively. Herein, though complex **2** shows a larger SOCME_ST_, a relatively larger Δ*E*_ST_ value led to about 5 times decrease in the RISC rate. Moreover, for complex **1**, about 10 times larger RISC rate versus fluorescence decay rate (*k*_r_) successfully explains its efficient TADF emission as well as higher performance of the corresponding OLED (device A). However, in the case of complex **2**, we observed nearly comparable RISC and *k*_r_ rates, which are also sufficient for efficient TADF that explains only slightly lesser efficiency of device B OLED compared to device A.

**Table 4:** Calculated ISC and RISC Rates for Boron Difluoride Complexes **1** and **2**; SOCME Calculations at TD-DFT/PBE0/Triple-ζ Polarized (TZP) Theory Level, Adiabatic Excited-State Energies, Singlet–Triplet (S_1_–T_1_) Energy Gaps, and S_1_ → S_0_ Fluorescence Rates (*k*_*r*_) Were Computed by Using Optimized ω-Tuned LC-ω* PBE Functional and 6-31+G(d) Basis within TD-DFT Formalism^a^

dye	excited-state energies, eV	Δ*E*_ST_, eV	at S_1_ geometry				at T_1_ geometry		
			SOCME_ST_, cm^–1^	λ, eV	*k*r, × 10^6^ s^–1^	*k*_ISC_^S1T1^,× 10^7^ s^–1^	SOCME_ST_, cm^–1^	λ, eV	*k*_RISC_^S1T1^,× 10^6^ s^–1^
**1**	S_1_ = 2.22	0.035	0.09	0.016	0.30	1.34	1.06	0.336	3.20
	T_1_ = 1.97								
**2**	S_1_ = 2.02	0.121	0.21	0.067	1.34	2.88	1.26	0.321	0.658
	T_1_ = 1.69								

a All calculations were performed at room temperature and in toluene solvent medium.

### 2.7 Electroluminescence Properties

Encouraged by the intensive solid-state photophysical as well as charge-transporting properties of both boron difluoride complexes, we decided to apply these compounds in OLEDs. Guest–host systems were used as light-emitting layers of devices A and B, where boron dyes **1** and **2** were the guests and mCP was the host, respectively. The OLEDs were fabricated with the structures of ITO/CuI (8 nm)/m-MTDATA (35 nm)/TAPC (10 nm)/mCP:10 wt % emitters (20 nm)/PO-T2T (40 nm)/Ca (50 nm)/Al (200 nm), where copper(I) iodide (CuI),^47^ 4,4′,4″-tris[(3-methylphenyl)phenylamino]triphenylamine (*m*-MTDATA), and 2,4,6-tris[3-(diphenylphosphinyl)phenyl]-1,3,5-triazine (PO-T2T) were applied for the deposition of a hole-injecting layer, a hole-transporting layer (HTL), and a hole-blocking layer, respectively, while 1,1-bis[(di-4-tolylamino)phenyl]cyclohexane (TAPC) (Figure S25 in the Supporting Information) served as the exciton-/electron-blocking material due to its high LUMO energy level.

**Figure 8:**
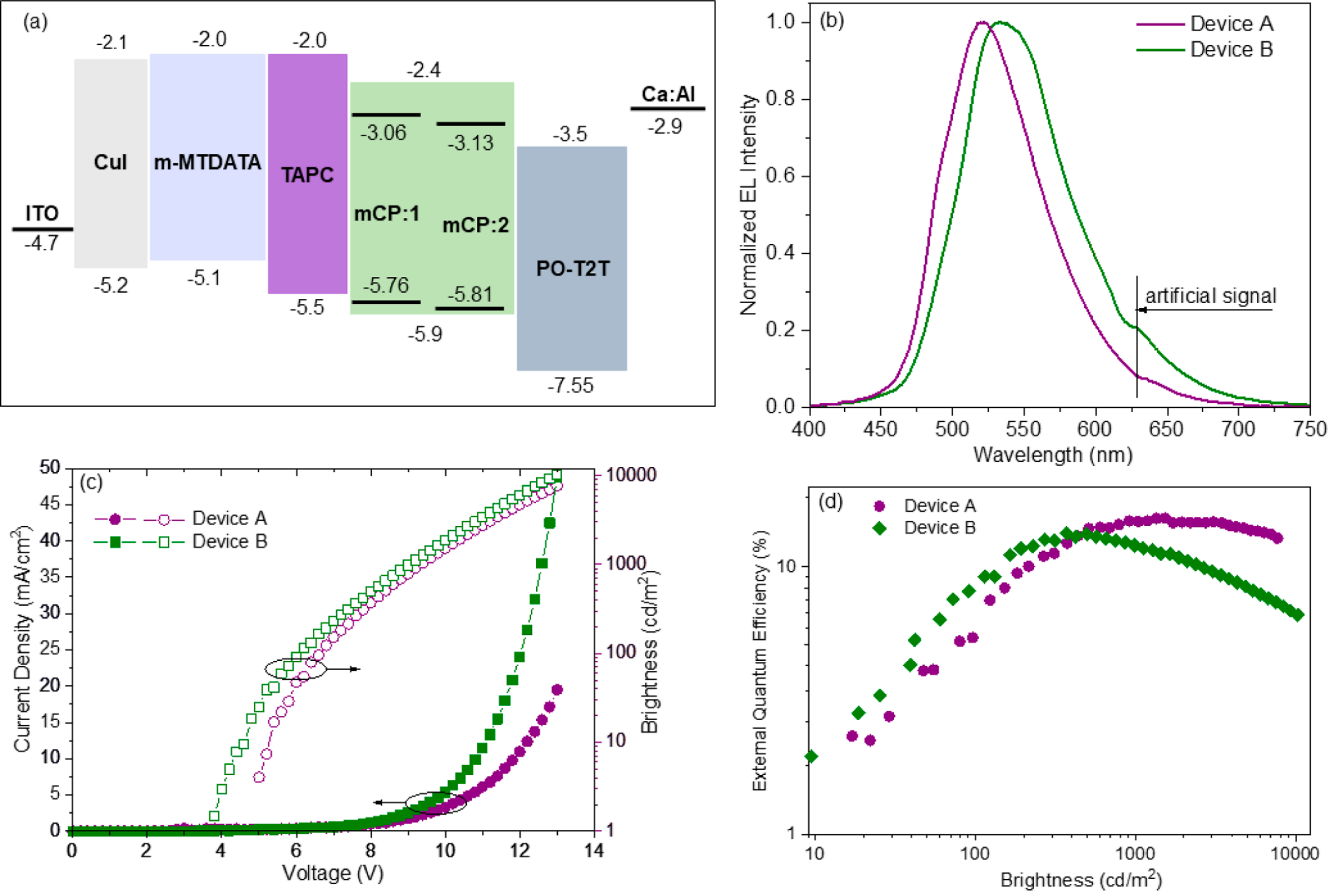
Electroluminescence properties of compounds **1** and **2**. Equilibrium energy diagram (a), EL spectra at constant voltage (b) of OLEDs. Brightness–voltage–current density curves (c) and quantum efficiency-brightness (d) of devices A and B.

Relatively low turn-on voltages were observed for devices A (5.3 V) and B (4.7 V) taken at 10 cd/m^2^ (Table 5). This observation indicates efficient injection from the electrodes as well as the transport of charge carriers to the emission layers. The brightness of the OLED structure B (10 300 cd/m^2^) exceeds the brightness of structure A (7692 cd/m^2^) under 12 V. Devices A and B were characterized by green electroluminescence (EL) with CIE color coordinates of (0.31, 0.51) and (0.32, 0.55), respectively. The electroluminescence (EL) spectra originated from the emission of dyes **1** and **2** (Figure 8b). The EL spectra (λ_EL_ = 525 and 534 nm for devices A and B, respectively) of devices are similar to the PL spectra of the crystals and films of host–guest boron dye/mCP mixtures (Figure 5). The shapes of the EL spectra of devices A and B reproduce the shapes of the PL spectra of the corresponding crystals of dyes **1** and **2** better than the PL spectra of the host-based films. This observation indicates that the electroluminescence of devices A and B mainly resulted from the most stable conformers of dyes **1** and **2**, which were detected by monocrystal analyses. EL spectra were not characterized by additional bands/shoulders that resulted from either the emissions of functional materials, interface exciplex (between mCP and PO-T2T at the interface of mCP/emitters/PO-T2T^48^), or electroplex of TAPC.^49^ Thus, very efficient injections of holes from HTL TAPC and electrons from ETL PO-T2T to the HOMO and LUMO levels of emitters occur following efficient light-emitting recombination. This claim is supported by the absence of energy barriers between HOMO–HOMO of TAPC and emitters and LUMO–LUMO of emitters and PO-T2T (Figure 8a). Since the electrons and holes are not blocked at the mCP/emitters/PO-T2T interface, the formation of the mCP:PO-T2T exciplex is not observed. When an electric voltage is applied to the OLED structure, direct recombination of electrons and holes on the guest component is supported by the similarity of PL and EL spectra of dyes **1** and **2**.

**Table 5:** Characteristics of OLEDs Containing Dyes **1** and **2**

dye	device	λ_EL_, nm^a^	*V*_on_, V^b^	*L*_max_, cd/m^2^^c^	C*E*_max_, cd/A^d^	P*E*_max_, lm/W^e^	EQE, %^f^	CIE^g^
**1**	A	525	5.3	7692	46.86	12.84	15.20/5.75/14.82	(0.31, 0.51)
**2**	B	534	4.7	10 302	42.34	13.88	13.29/8.57/12.00	(0.32, 0.55)

a Wavelength of electroluminescence maximum.

b Turn-on voltage at 1 cd/m^2^.

c Maximum luminance.

d Maximum current efficiencies.

e Maximum power efficiencies.

f External quantum efficiencies at maximum, 100, and 1000 cd/m^2^.

g Commission Internationale de l’Éclairage color coordinates measured at 100 cd/m^2^.

Because of the recombination of both singlet and triplet excitons formed under electrical excitation, devices A and B show maximum EQE values of 15.2 and 13.3%, respectively (Figure 8d, Table 5). The recombination of singlet and triplet excitons formed under electrical excitation is additionally proved by the transient EL measurements (Figure S26 in Supporting Information). The trend of EQE values well follows the trend of PLQY values and TADF properties of the tested emitters. Because the emitters were characterized by electron mobility much higher than their hole mobilities (Figure S10a in the Supporting Information), maximum EQE values were obtained at a relatively high brightness when the hole–electron balance was achieved. The different efficiency roll-offs of devices A and B can also be explained by the different mobility values of dyes **1** and **2**. For example, in comparison to device B, device A showed lower efficiency roll-offs due to ca. 2 times higher electron mobility of dye **1** in comparison to that of dye **2** at the same electric fields (1.5 × 10^–4^ cm^2^ V^–1^ s^–1^ for dye **1** versus 0.7 × 10^–4^ cm^2^ V^–1^ s^–1^ for dye **2** at an electric field of 1.35 × 10^6^ V/cm). The high EQE values of 15.2 and 13.3% well support the TADF properties of dyes **1** and **2** and highlight the perspectives of benzothiazole-based boron difluoride complexes for electroluminescence applications.

## 3 Conclusions

We designed, facilely synthesized, and studied two donor–acceptor boron difluoride complexes **1** and **2** constructed of a carbazole electron donor and benzo[4,5]thiazolo[3,2-*c*][1,3,5,2]oxadiazaborinine or benzo[4,5]thiazolo[3,2-*c*][1,3,2]oxazaborinine-4-carbonitrile acceptor units, while the donor and acceptor are directly combined as substituents at the *ortho*-phenylene-linked position of a benzene ring. Due to the presence of numerous hydrogen-bonding intermolecular interactions, these dyes exhibit intense solid-state luminescence. The geometrical difference in the acceptor units causes the higher PLQY of dye **1** with almost an orthogonal acceptor orientation relative to the carbazole donor plane. Both dyes demonstrated efficient AIE and TADF properties. They also showed pertinent charge-injecting and charge-transporting properties for electronic applications, reaching an electron mobility of 1.5 × 10^–4^ cm^2^ V^–1^ s^–1^ at an electric field of 1.35 × 10^6^ V/cm in the best experimental case. The application of these emitters in OLEDs results in external quantum efficiencies of 15 and 13%. Our work sheds new light on the design of *ortho*-phenylene-linked donor–acceptor TADF materials.

## 4 Experimental Section

### 4.1 General

All reagents and chemicals were purchased from commercial sources (abcr, TCI, Acros Organics, FluoroChem) and used without further purification.

### 4.2 Instrumental Methods

NMR spectra were recorded on a Varian V NMRS 500 MHz (at 500, 125, and 470 MHz for ^1^H, ^13^C, and ^19^F NMR spectra, respectively) or a Varian Mercury 400 MHz (at 400 and 100 MHz for ^1^H and ^13^C NMR spectra, respectively) spectrometer at room temperature using CDCl_3_ or DMSO-*d*_6_ as a solvent and referenced externally to SiMe_4_. The singlet, doublet, triplet, and multiplet signals are indicated as “s”, “d”, “t”, or “m”, respectively. High-resolution mass spectra were collected on a Synapt G2-S HDMS (Waters Inc.) mass spectrometer equipped with a q-TOF-type mass analyzer and an electrospray ion source. The instrument was controlled, and recorded data were processed using the MassLynx 4.1 software package (Waters Inc., USA).

Single crystals of boron complexes were grown by slow evaporation of their solutions in a hexane/DCM (2:1) mixture (structures **1**) or cyclohexane/DCM (1:1) mixture (structure **2**). The X-ray experiments were performed on a SuperNova Agilent diffractometer using Cu Kα (λ = 1.54184 Å) radiation at 100 K. Data reduction was done with CrysAlisPro.^50^ The obtained structures were solved by direct methods and refined using SHELXL^51^ under WinGX.^52^ The disordered solvent in the crystal structure of compound **1** was removed by SQUEEZE. Crystallographic data of compounds **1** and **2** have been deposited with the Cambridge Crystallographic Data Centre (CCDC) and can be obtained, free of charge, from CCDC via https://www.ccdc.cam.ac.uk/structures/.

TGA experiments were performed on a TGA/DSC 3+, Mettler Toledo apparatus at a heating rate of 5 °C/min under a nitrogen atmosphere. DSC measurements were done on DSC 3, Mettler Toledo equipment at a heating rate of 5 °C/min under nitrogen atmosphere.

Electrochemical measurements were achieved using a mAUTOLAB Type III apparatus, platinum coil, glassy carbon, and silver wire as auxiliary, working, and reference electrodes, respectively.

The UPS measurements of IP values of vacuum-deposited layers were carried out by using a home-made UPS setup in the air. This setup consisted of a holder of UPS samples, a 6517B Keithley electrometer, a UV deuterium lamp ASBN-D130-CM, and a CM110 1/8 m monochromator. The samples were vacuum-deposited onto glass substrates with an electrode [fluorine-doped tin oxide]. The UPS setup allowed recording photocurrent (*i*) under different excitation wavelengths [photon energies (*h*ν)]. The photocurrent was collected under a constant electric field of 1 × 103 V/cm (300 V was applied to the counter electrode, and the distance between the electrodes was 3 mm). The dependences (UPS spectra) of the square root on photon energies were plotted to estimate the IP values of the vacuum-deposited layers. These values were obtained by extrapolating the linear part of the *i*-dependences to the baseline (at *i* equals zero).

UV–vis absorption spectra of ca. 10^–5^ M solutions of the dyes were recorded at room temperature using a Shimadzu UV2700 spectrophotometer. Steady-state and time-resolved photoluminescence spectra of the samples (solutions with the concentration of ca. 1 × 10^–5^ M, films, and crystals) were recorded by using a spectrometer FLS980, Edinburgh Instruments. The time-resolved PL spectra and PL decay curves were recorded by using a pulsed diode laser (PDL 820, PicoQuant, λ_ex_ = 374 nm). The absolute PLQY values were recorded using an FLS980 spectrometer with an integrating sphere in the air.

In the case of AIE measurements, the THF/water mixtures of various ratios were prepared by slowly adding distilled water into solutions of compound **1** or **2** in THF, while the concentration was achieved at 2.5 × 10^–6^ M. The absorption and photoluminescence measurements of such obtained samples were conducted immediately.

### 4.3 Synthesis

#### 2-(Benzo[*d*]thiazol-2-yl)acetonitrile (**4**)

2-(Benzo[*d*]thiazol-2-yl)acetonitrile (**4**) was prepared using a modified literature procedure.^53^ A solution of 2-aminothiophenol (1.00 g, 7.99 mmol) in ethanol (5 mL) was added to a stirred solution of malononitrile (0.53 g, 7.99 mmol) and acetic acid (0.46 mL, 7.99 mmol) in ethanol (5 mL). The reaction mixture was stirred for 20 min at room temperature and for 30 min at reflux. After cooling, the solvent was evaporated under reduced pressure, water (5 mL) was added, and the formed precipitate was filtered, washed with cold ethanol, and dried to give compound **4** (1.20 g, 6.89 mmol, 86%) as a yellow solid. ^1^H NMR (500 MHz, CDCl_3_): δ 8.04 (1H, d, *J* = 8.1 Hz, Ar–H), 7.89 (1H, d, *J* = 8.0 Hz, Ar–H), 7.52 (1H, ddd, *J* = 8.1 Hz, *J* = 7.3 Hz, *J* = 1.2 Hz, Ar–H), 7.44 (1H, ddd, *J* = 8.0 Hz, *J* = 7.3 Hz, *J* = 1.1 Hz, Ar–H), 4.23 (2H, s, CH_2_) ppm; ^13^C{H} NMR (125 MHz, CDCl_3_): δ 158.20, 152.79, 135.42, 126.69, 125.94, 123.36, 121.70, 114.84, 23.17 ppm.

#### Methyl 2-(9*H*-Carbazol-9-yl)benzoate (**7**)

Methyl 2-(9H-carbazol-9-yl)benzoate (**7**) was synthesized using a modified literature procedure.^54^ The mixture of ethyl 2-iodobenzoate (**6**, 1.00 mL, 5.98 mmol, 1.0 equiv), carbazole (**5**, 1.30 g, 7.77 mmol, 1.0 equiv), potassium carbonate (3.20 g, 23.32 mmol, 3.0 equiv), copper(I) iodide (148 mg, 0.78 mmol, 0.1 equiv), and 1,10-phenanthroline (140 mg, 0.78 mmol, 0.1 equiv) in dimethylacetamide (15 mL) was refluxed for 24 h under argon. After cooling, the mixture was filtered through silica gel, eluted with hexane/ethyl acetate (8:1), and concentrated. The residue was separated by column chromatography on silica gel (hexane, next hexane/AcOEt = 98:2) to afford pure product **7** (1.55 g, 5.14 mmol, 66%) as a white powder. ^1^H NMR (500 MHz, CDCl_3_): δ 8.11–8.18 (3H, m, Ar–H), 7.76 (1H, ddd, *J* = 9.2 Hz, *J* = 7.6 Hz, *J* = 1.6 Hz, Ar–H), 7.58–7.63 (2H, m, Ar–H), 7.40 (2H, dd, *J* = 8.7 Hz, *J* = 7.6 Hz, Ar–H), 7.29 (2H, dd, *J* = 8.2 Hz, *J* = 7.6 Hz, Ar–H), 7.15 (2H, d, *J* = 8.2 Hz, Ar–H), 3.21 (3H, s, OCH_3_) ppm; ^13^C{H} NMR (125 MHz, CDCl_3_): δ 166.40, 141.60 (2C), 136.93, 133.32, 131.97, 130.10, 130.08, 128.26, 125.92 (2C), 123.27 (2C), 120.24 (2C), 119.77 (2C), 109.27 (2C), 52.05 ppm. HRMS (ESI-TOF) calcd for C_20_H_15_NO_2_Na [M + Na]^+^, 324.1000; found, 324.0998.

#### 4.5 2-(9*H*-Carbazol-9-yl)benzoic Acid (**8**)

The mixture of ester **7** (1.45 g, 4.81 mmol, 1 equiv), THF (25 mL), ethanol (5 mL), water (5 mL), and KOH (0.81 g, 15.44 mmol, 3 equiv) was refluxed for 12 h and then cooled to room temperature. The solvents were evaporated, and water (5 mL) and 5 N HCl aqueous solution were slowly added until pH ∼ 5. The mixture was left in a fridge for 3 h. The formed precipitate was filtered, washed with water, and dried to give acid **8** (1.38 g, ∼100%) as a white powder. ^1^H NMR (400 MHz, DMSO-*d*_6_): δ 8.21 (2H, d, *J* = 8.2 Hz, Ar–H), 8.04 (1H, dd, *J* = 7.8 Hz, *J* = 1.5 Hz, Ar–H), 7.81 (1H, ddd, *J* = 9.0 Hz, *J* = 7.8 Hz, *J* = 1.5 Hz, Ar–H), 7.67 (1H, ddd, *J* = 9.0 Hz, *J* = 7.8 Hz, *J* = 1.0 Hz, Ar–H), 7.57 (1H, dd, *J* = 7.8 Hz, *J* = 1.0 Hz, Ar–H), 7.37 (2H, ddd, *J* = 8.2 Hz, *J* = 7.6 Hz, *J* = 1.1 Hz, Ar–H), 7.24 (2H, ddd, *J* = 8.2 Hz, *J* = 7.6 Hz, *J* = 0.8 Hz, Ar–H), 7.08 (2H, d, *J* = 8.2 Hz, Ar–H) ppm; ^13^C{H} NMR (100 MHz, DMSO-*d*_6_): δ 166.95, 141.10 (2C), 135.39, 132.93, 132.19, 131.23, 129.75, 128.64, 125.98 (2C), 122.64 (2C), 120.32 (2C), 119.52 (2C), 109.37 (2C) ppm. HRMS (ESI-TOF) calcd for C_19_H_12_NO_2_ [M – H]^−^: 286.0868, found: 286.0869.

#### 4.6 *N*-(Benzo[*d*]thiazol-2-yl)-2-(9H-Carbazol-9-yl)benzamide (**10**)

Thionyl chloride (237 μL, 3.27 mmol, 1.5 equiv) was added to a suspension of 2-(9*H*-carbazol-9-yl)benzoic acid (**8**, 626 mg, 2.18 mmol, 1 equiv) in dry toluene (30 mL). The reaction mixture was heated at 110 °C for 1 h and then cooled to room temperature. The solvent and the rest of SOCl_2_ were evaporated in a vacuum. The as-obtained benzoyl chloride **9** was dissolved in dry 1,4-dioxane (30 mL). Benzo[*d*]thiazol-2-amine (**3**, 344 mg, 2.29 mmol, 1.05 equiv), dry triethylamine (912 μL, 6.54 mmol, 3.0 equiv), and DMAP (13 mg, 0.11 mmol, 0.05 equiv) were added. The reaction mixture was refluxed for 24 h and then cooled to room temperature. Water (30 mL) and the saturated aqueous solution of NaHCO_3_ (30 mL) were added, and the mixture was extracted with DCM (3 × 50 mL). The combined organic layers were dried over anhydrous Na_2_SO_4_, filtered, and concentrated. The crude product was purified by flash column chromatography on silica gel (hexane/dichloromethane from 4:1 to 1:3, v/v) to afford pure compound **10** (585 mg, 1.39 mmol, 64%) as a white powder. ^1^H NMR (500 MHz, CDCl_3_): δ 11.48 (1H, br s, NH), 8.09 (2H, dd, *J* = 8.0 Hz, *J* = 1.5 Hz, Ar–H), 7.95 (1H, d, *J* = 7.8 Hz, Ar–H), 7.71 (1H, dd, *J* = 7.8 Hz, *J* = 7.5 Hz, Ar–H), 7.67 (1H, d, *J* = 7.8 Hz, Ar–H), 7.58 (1H, d, *J* = 7.8 Hz, Ar–H), 7.54 (1H, dd, *J* = 7.7 Hz, *J* = 7.6 Hz, Ar–H), 7.19–7.27 (4H, m, Ar–H), 7.17 (1H, dd, *J* = 7.6 Hz, *J* = 7.4 Hz, Ar–H), 7.05–7.13 (3H, m, Ar–H), 6.70 (1H, d, *J* = 8.1 Hz, Ar–H) ppm; ^13^C{H} NMR (125 MHz, CDCl_3_): δ 164.79, 158.84, 147.35, 140.80 (2C), 136.25, 133.41, 131.72, 131.39, 131.37, 129.32, 128.40, 126.33 (2C), 125.76, 123.80 (2C), 123.61, 121.15, 120.68 (2C), 120.30 (2C), 119.86, 109.46 (2C) ppm. HRMS (ESI-TOF) calcd for C_26_H_17_N_3_OSNa [M + Na]^+^: 442.0990; found: 442.0992.

#### 4.7 3-(2-(9H-Carbazol-9-yl)phenyl)-2-(benzo[*d*]thiazol-2-yl)-3-oxopropanenitrile (**11**)

Thionyl chloride (204 μL, 2.81 mmol, 1.5 equiv) was added to a suspension of 2-(9*H*-carbazol-9-yl)benzoic acid (**8**, 538 mg, 1.87 mmol, 1.0 equiv) in dry toluene (25 mL). The reaction mixture was heated at 110 °C for 1 h. After cooling, the solvent and the rest of SOCl_2_ were evaporated in a vacuum. The as-obtained benzoyl chloride **9** was dissolved in dry 1,4-dioxane (25 mL). 2-(Benzo[*d*]thiazol-2-yl)acetonitrile (**4**, 326 mg, 1.87 mmol, 1.0 equiv) and dry triethylamine (783 μL, 5.62 mmol, 3.0 equiv) were added. Next, the reaction mixture was stirred at 100 °C for 24 h. After cooling, water (25 mL) and the saturated aqueous solution of NaHCO_3_ (30 mL) were added, and the mixture was extracted with DCM (3 × 40 mL). The combined organic layers were dried over anhydrous Na_2_SO_4_, filtered, and concentrated. The crude product was purified by flash column chromatography on silica gel (hexane/dichloromethane from 4:1 to 1:3, v/v) to afford pure compound **11** (516 mg, 1.16 mmol, 62%) as a white powder. ^1^H NMR (500 MHz, DMSO-*d*_6_): δ 13.54 (1H, br s, NH), 8.13 (2H, d, *J* = 7.7 Hz, Ar–H), 7.85 (1H, d, *J* = 7.5 Hz, Ar–H), 7.79 (1H, d, *J* = 9.1 Hz, Ar–H), 7.76 (1H, dd, *J* = 7.7 Hz, *J* = 1.3 Hz, Ar–H), 7.71 (1H, dd, *J* = 7.4 Hz, *J* = 7.2 Hz, Ar–H), 7.53–7.62 (2H, m, Ar–H), 7.42 (1H, dd, *J* = 7.8 Hz, *J* = 7.6 Hz, Ar–H), 7.35 (2H, dd, *J* = 7.7 Hz, *J* = 7.5 Hz, Ar–H), 7.26 (1H, dd, *J* = 7.7 Hz, *J* = 7.6 Hz, Ar–H), 7.22 (2H, dd, *J* = 8.2 Hz, Ar–H), 7.19 (2H, dd, *J* = 7.5 Hz, *J* = 7.4 Hz, Ar–H) ppm; ^13^C{H} NMR (125 MHz, DMSO-*d*_6_): δ 185.13, 166.73, 141.12 (2C), 138.77, 138.23, 134.34, 131.59, 129.27, 129.23, 128.37, 127.50, 126.72, 125.68 (2C), 124.28, 122.70 (2C), 122.57, 120.17 (2C), 119.73 (2C), 118.55, 114.10, 110.05 (2C), 77.05 ppm. HRMS (ESI-TOF) calcd for C_28_H_16_N_3_OS [M – H]^−^: 442.1014, found: 442.1017.

#### 3-(2-(9*H*-Carbazol-9-yl)phenyl)-1,1-difluoro-1*H*-1λ,10λ^4^-benzo[4,5]thiazolo[3,2-*c*][1,3,5,2]oxadiazaborinine (**1**)

To a suspension of ligand **10** (520 mg, 1.24 mmol) in dry DCM (30 mL) was added distilled DIPEA (4.32 mL, 24.79 mmol) under an argon atmosphere. The mixture was stirred for 15 min at room temperature. Then, BF_3_·Et_2_O (1.53 mL, 12.40 mmol) was added, and the reaction mixture was stirred for 24 h at room temperature. After that, water (50 mL) was added, and the mixture was extracted with DCM (3 × 40 mL). The collected organic phases were dried over anhydrous Na_2_SO_4_, filtered, and concentrated. The crude product was purified by column chromatography on silica gel (hexane/dichloromethane from 8:1 to 2:1, v/v) to afford pure compound **1** (429 mg, 0.92 mmol, 74%) as a yellow powder. ^1^H NMR (500 MHz, CDCl_3_): δ 8.53 (1H, dd, *J* = 8.1 Hz, *J* = 1.6 Hz, Ar–H), 8.18 (2H, d, *J* = 7.6 Hz, Ar–H), 7.88 (1H, ddd, *J* = 7.6 Hz, *J* = 7.5 Hz, *J* = 1.6 Hz, Ar–H), 7.85 (1H, d, *J* = 8.5 Hz, Ar–H), 7.69–7.74 (2H, m, Ar–H), 7.57 (1H, d, *J* = 8.1 Hz, Ar–H), 7.47 (1H, dd, *J* = 7.7 Hz, *J* = 7.5 Hz, Ar–H), 7.31–7.38 (3H, m, Ar–H), 7.25 (2H, dd, *J* = 7.6 Hz, *J* = 7.2 Hz, Ar–H), 7.10 (2H, d, *J* = 8.1 Hz, Ar–H) ppm; ^13^C{H} NMR (125 MHz, CDCl_3_): δ 172.89, 165.92, 142.20 (2C), 139.61, 138.49, 134.84, 133.32, 131.46, 129.90 128.84, 128.20, 127.50, 126.39, 125.94 (2C), 123.45 (2C), 121.93, 120.13 (2C), 119.70 (2C), 118.57, 109.38 (2C) ppm; ^19^F NMR (470 MHz, CDCl_3_): δ −135.89 (2F, m, BF_2_) ppm. HRMS (ESI-TOF): calcd for C_26_H_16_BN_3_OF_2_SNa [M + Na]^+^, 490.0973; found, 490.0971.

#### 4.8 3-(2-(9*H*-Carbazol-9-yl)phenyl)-1,1-difluoro-1*H*-1λ,^4^10λ^4^-benzo[4,5]thiazolo[3,2-*c*][1,3,2]oxazaborinine-4-carbonitrile (**2**)

To a suspension of ligand **11** (470 mg, 1.06 mmol) in dry DCM (35 mL) was added distilled DIPEA (3.69 mL, 21.19 mmol) under an argon atmosphere. The mixture was stirred for 15 min at room temperature. Then, BF_3_·Et_2_O (1.31 mL, 10.60 mmol) was added, and the reaction mixture was stirred for 24 h at room temperature. After that, water (50 mL) was added, and the mixture was extracted with DCM (3 × 40 mL). The collected organic phases were dried over anhydrous Na_2_SO_4_, filtered, and concentrated. The crude product was purified by column chromatography on silica gel (hexane/dichloromethane from 8:1 to 1:2, v/v) to afford pure compound **2** (365 mg, 0.74 mmol, 70%) as an orange powder. ^1^H NMR (500 MHz, CDCl_3_): δ 8.08 (2H, d, *J* = 7.7 Hz, Ar–H), 7.98–8.01 (2H, m, Ar–H), 7.82 (1H, ddd, *J* = 8.9 Hz, *J* = 7.7 Hz, *J* = 1.4 Hz, Ar–H), 7.75 (1H, d, *J* = 8.1 Hz, Ar–H), 7.66–7.71 (2H, m, Ar–H), 7.55 (1H, ddd, *J* = 8.8 Hz, *J* = 7.9 Hz, *J* = 0.9 Hz, Ar–H), 7.46 (1H, dd, *J* = 7.6 Hz, *J* = 7.4 Hz, Ar–H), 7.39 (2H, ddd, *J* = 8.1 Hz, *J* = 7.7 Hz, *J* = 0.9 Hz, Ar–H), 7.33 (2H, d, *J* = 8.1 Hz, Ar–H), 7.24 (2H, dd, *J* = 7.7 Hz, *J* = 7.4 Hz, Ar–H) ppm; ^13^C{H} NMR (125 MHz, CDCl_3_): δ 175.34, 166.55, 142.37, 141.13 (2C), 136.87, 133.88, 131.38, 130.99, 129.72, 128.85, 128.48, 128.24, 127.14, 126.04 (2C), 123.94 (2C), 122.05, 120.50 (2C), 120.21 (2C), 119.30 (t, *J*_C–F_ = 2.7 Hz), 113.92, 110.03 (2C), 83.41 ppm; ^19^F NMR (470 MHz, CDCl_3_): δ −134.66 (2F, m, BF_2_) ppm. HRMS (ESI-TOF) calcd for C_28_H_16_BN_3_OF_2_SNa [M + Na]^+^, 514.0973; found, 514.0974.

### 4.9 Charge-Transporting Properties

TOF setup was constructed to investigate the charge-transport properties of the films. The films were deposited onto ITO-covered glass substrates obtained from Ossila at 3 × 10^–6^ mBar using the method of thermal evaporation. Then, the films were covered by an aluminum electrode using shadow masks from Ossila. The active area of TOF samples (ITO/film/Al) was of 4.6 mm^2^. The main parts of the TOF setup were a laser (EKSPLA, NL300), an oscilloscope (Tektronix, TDS 3032C), and an electrometer (Keithley, 6517B). It was possible to record photocurrent transients for holes or electrons in the tested films under different electric fields. The photocurrent transients built in log–log scales were used to obtain the charge transit times (*t*_tr_) under different voltages (*V*) (Figure S10b-d in the Supporting Information). The formula μ = *d*^2^/(*V* × *t*_tr_) was used to calculate the charge mobility values. The thicknesses (*d*) of the films were measured using a Profilm3D profilometer (Figures S11 and S12 in Supporting Information).

### 4.10 Theoretical Calculations

DFT with B3LYP hybrid functional and 6-31G(d) basis has been used for S_0_ geometry optimization of complexes **1** and **2** in the toluene solution state.^55^ Here, we implemented the polarizable continuum model^56^ to consider the condensed phase simulation in the toluene solvent (ε = 2.3741) and to validate with the experimental results. With the lowest-energy conformation S_0_ geometry in hand, we then carried out S_1_ and T_1_ state simulations using the TD-DFT^57^ framework with long-range-corrected Coulomb-attenuating method (CAM-B3LYP)^58^ functional. The reason for using CAM-B3LYP is because this functional has been found better in describing the CT character of excited states in donor–acceptor-based TADF systems.^59^

We followed the work by Brédas et al.^60^ and Kronik et al.^61^ and implemented here the range-separated functional method which has been found successful in terms of computational costs as well as reliable estimation of excited-state energies and the lowest singlet–triplet energy gaps. We used the optically tuned LC-ω* PBE functional with the 6-31+G(d) basis function within the TD-DFT framework to estimate the excitation energies of the studied compounds. At the ground-state geometry, we used the LC-ω PBE functional^62^ with the 6-31+G(d) basis set to compute the optimal ω values which were 0.180 for both dyes **1** and **2**. It should be noted that the ω parameter was optimized in gas-phase medium.

In this context, the tuning of range-separated ω parameter was performed by minimizing the following equation^59,60^
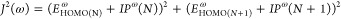
1

For a specific choice of ω, *E*_HOMO(*N*)_^ω^ and *E*_HOMO(*N*+1)_^ω^ are HOMO energies of *N* and *N* + *1* electron systems, respectively. The IP’s IP^ω^(*N*) and IP^ω^(*N* + 1), therefore, can be calculated as the energy difference as follows
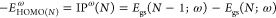
2
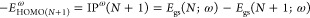
3

Herein, for the evaluation of eqs 1–3, we computed all of the single-point energy calculations for *N* and *N* + *1* electron systems using the LC-ω PBE/6-31+G(d) theory for each range-separated ω parameter. For the calculated optimized ω* value of each compound, we obtained coincidence in between the left and right parts of eqs 2 and 3, which successfully explains the optimal tuning of the range-separated parameter.

All of the abovementioned quantum-chemical calculations have been performed using Gaussian 16. B.01 package.^63^

The crucial component to describe the TADF process between the S_1_ and T_1_ states is the RISC rate (*k*_RISC_^*T*1*S*1^). This RISC factor is mainly driven by the direct SOC which is the only electronic coupling between the S_1_ and T_1_ states. Within the scalar relativistic approximation, we used TZP^64^ basis set with no frozen core, PBE0 functional^65^ to calculate the perturbative SOC (pSOC). The calculations were performed at the TD-DFT level using the Amsterdam density functional ADF2023 package.^66^ Here, it is pointed out that the singlet S_1_ state geometries were considered for direct ISC rate (*k*_ISC_^*S*1*T*1^) calculations, whereas the RISC rate calculations were carried out at the relevant triplet T_1_ geometry. Additionally, the conductor-like screening model^67^ continuum solvation approach was considered to analyze the matrix effects. Herein, the SOC matrix elements were calculated as root-mean-squares that means square root of the sum of squares of SOCME’s of all sublevels of the unoccupied states, i.e., ,^68^ where *m* is the relevant triplet-state sublevel.

We implemented the Fermi Golden Rule to compute RISC and ISC rate constants, which is as follows^69^
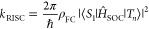
4where the second term has been defined before, and the first term ρ_FC_ is the Franck–Condon-weighted density of states, which can be calculated using the Marcus theory as
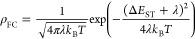
5where λ denotes the reorganization energy. For RISC rate, λ can be calculated as the difference of the energy of S_1_ state at relevant T_1_ state geometry and energy of S_1_ state at S_1_ geometry, i.e., λ = *E*_S1/T1_ – *E*_S1/S1_. Similarly, ISC rate is computed as the difference between the energy of the relevant T_1_ state at S_1_ state geometry and the energy of the relevant T_1_ state at T_1_ geometry, *i.e.,* λ = *E*_T1/S1_ – *E*_T1/T1_. Here, Δ*E*_ST_ < 0 for the ISC rate, and it has the same value with opposite sign for the RISC rate, i.e., Δ*E*_ST_ > 0.

We used the Strickler–Berg equation,^70^, where *f* is the calculated oscillator strength, and  defines the de-excitation energy to calculate the fluorescence emission rates.

### 4.11 Device Fabrications

OLEDs were formed by thermal deposition in a vacuum (10^–5^ Torr) on a glass substrate with a transparent conductive layer of ITO, by stepwise deposition of a hole–injection layer of CuI, two hole-transporting layers (*m*-MTDATA and TAPC) along with an electron-transporting layer (PO-T2T). Compounds **1** and **2** were doped in mCP, and the mCP:**1** and mCP:**2** layers were formed by controlled simultaneous deposition from two sources. Calcium and aluminum were used as cathode materials with a low work function, providing good electron injection to the electron-transport layer. The active area of the obtained devices was 6 mm^2^. The density–voltage and luminance–voltage characteristics were measured by using a semiconductor parameter analyzer HP4145A. The measurement of brightness was obtained using a calibrated photodiode, and the electroluminescence spectra were recorded with an Ocean Optics USB2000 spectrometer.

## Supporting Information Available

ORTEP diagrams for the X-ray structures and crystal data of complexes; TGA curves; DSC thermograms; cyclic voltammograms; charge carrier mobility curves; fluorescence decays of dyes; additional photophysical data; and NMR spectra of all synthesized compounds (PDF)
Crystal data of **1** (CIF)
Crystal data of **2** (CIF)

## Supplementary Material

am4c12662_si_001.pdf

am4c12662_si_002.cif

am4c12662_si_003.cif

## Abbreviations

TADF: thermally activated delayed fluorescence
EQE: external quantum efficiency
OLED: organic light-emitting diode
ICT: intramolecular charge transfer
DIPEA: diisopropylethylamine
NMR: nuclear magnetic resonance
HRMS: high-resolution mass spectrometry
TGA: thermogravimetric analysis
DSC: differential scanning calorimetry
CV: cyclic voltammetry
IP: ionization potential
EA: electron affinity
PLQY: photoluminescence quantum yield
SOC: spin–orbit coupling
ISC: intersystem crossing rate
RISC: reverse ISC
mCP: *N*,*N*′-dicarbazolyl-3,5-benzene
DFT: density functional theory
TD-DFT: time-dependent DFT
PCM: polarizable continuum model
TZP: triple-ζ polarized
COSMO: conductor-like screening model
HOMO: highest occupied molecular orbital
LUMO: lowest unoccupied molecular orbital
EL: electroluminescence
