# Supplementary material for: Sterically Tuned Ortho-Phenylene-Linked Donor–Acceptor Benzothiazole-Based Boron Difluoride Complexes as Thermally-Activated Delayed Fluorescence Emitters for Organic Light-Emitting Diodes
Source: ACS Appl Mater Interfaces. 2024 Oct 22;16(44):60633–47. doi: 10.1021/acsami.4c12662 (PMC11551907; doi:10.1021/acsami.4c12662)
Supplement: Supplementary file 1 — am4c12662_si_001.pdf [file am4c12662_si_001.pdf]

Supporting Information

Sterically Tuned *Ortho*-Phenylene Linked Donor-Acceptor Benzothiazole-Based Boron Difluoride Complexes as Thermally-Activated Delayed Fluorescent Emitters for Organic Light-Emitting Diodes

Stepan Kutsiy,[a,b] Dmytro Volyniuk,[c] Smruti Ranjan Sahoo,[d,e] Magdalena Ceborska,[f] Agnieszka Wisniewska,[g] Pavlo Stakhira,[b] Juozas Vidas Grazulevicius,[c,]* Glib V. Baryshnikov,[d,]* Mykhaylo A. Potopnyk[a,h,]*

[a] Institute of Organic Chemistry, Polish Academy of Sciences, Kasprzaka 44/52, 01-224, Warsaw, Poland, Email: mykhaylo.potopnyk@icho.edu.pl

[b] Department of Electronic Devices, Lviv Polytechnic National University, 1 Sviatoho Yura sq., Lviv 79013, Ukraine

[c] Department of Polymer Chemistry and Technology, Kaunas University of Technology, Barsausko 59, LT-51423 Kaunas, Lithuania, Email: juozas.grazulevicius@ktu.lt

[d] Laboratory of Organic Electronics, Department of Science and Technology, Linköping University, Norrköping SE-60174, Sweden, Email: glib.baryshnikov@liu.se

[e] Department of Physics and Astronomy, Uppsala University Box 516, SE-75120 Uppsala, Sweden

[f] Faculty of Mathematics and Natural Sciences, Cardinal Stefan Wyszynski University in Warsaw, K. Woycickiego 1/3, 01-938, Warsaw, Poland

[g] Institute of Physical Chemistry, Polish Academy of Sciences, Kasprzaka 44/52, 01-224, Warsaw, Poland

[h] Institute of Organic Chemistry, National Academy of Sciences of Ukraine, Akademika Kuharya Str. 5, 02000, Kyiv, Ukraine, Email: potopnyk@gmail.com

Contents

1. Single Crystal X-ray Diffraction S3
2. Thermal Analysis S8
3. Electrochemical Properties and Ultraviolet Photoelectron Spectroscopy S10
4. Charge-Transporting Properties S12
5. Photophysical Properties S14
6. Electroluminescent Performance S22
7. Transient Electroluminescence Measurements S22
8. Copies of NMR Spectra S24
9. References S35

1. Single Crystal X-ray Diffraction

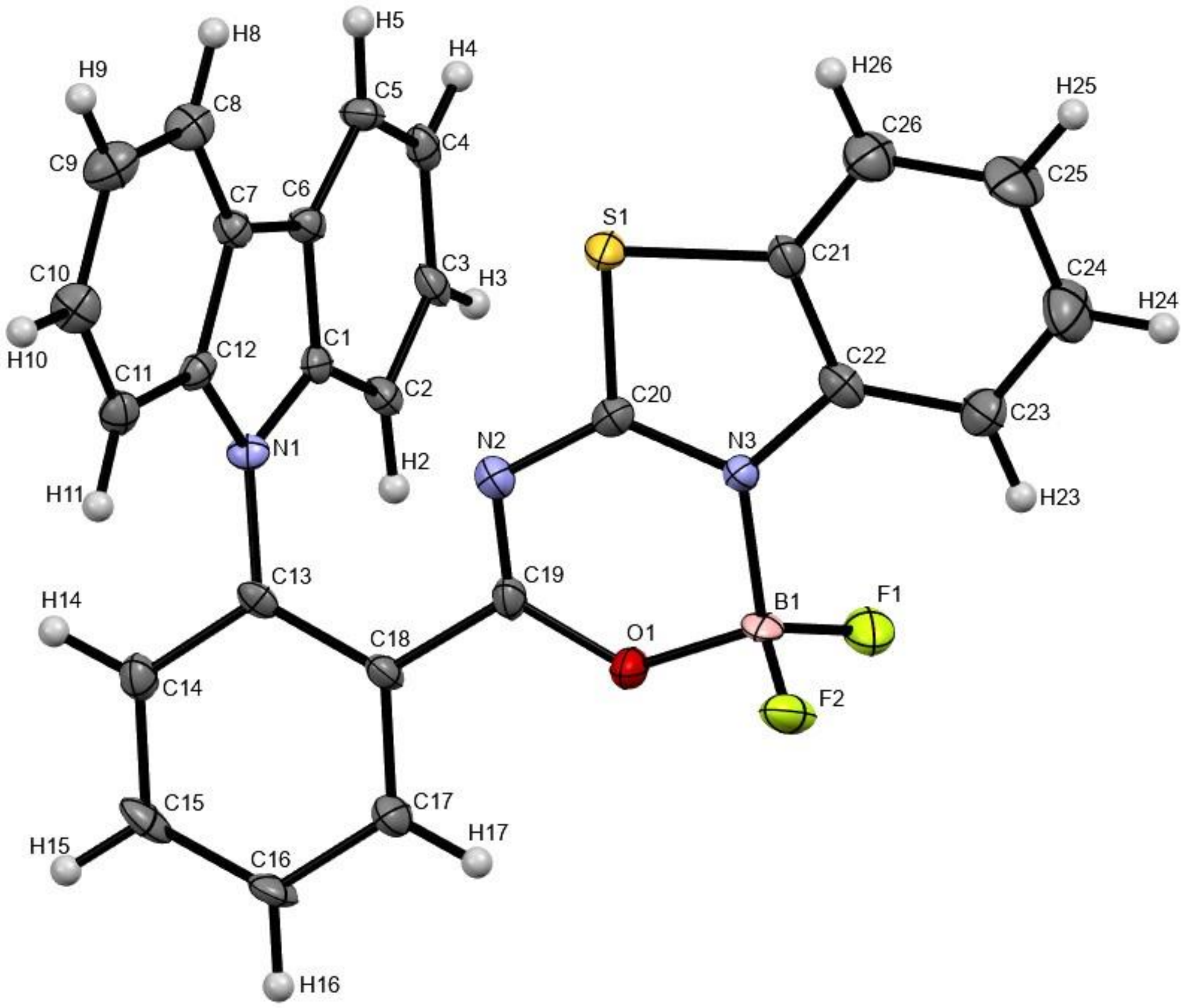

Figure S1. ORTEP diagram of compound **1**. The ellipsoid contour of probability level is 50%.

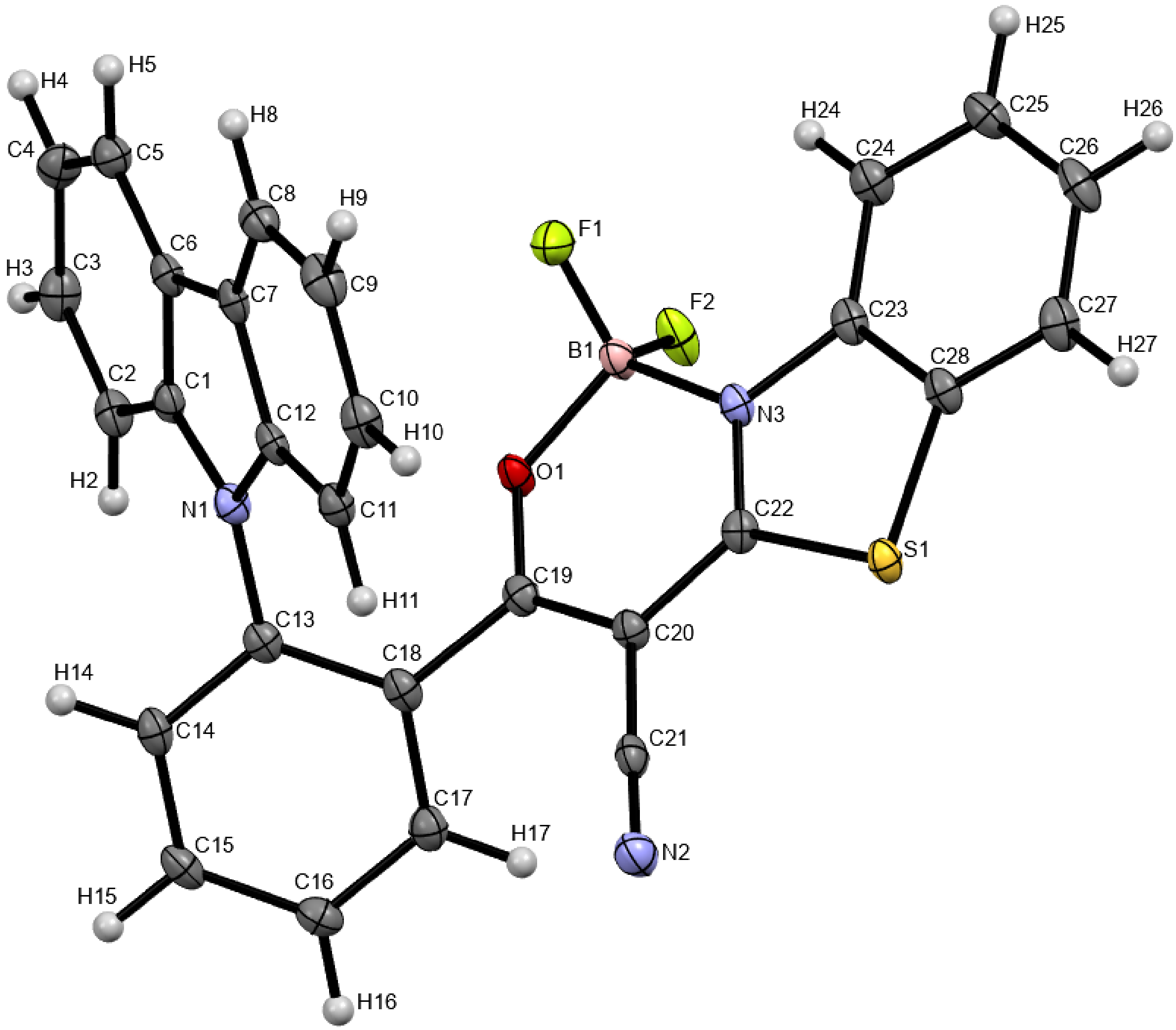

Figure S2. ORTEP diagram of compound **2**. The ellipsoid contour of probability level is 50%.

Table S1. Crystal data of boron difluoride complexes **1** and **2**.

Compound	**1**	**2**	
Empirical formula	$C_{26}H_{16}BF_2N_3OS$	$C_{28}H_{16}BF_2N_3OS$	
Moiety formula	$C_{26}H_{16}BF_2N_3OS$	$C_{28}H_{16}BF_2N_3OS$	
Formula weight	467.29	491.31	
CCDC No.	2329986	2293522	
Wavelength	1.54184	1.54184	
Crystal system	tetragonal	monoclinic	
Space group	P-42_1c	$P2_1/c$	
Unit cell dimensions	a = 23.5241(2) Å<br>b = 23.5241(2) Å<br>c = 8.0969(1) Å	a = 12.9208(3) Å<br>b = 8.5205(2) Å<br>c = 20.3092(5) Å	β = 95.040(2)°
Volume	4480.69(9) Å^3	2227.23(9) Å^3	
Z	8	4	
Density Calc.	1.385 g/cm^3	1.465 g/cm^3	
Absorption coefficient	1.639 mm^{-1}	1.681 mm^{-1}	
F(000)	1920	1008	
Crystal	yellow needle	Orange needle	
Crystal size	0.22 × 0.04 × 0.03	0.22 × 0.04 × 0.03	
Index ranges	-26 ≤ h ≤ 20, -27 ≤ k ≤ 28, -3 ≤ l ≤ 9	-14 ≤ h ≤ 15, -10 ≤ k ≤ 5, -24 ≤ l ≤ 24	
Reflections collected (all / independent)	8138 / 3796 [R_{int} = 0.02]	8126 / 4157 [R_{int} = 0.029]	
Absorption correction	Multi-scan	Multi-scan	
Refinement method	Full-matrix least-squares on F^2	Full-matrix least-squares on F^2	
Restraints / parameters	0 / 307	0 / 325	
Goodness-of-fit on F^2	1.054	1.028	
Final R indices [$F^2 > 2\sigma(F^2)$]	R = 0.029, wR = 0.076	R = 0.039, wR = 0.104	
R indices (all data)	R = 0.031, wR = 0.078	R = 0.043, wR = 0.109	

Table S2. Single crystal analysis of intermolecular interactions for compound **1**.

Entry	D−H···A	D−H (Å)	*d*(D−A) [Å]	*d*(H…A) [Å]	<(D-H…A) [°]
1.	C5—H5···Cg(a) [(a)]	0.95	3.875	2.764	161
2.	C8—H8···Cg(b) [(a)]	0.95	3.849	2.985	162
3.	C15—H15···Cg(c)[(b)]	0.95	3.741	2.932	144
4.	C3—H3···F1 [(c)]	0.95	3.332	2.631	130
5.	C3—H3···O1 [(c)]	0.95	3.658	2.714	171
6.	C16—H16···F2 [(b)]	0.95	3.403	2.634	138
7.	C26—H26···F1 [(d)]	0.95	3.307	2.572	134

Symmetry operations: [(a)] ½+x, -1/2+y, -1/2+z, [(b)] x, y, 1+z [(c)] 1+x, 1-y, 3-z [(d)]1+x, 1-y, 2-z. For CH−π interactions the distances were measured between specific atoms and centroids measured for phenyl ring (C1-C6 for distance **a,** Cg(a)); bicyclic part of carbazole ring (for distance **b**, Cg(b) and for phenyl ring (C7-C12 for distance **c,** Cg(c)).

Table S3. Single crystal analysis of intermolecular interactions for compound **2**.

Entry	D−H···A	D−H (Å)	*d*(D−A) [Å]	*d*(H…A) [Å]	<(D-H…A) [°]
1.	C5—H5···N2 [(a)]	0.95	3.500	2.678	145
2.	C8—H8···N2 [(a)]	0.95	3.511	2.686	146
3.	C15—H15···N2 [(b)]	0.95	3.333	2.686	126
4.	C25—H25···F1 [(c)]	0.95	3.292	2.569	133
5.	C26—H26···F2 [(d)]	0.95	3.170	2.549	123
6.	C27—H27···O1 [(d)]	0.95	3.317	2.683	125

Symmetry operations: [(a)] x, 1/2-y, ½+z [(b)] 1-x, -y, -z [(c)] –x, ½+y, ½-z [(d)] x, 1+y, z

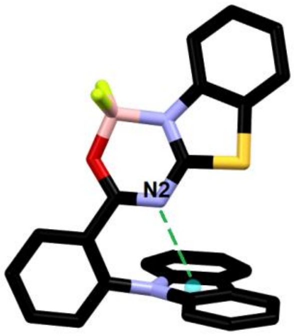

Figure S3. N···π interaction stabilizing bent conformation of compound **1**.

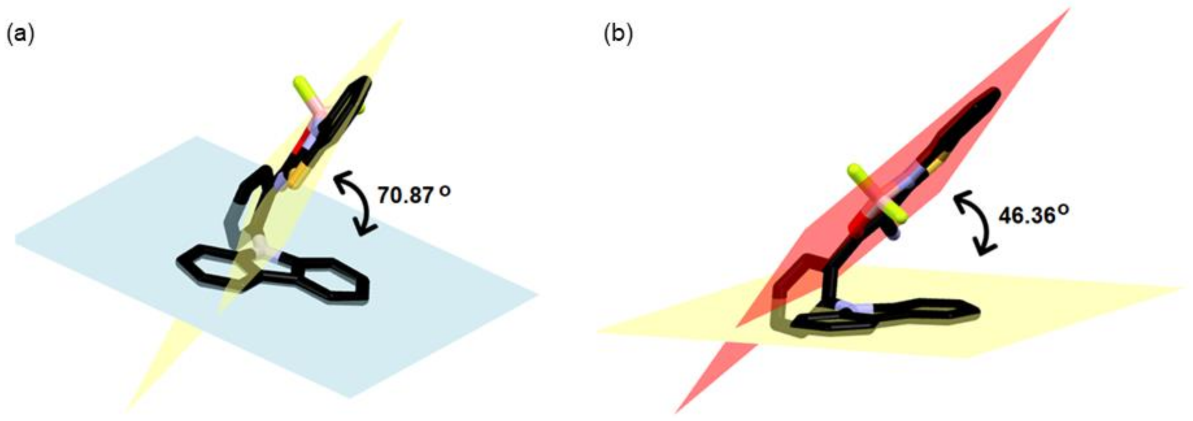

Figure S4. (a) Crystal structure of compound **1**, shown with the angle formed between planes (comprising carbazole moiety is blue and comprising BF_2 complex is yellow); (b) Crystal structure of compound **2**, shown with the angle formed between planes (comprising carbazole moiety is yellow and comprising BF_2 complex is red). Hydrogen atoms are omitted for clarity.

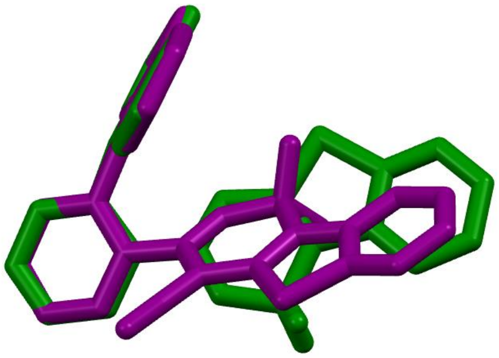

Figure S5. Overlay of structures of compounds **1** (green) and **2** (purple).

2. Thermal Analysis

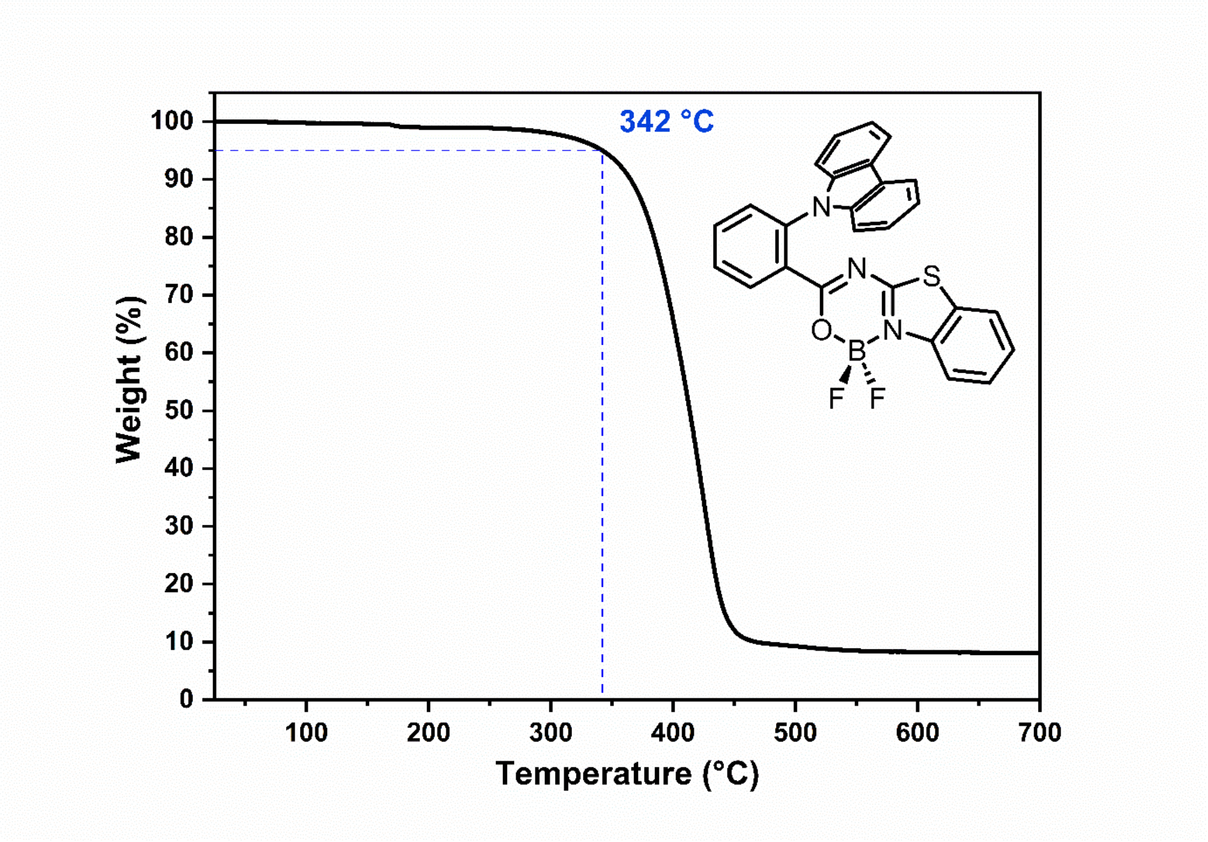

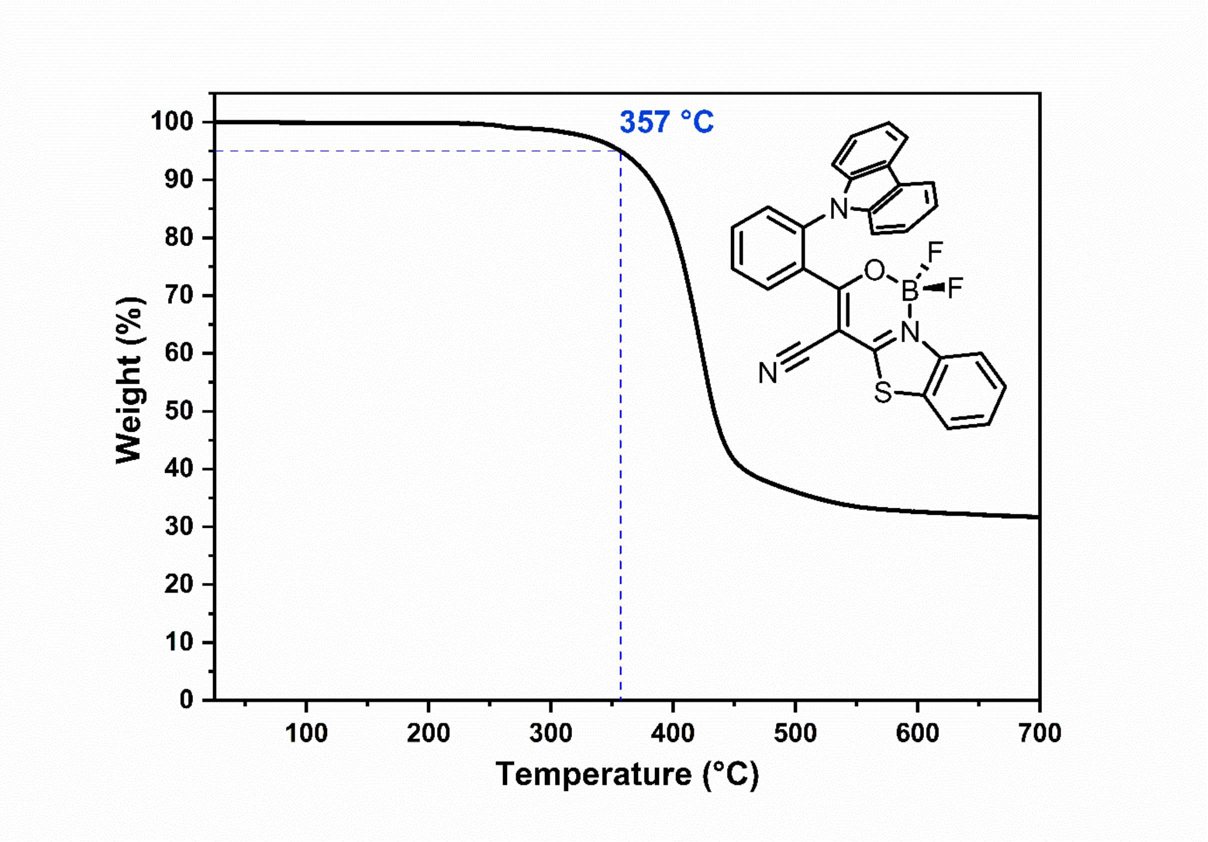

Figure S6. TGA curves of boron difluoride complexes **1** (top) and **2** (bottom).

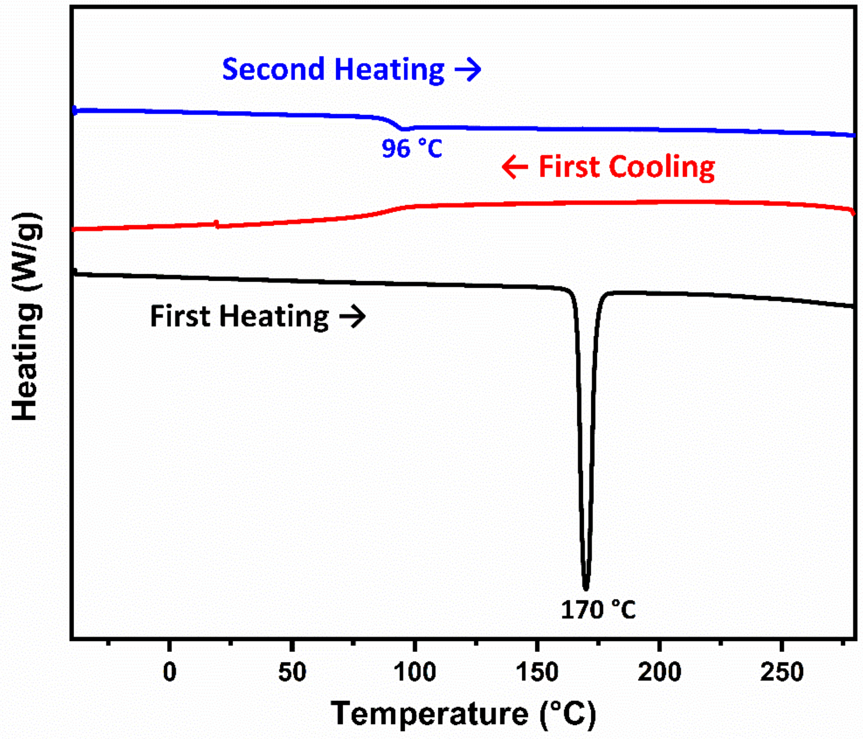

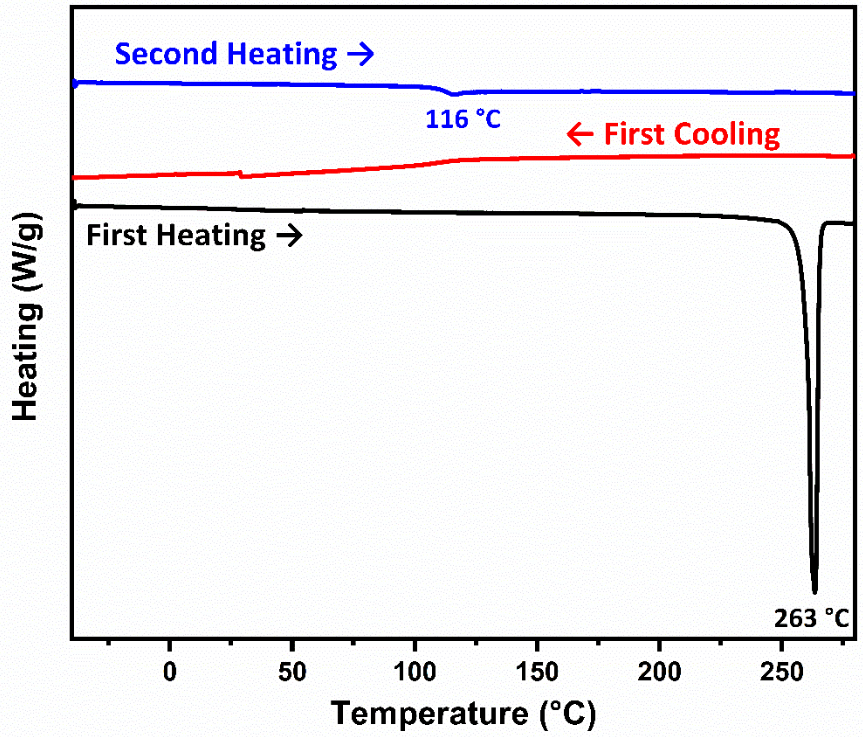

Figure S7. DSC thermograms of boron difluoride complexes **1** (top) and **2** (bottom).

3. Electrochemical Properties and Ultraviolet Photoelectron Spectroscopy

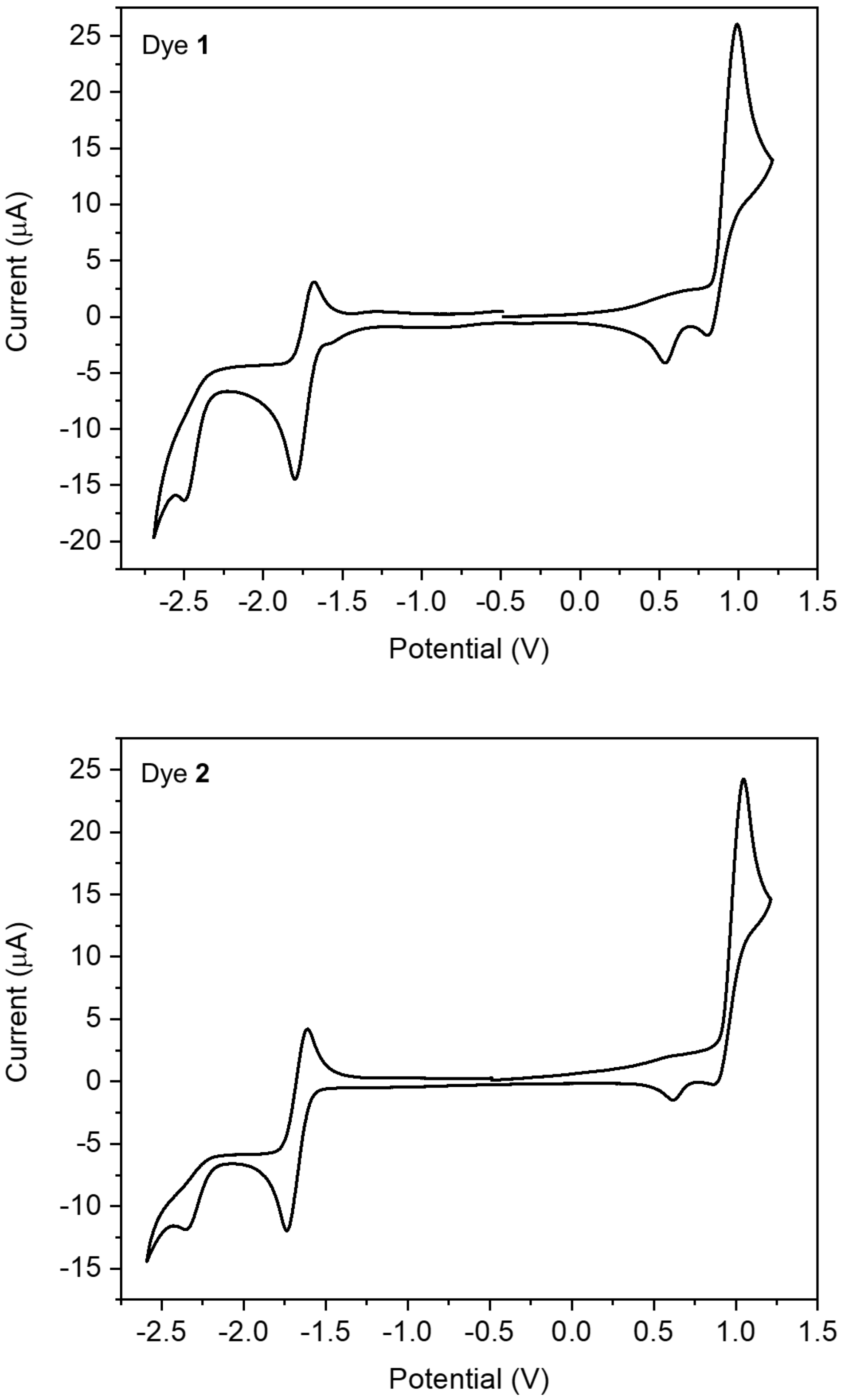

Figure S8. Cyclic voltammogram of compound **1** (top) and **2** (bottom).

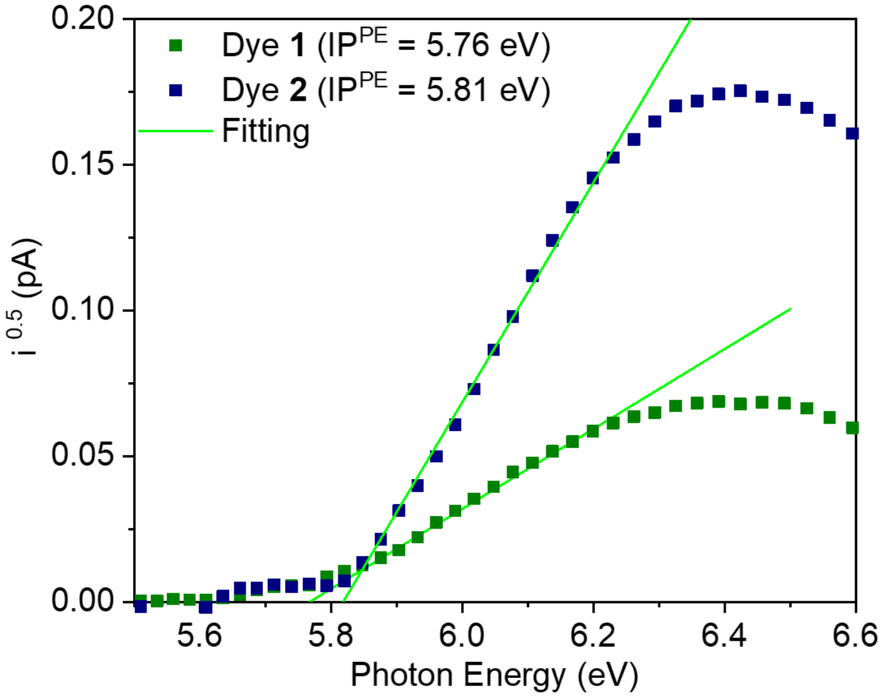

Figure S9. Electron photoemission spectra of the studied compounds.

Table S4. Oxidation and Reduction Potentials, Ionization Potentials, and Electron Affinities of Compounds **l** and **2**.

Dye	E_{ox} (V) [a]	E_{red} (V) [b]	IP (eV) [c]	EA (eV) [d]	E_g (eV) [e]	IP^{PE} (eV) [f]
**1**	0.90	-1.74	5.70	3.06	2.64	5.76
**2**	0.95	-1.67	5.75	3.13	2.62	5.81

[a] E_{ox} – oxidation potential. [b] E_{red} – reduction potential. [c] IP – the ionization potential obtained from cyclic voltammetry, IP = E_{ox} + 4.8. [e] EA – the electron affinity, EA = E_{red} + 4.8. [e] Energy gap, E_g = IP – EA. [f] IP^{PE} – the ionization potential obtained from election photoemission spectra.

4. Charge-Transporting Properties

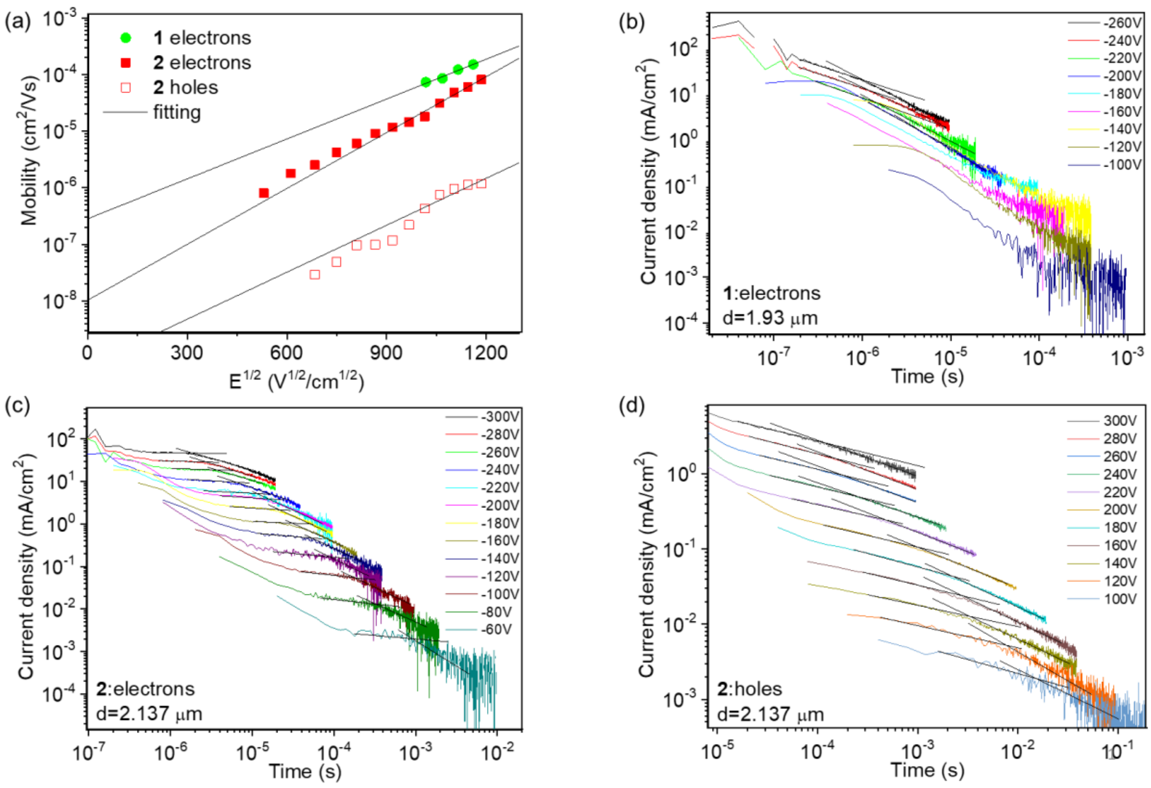

Figure S10. Poole-Frenkel type plots of charge carrier mobility versus electric field for the layers of compounds **1** and **2** (a). Electron TOF signals for compounds **1** (b) and **2** (c). Hole TOF signals for compound **2** (d).

Table S5. Hole and electron mobility parameters at room temperature, obtained for vacuum-deposited layers of dyes **1** and **2**.

	Holes			Electrons		
Dye	μ_h [a] ($cm^2\ V^{-1}\ s^{-1}$)	μ_0 [b] ($cm^2\ V^{-1}\ s^{-1}$)	β [c] ($(cm\ V^{-1})^{1/2}$)	μ_e [a] ($cm^2\ V^{-1}\ s^{-1}$)	μ_0 ($cm^2\ V^{-1}\ s^{-1}$)	β ($(cm\ V^{-1})^{1/2}$)
**1**	–	–	–	1.5×10^{-4}	2.8×10^{-7}	5.4×10^{-3}
**2**	1×10^{-6}	7.1×10^{-10}	6.37×10^{-3}	0.7×10^{-4}	0.1×10^{-7}	7.58×10^{-3}

[a] Taken from the TOF measurements (**Figure S10a**), hole (μ_h) and electron (μ_e) mobilities at electric field (E) of 1.35×10^6 V/cm; [b] Mobilities at zero electric field (μ_0) and [c] field dependence parameter (β) of a Poole-Frenkel type mobility obtained by fitting of experimental data using formula $\mu = \mu_0 \times \exp(\beta \times E^{1/2})$.

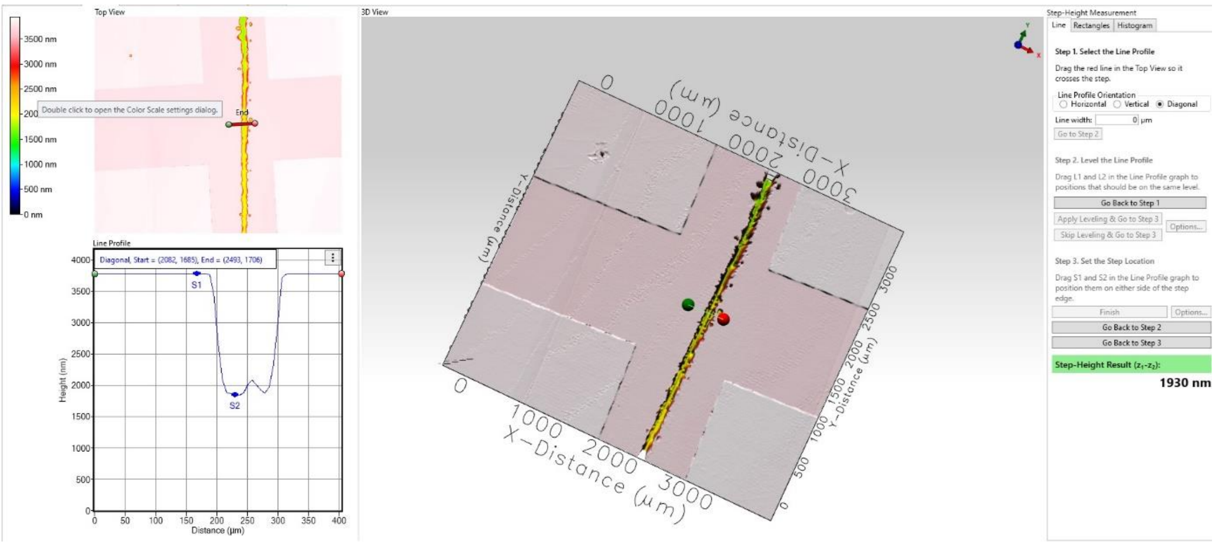

Figure S11. Thickness measurements of TOF samples of compound **1**.

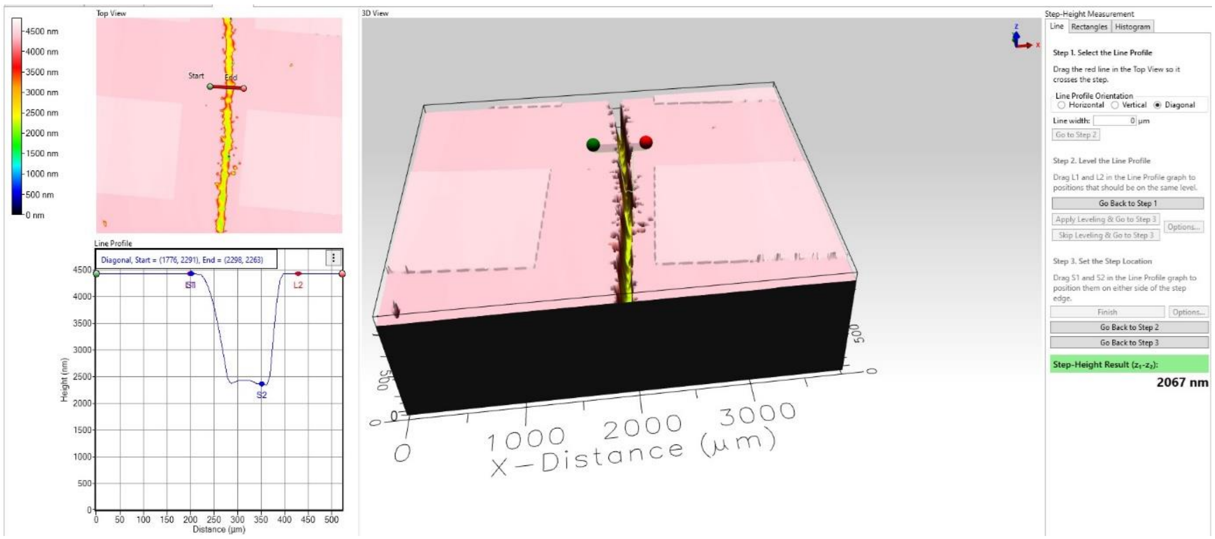

Figure S12. Thickness measurements of TOF samples of compound **2**.

5. Photophysical Properties

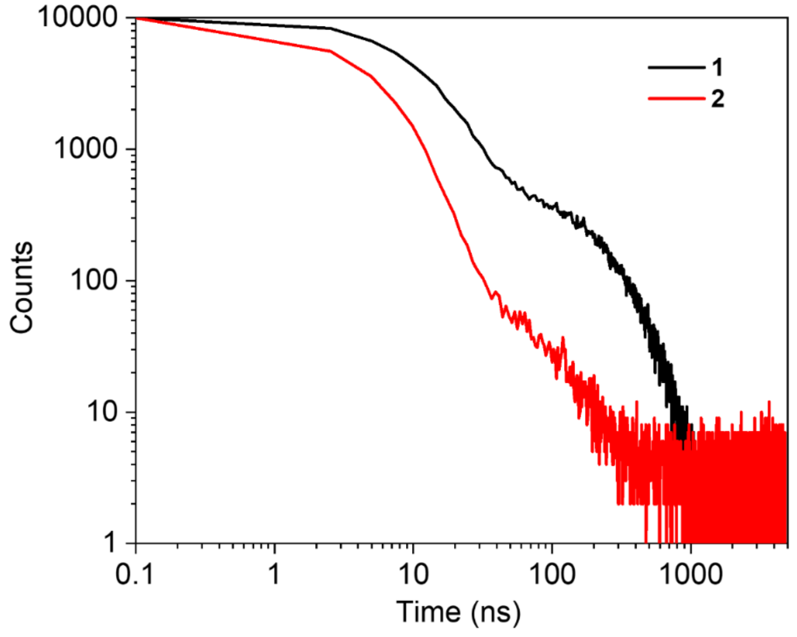

Figure S13. Photoluminescence decay curves of compounds **1** and **2** in toluene.

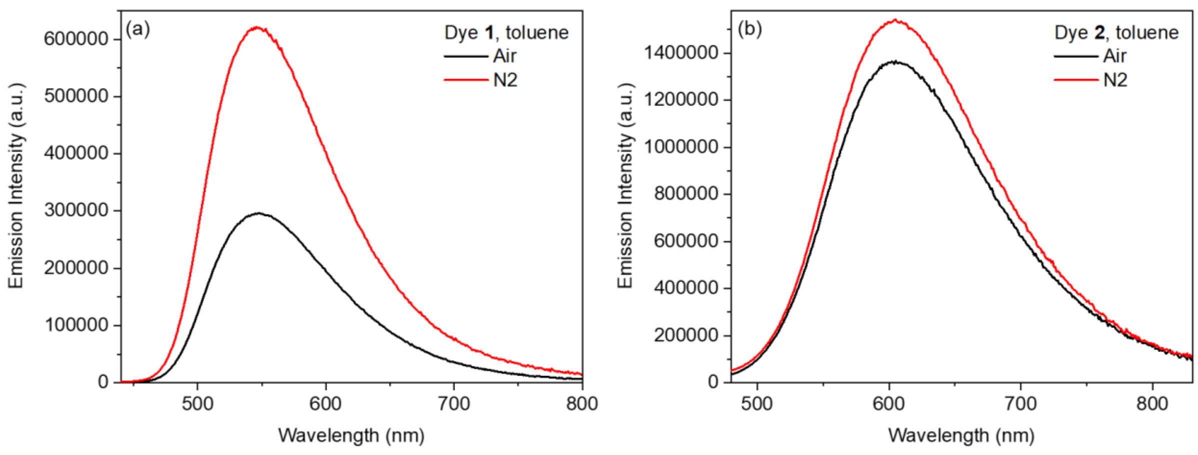

Figure S14. Photoluminescence spectra of the toluene solution of compounds **1** (a) and **2** (b) at air N_2 conditions.

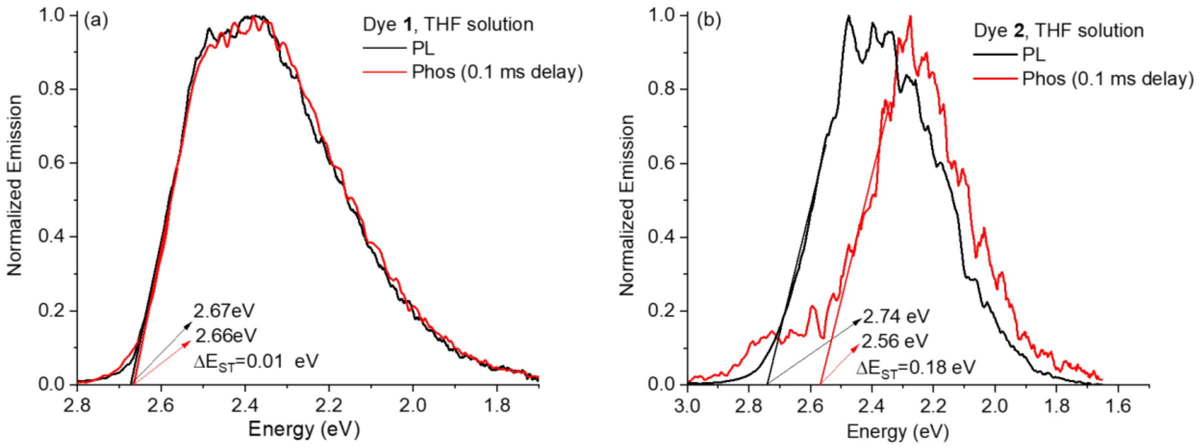

Figure S15. Photoluminescence and phosphorescence (with 0.1 ms delay) spectra of dilute solutions of dyes **1** (a) and **2** (b) in THF recorded at 77 K.

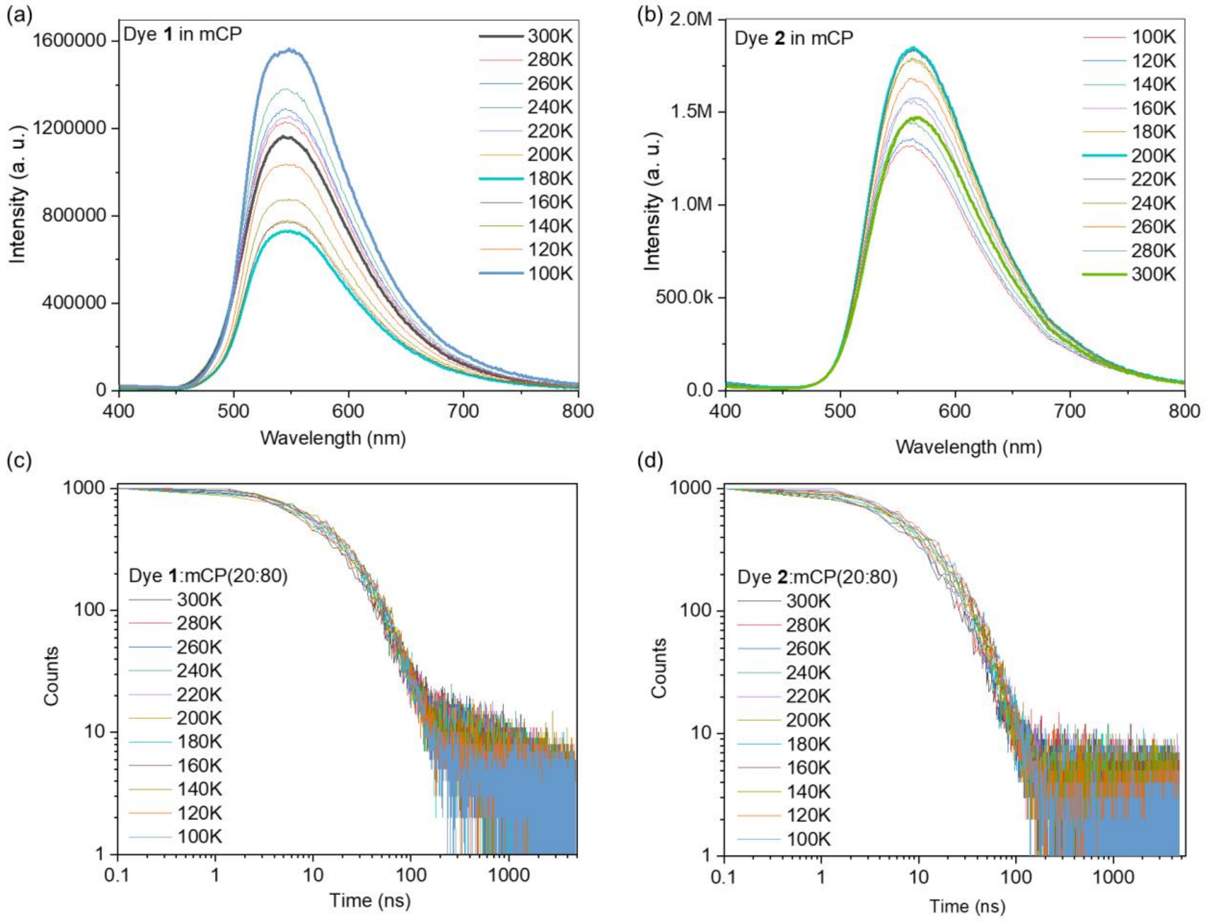

Figure S16. PL spectra (a, b) and PL decay curves (c, d) of mCP based films of dyes **1** and **2** (20 wt.%) at different temperatures.

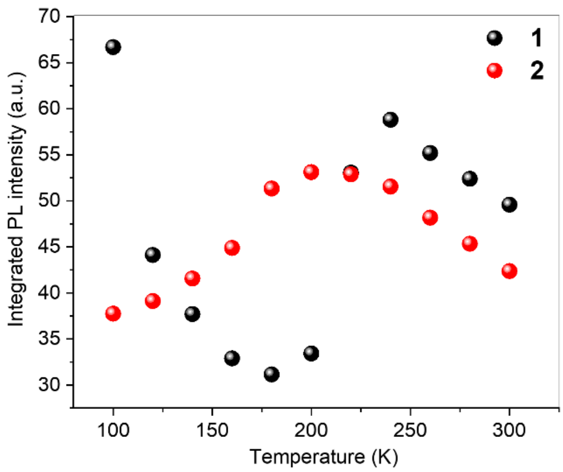

Figure S17. Integrated photoluminescent intensities of mCP based films of dyes **1** and **2** (20 wt.%) at different temperatures, showing that PL intensity decreases with the increase of temperature due to the decrease in intensity of phosphorescence; then, PL intensity increases with the further increase of temperature due to the increase in intensity of TADF until nonradiative relaxation processes are dominated.

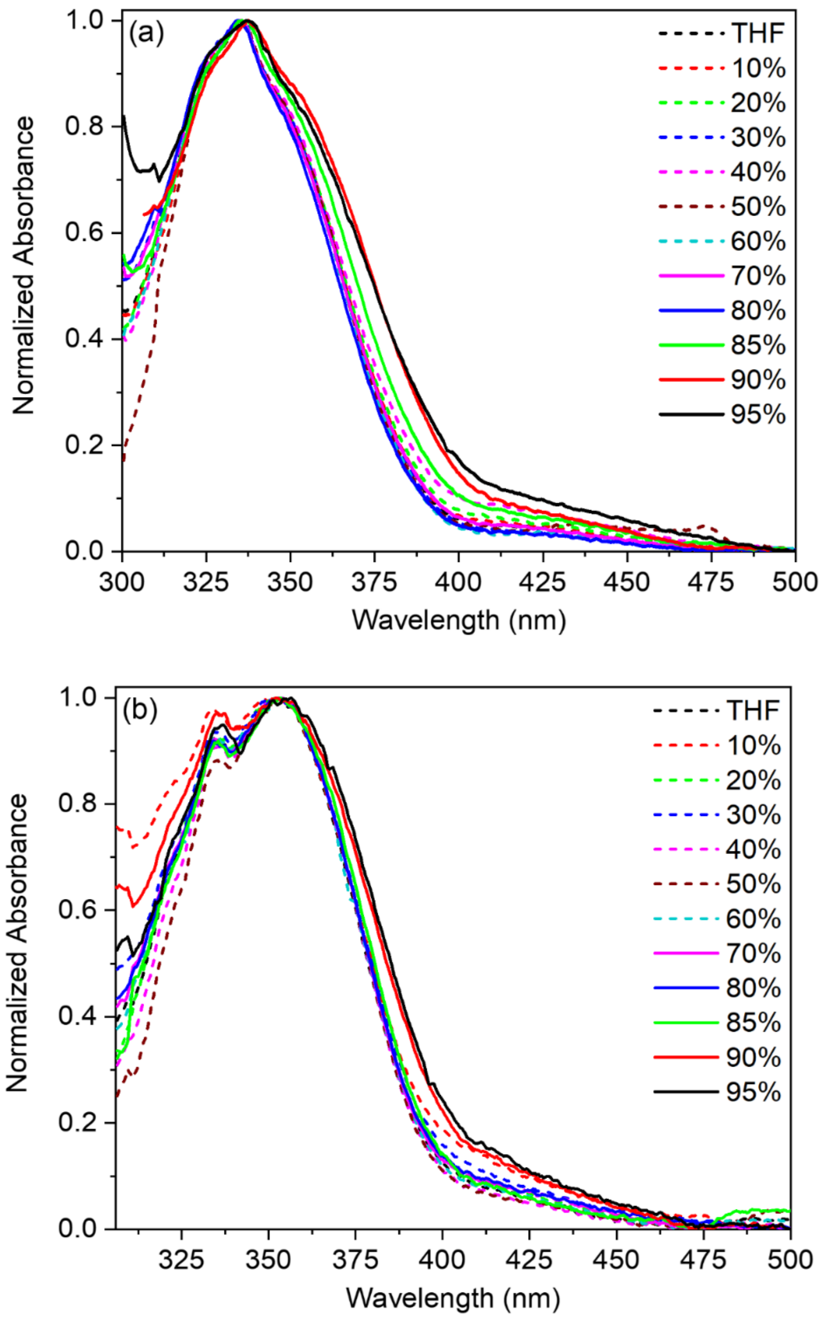

Figure S18. Absorption spectra of the dispersions of dyes **1** (a) and **2** (b) in THF/water mixtures of varying water content.

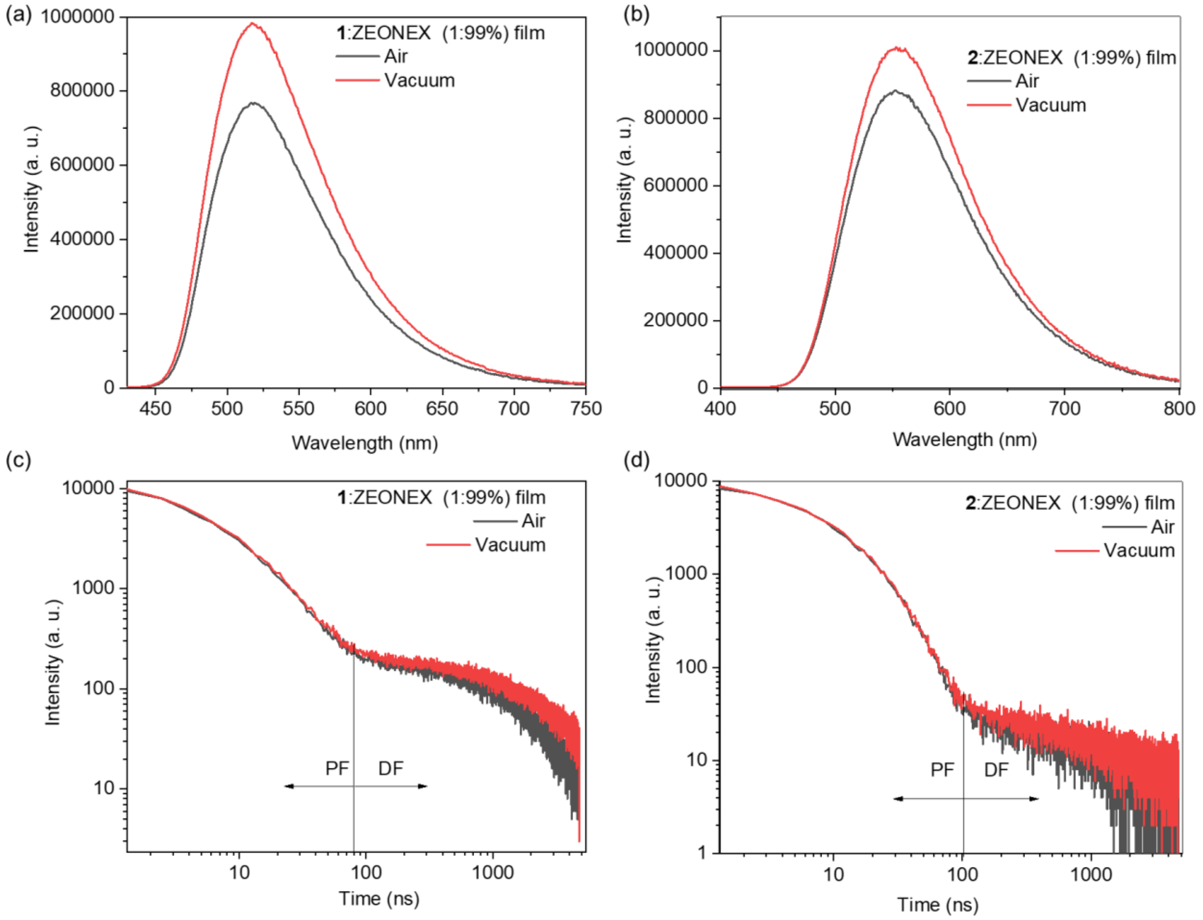

Figure S19. Photoluminescence spectra of dye-doped ZEONEX films (1% of boron dye and 99% of ZEONEX) of compounds **1** (a) and **2** (b). Photoluminescence decay curves of the dye-doped ZEONEX films of compounds **1** (c) and **2** (d).

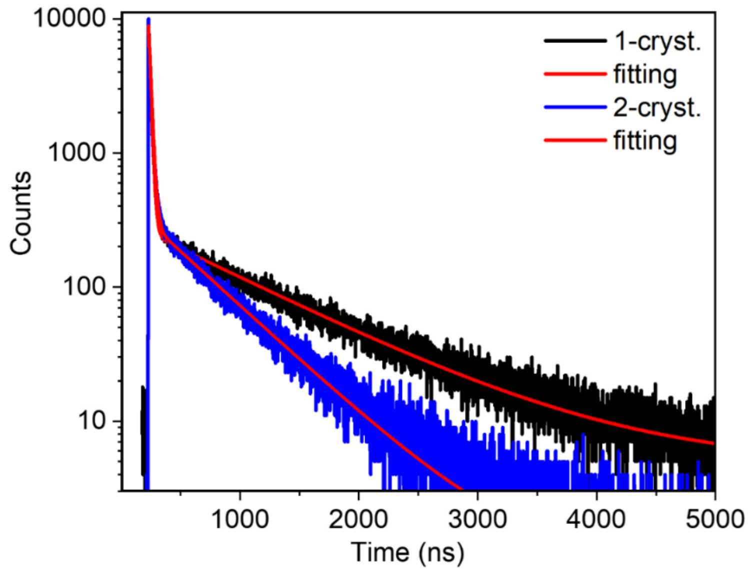

Figure S20. Photoluminescence decay curves of crystals of compounds **1** and **2**.

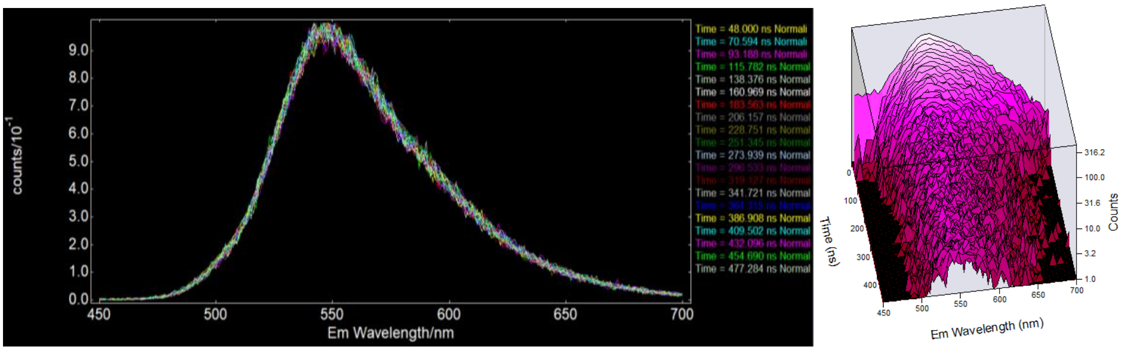

(a)

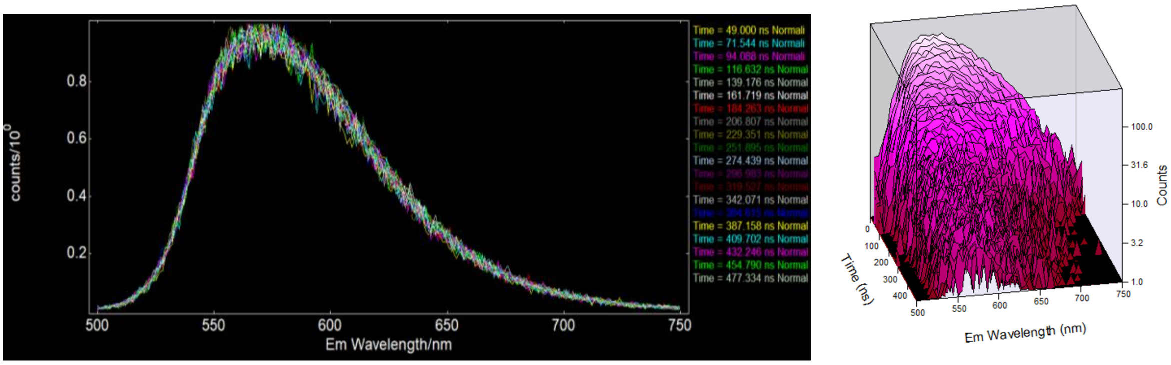

(b)

Figure S21. Time-resolved photoluminescent spectra of the crystals of compounds **1** (a) and **2** (b) plotted in 2D (left) and 3D (right) scales.

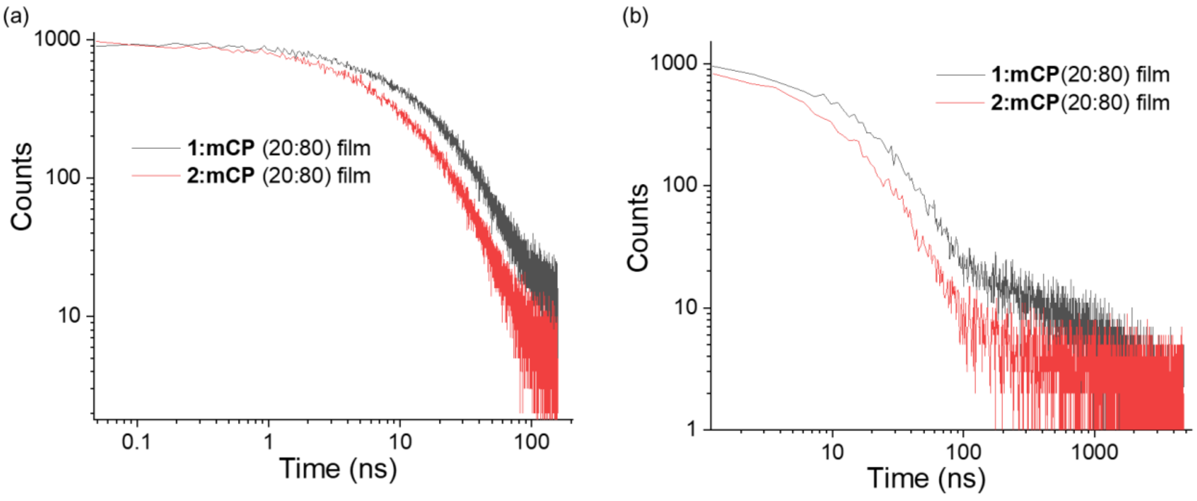

Figure S22. Photoluminescence decay curves of dye-doped mCP films (20% of boron dye and 80% of mCP) of compounds **1** and **2** recorded at nanosecond (a) and microsecond (b) ranges.

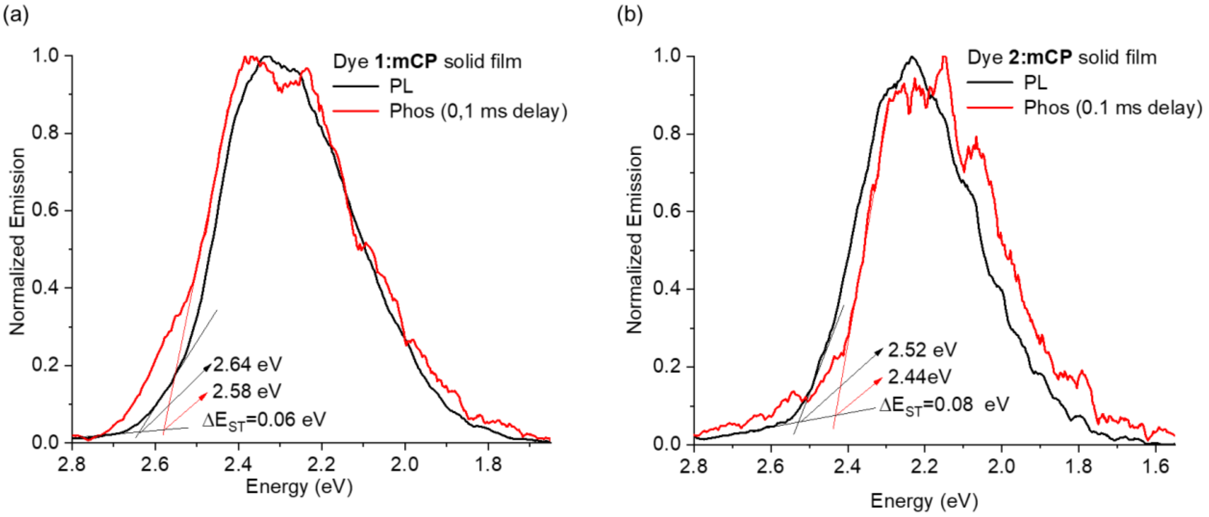

Figure S23. Photoluminescence (black line) and phosphorescence (red line) (with 0.1 ms delay) spectra of dye-doped mCP films (20% of boron dye and 80% of mCP) of compounds **1** (a) and **2** (b) recorded at 77 K.

The rate constants of the dyes were determined by using the following reported equations:[1]

$$k_{\mathrm{PF}} = \frac{\Phi_{\mathrm{PF}}}{\tau_{\mathrm{PF}}} \quad (1);$$

$$\mathrm{PLQY} = \Phi_{\mathrm{PF}} + \Phi_{\mathrm{DF}} = \frac{k_{\mathrm{PF}}}{k_{\mathrm{PF}} + k_{\mathrm{IC}}} \quad (2);$$

$$\Phi_{\mathrm{PF}} = \frac{k_{\mathrm{PF}}}{k_{\mathrm{PF}} + k_{\mathrm{IC}} + k_{\mathrm{ISC}}} \quad (3);$$

$$\Phi_{\mathrm{ISC}} = 1 - \Phi_{\mathrm{PF}} - \Phi_{\mathrm{IC}} = \frac{k_{\mathrm{ISC}}}{k_{\mathrm{PF}} + k_{\mathrm{IC}} + k_{\mathrm{ISC}}} \quad (4);$$

$$k_{\mathrm{DF}} = \frac{\Phi_{\mathrm{DF}}}{\Phi_{\mathrm{ISC}}\tau_{\mathrm{DF}}} \quad (5);$$

$$k_{\mathrm{RISC}} = \frac{k_{\mathrm{PF}}k_{\mathrm{DF}}}{k_{\mathrm{ISC}}}\frac{\Phi_{\mathrm{DF}}}{\Phi_{\mathrm{PF}}} \quad (6);$$

where k_{PF}, k_{DF}, k_{IC}, k_{ISC}, and k_{RISC} are the radiative rate constant for prompt fluorescence, delayed fluorescence, internal conversion, intersystem crossing, and reverse intersystem crossing, respectively; τ_{PT} is prompt lifetime; τ_{PT} is delayed lifetime; Φ_{PF}, Φ_{DF}, Φ_{IC}, and Φ_{ISC} are quantum yield of prompt fluorescence, delayed fluorescence, internal conversion, and intersystem crossing, respectively.

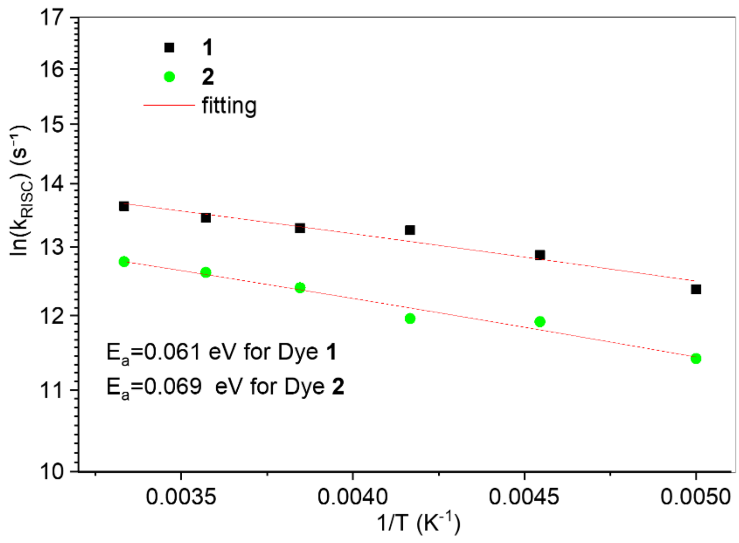

Figure S24. RISC temperature dependences of dyes **1** and **2** dispersed in Zeonex. The RISC activation energies E_A^{RISC} of dyes **1** and **2** were obtained by the linear fitting of the plots. The fitting was performed according to the Arrhenius dependence k=A×exp($-E_A^{RISC}/k_B$T), where k_B is Boltzmann constant and A is the frequency factor involving the spin–orbit coupling constant.[2]

6. Electroluminescent Performance

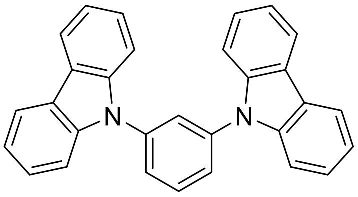

mCP

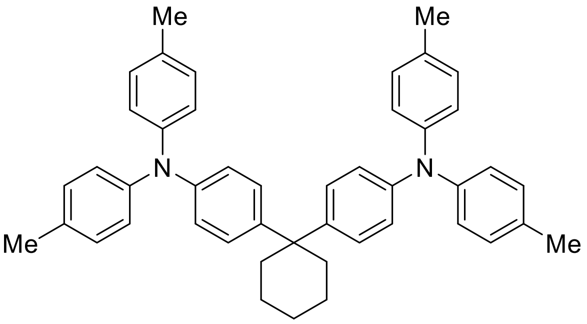

TAPC

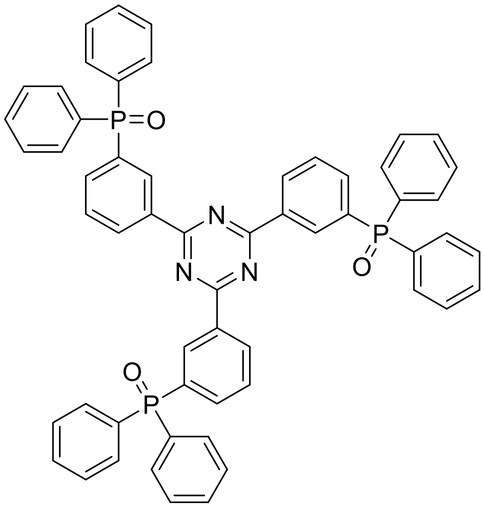

PO-T2T

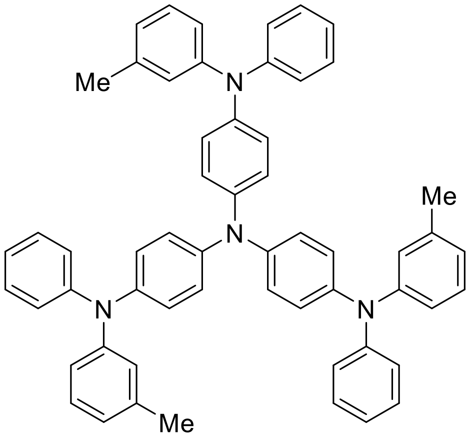

m-MTDATA

Figure S25. The chemical structures of semiconductor materials in OLEDs.

7. Transient Electroluminescence Measurements

The simplified devices based on the host-free light-emitting layers **1** and **2** were fabricated for the transient electroluminescence measurements. The structure of those devices was ITO/TAPC/**1** or **2** /TPBi/LiF:Al, where TPBi is 2,2',2''-(1,3,5-benzinetriyl)-tris(1-phenyl-1-H-benzimidazole) and LiF is lithium fluoride. The electroluminescence decay curves of those devices are well-described by two electroluminescence lifetime components (τ_1 and τ_2) (Table S6). The fitting error was in the required range of $1<\chi^2<1.3$. The trend of τ_1 and τ_2 of **1**- and **2**-based devices are the same as the trend of τ_{PF} and τ_{DF} of the studied emitters **1** and **2** (Table 2). For example, the values of long-lived PL (τ_{DF}) and EL (τ_2) time components of emitter **1** and **1**-based devices are higher than those of emitter **2** and **2**-based devices, respectively. A similar trend is observed for prompt PL and EL time components of the studied compounds. The values of EL lifetime components τ_1 and τ_2 of the devices are much longer than the values of PL lifetime components τ_{PF} and τ_{DF} because of the effects of charge-transporting properties of the studied devices.

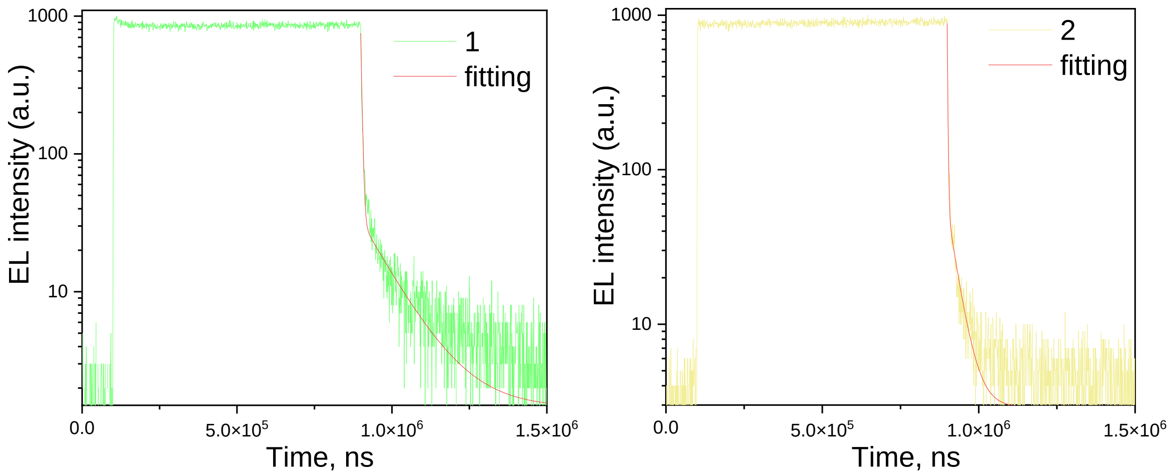

Figure S26. Electroluminescence decay curves of **1**- and **2**-based devices.

Table S6. Electroluminescence lifetimes of **1**- and **2**-based devices.

Device	τ_1 (μs)	A_1, %	τ_2 (μs)	A_2, %	χ^2
**1**-based	3.7	51	105.9	49	1.046
**2**-based	1.9	60	31.8	40	1.271

8. Copies of NMR Spectra

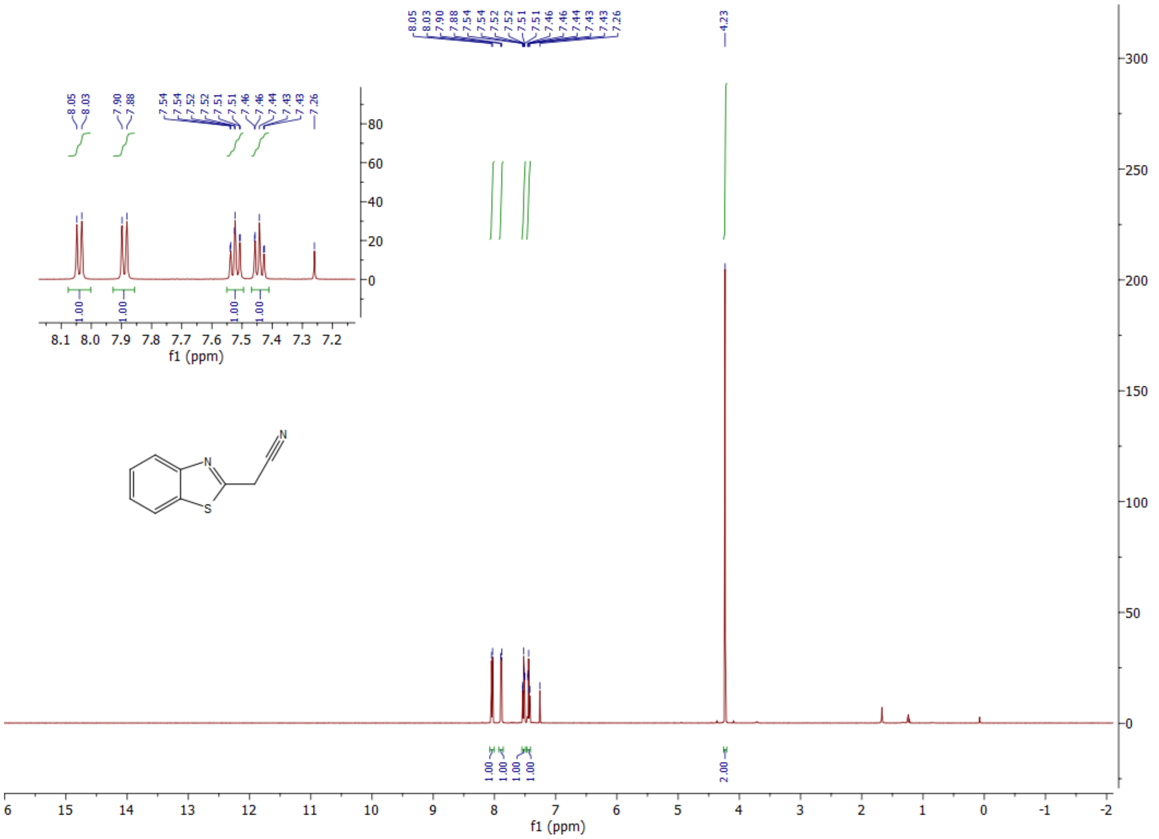

Figure S27. ^{1}H NMR (500 MHz, $CDCl_3$) spectrum of compound **4**.

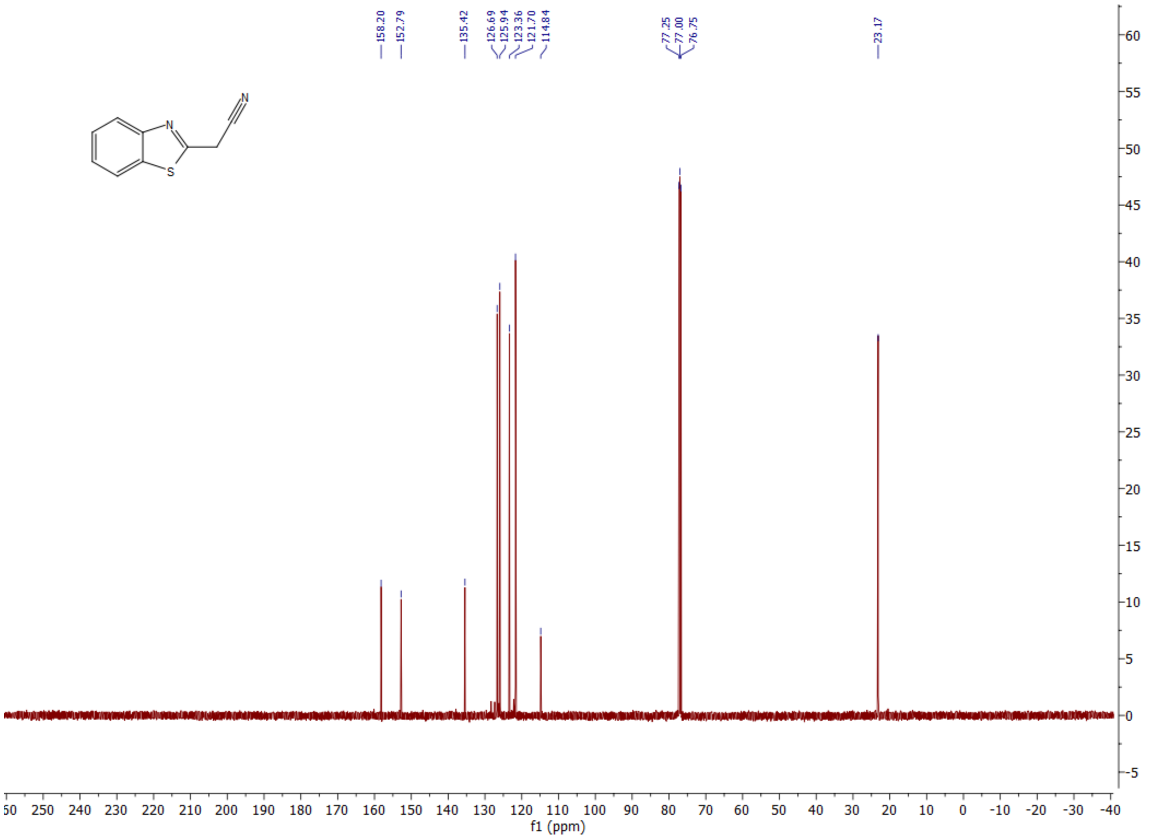

Figure S28. ^{13}C{H} NMR (125 MHz, $CDCl_3$) spectrum of compound **4**.

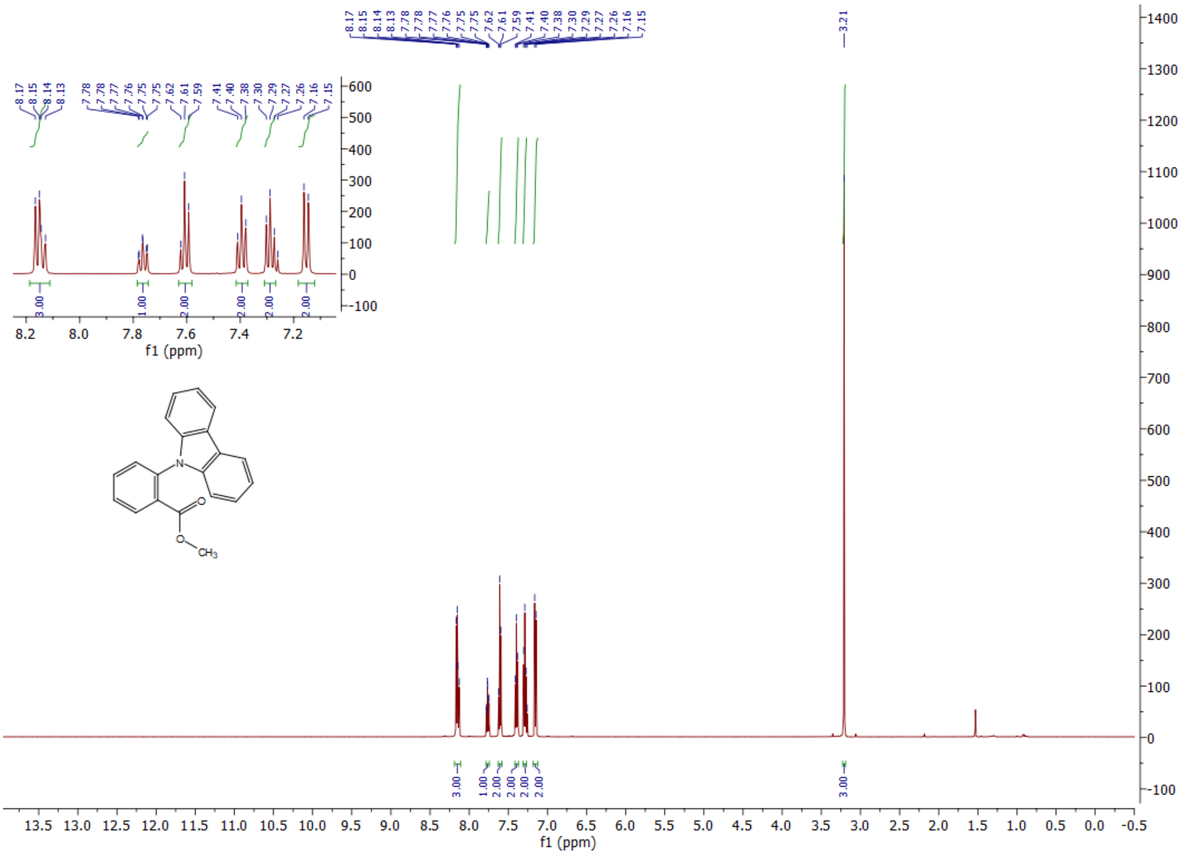

Figure S29. ^{1}H NMR (500 MHz, $CDCl_3$) spectrum of compound **7**.

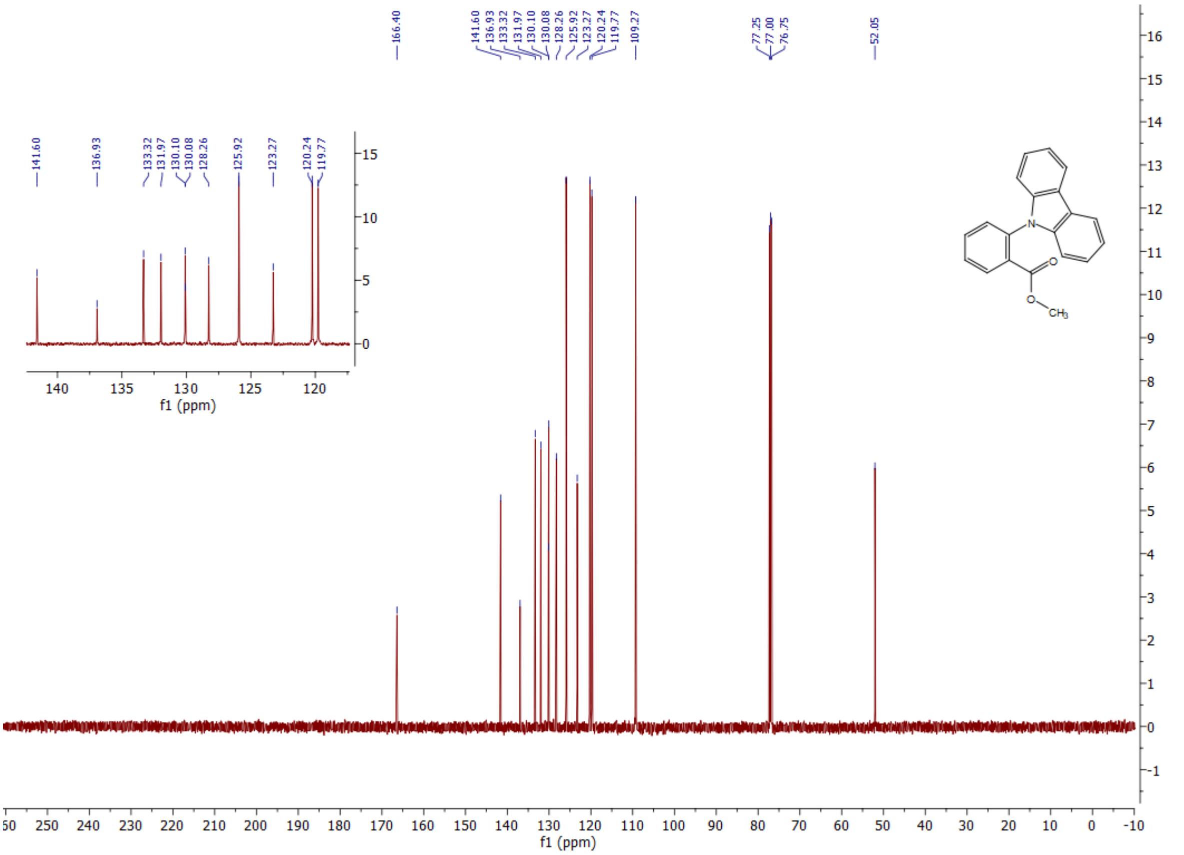

Figure S30. ^{13}C{H} NMR (125 MHz, $CDCl_3$) spectrum of compound **7**.

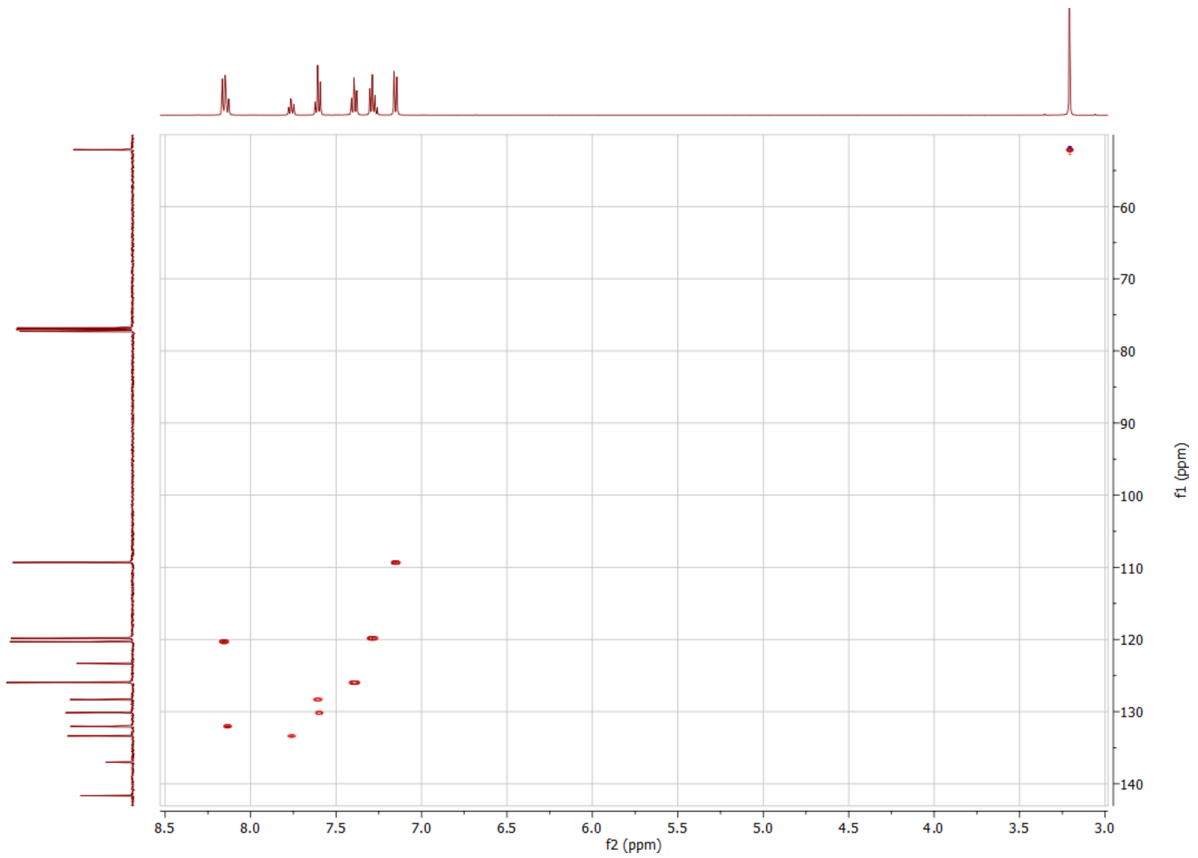

Figure S31. ^{1}H–^{13}C HSQC NMR (125 MHz, $CDCl_3$) spectrum of compound **7**.

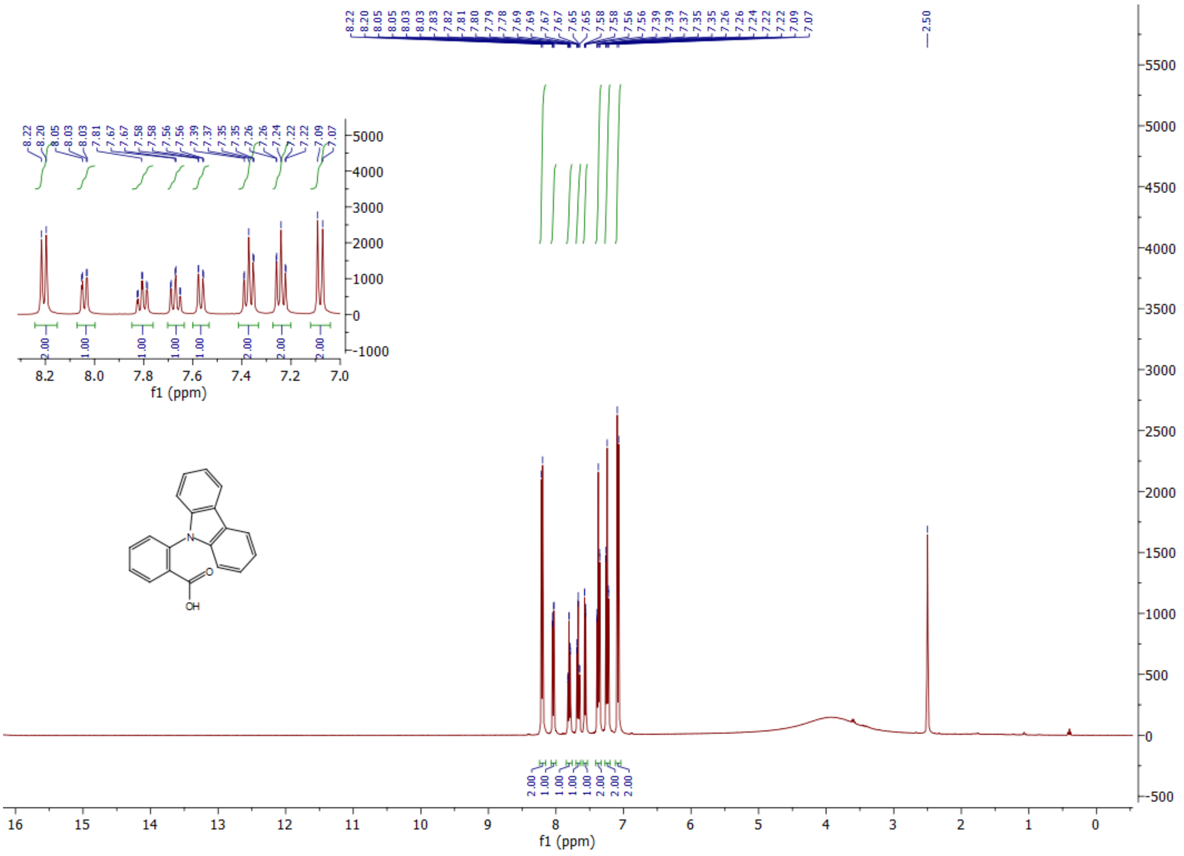

Figure S32. ^{1}H NMR (400 MHz, DMSO-d_6) spectrum of compound **8**.

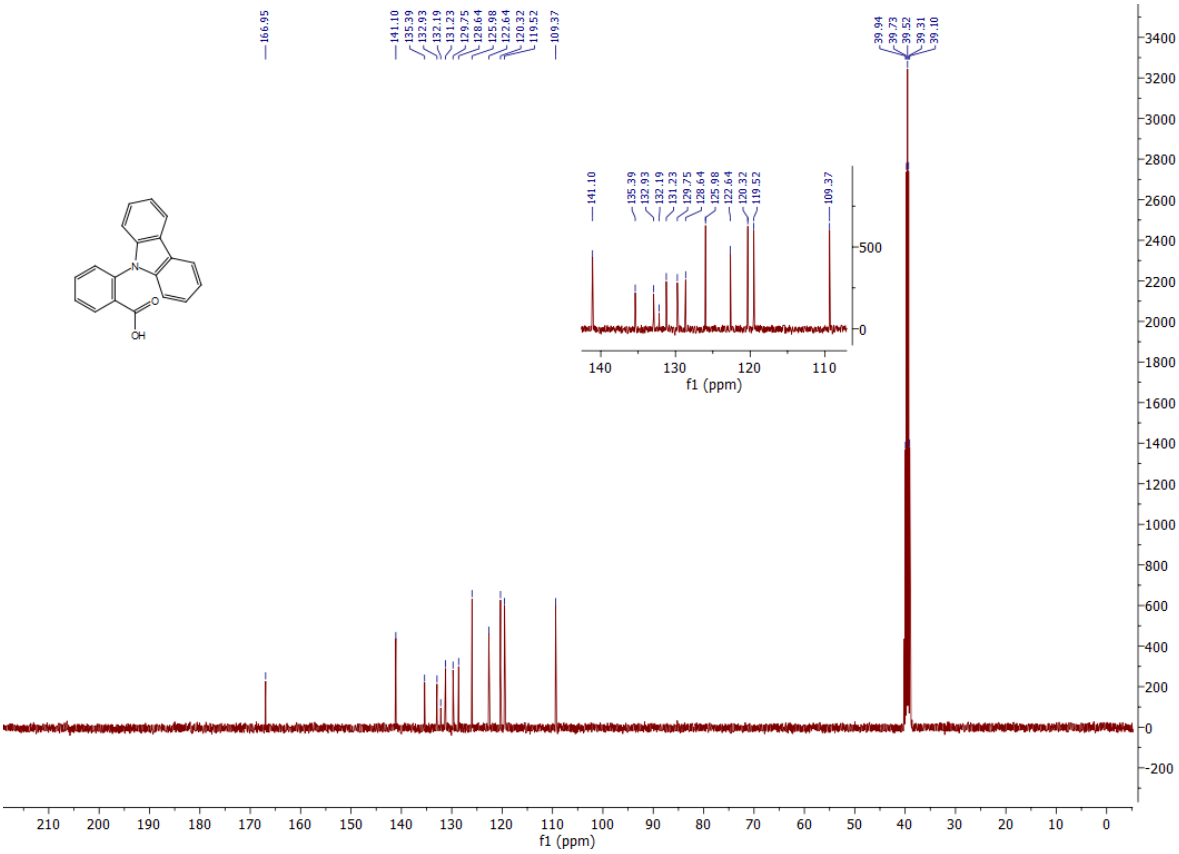

Figure S33. $^{13}C\{H\}$ NMR (100 MHz, DMSO-d_6) spectrum of compound **8**.

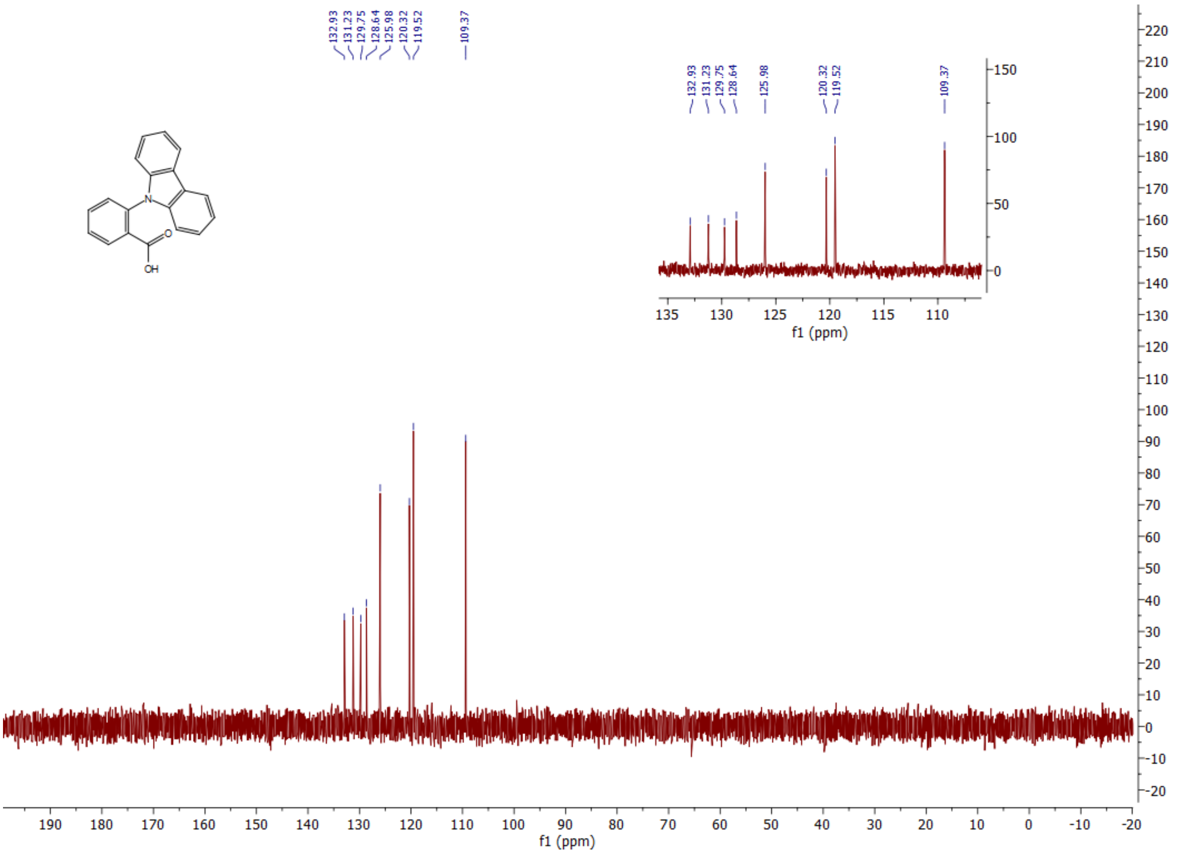

Figure S34. DEPT135 NMR (100 MHz, DMSO-d_6) spectrum of compound **8**.

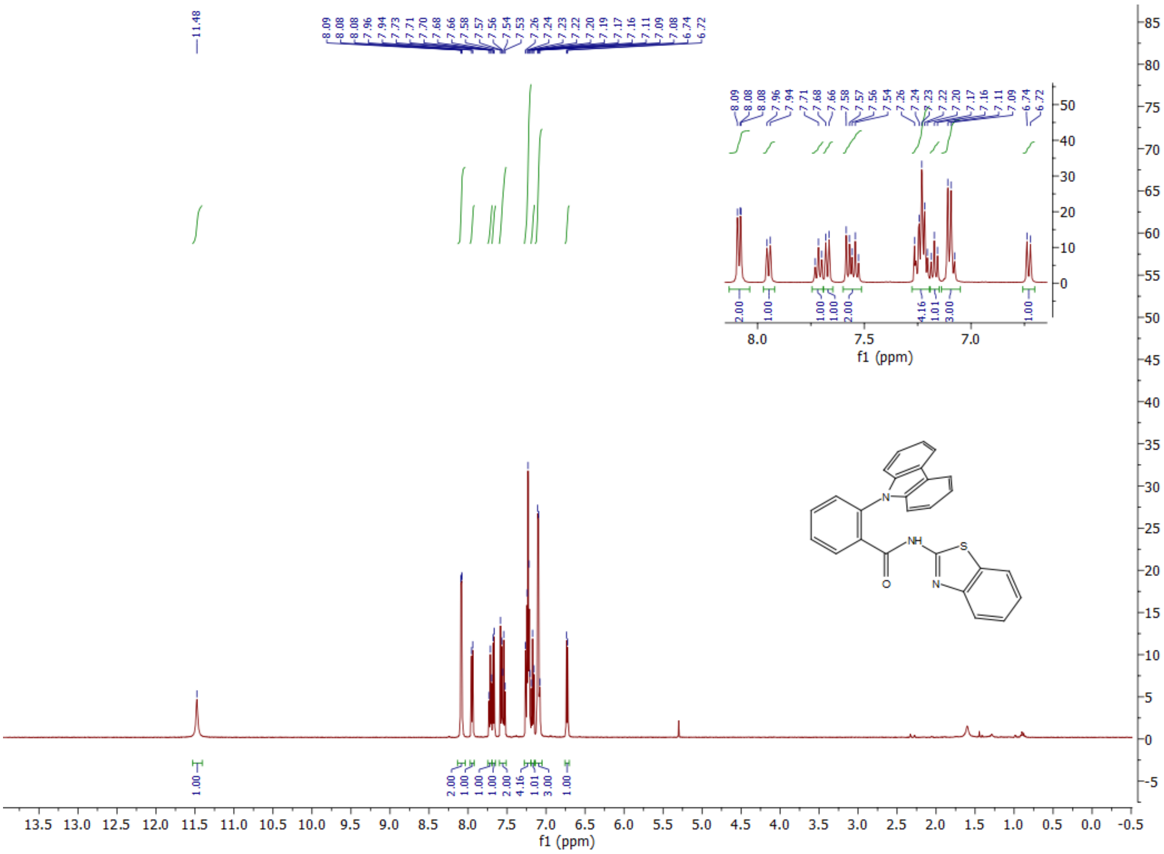

Figure S35. ^{1}H NMR (500 MHz, $CDCl_3$) spectrum of compound **10**.

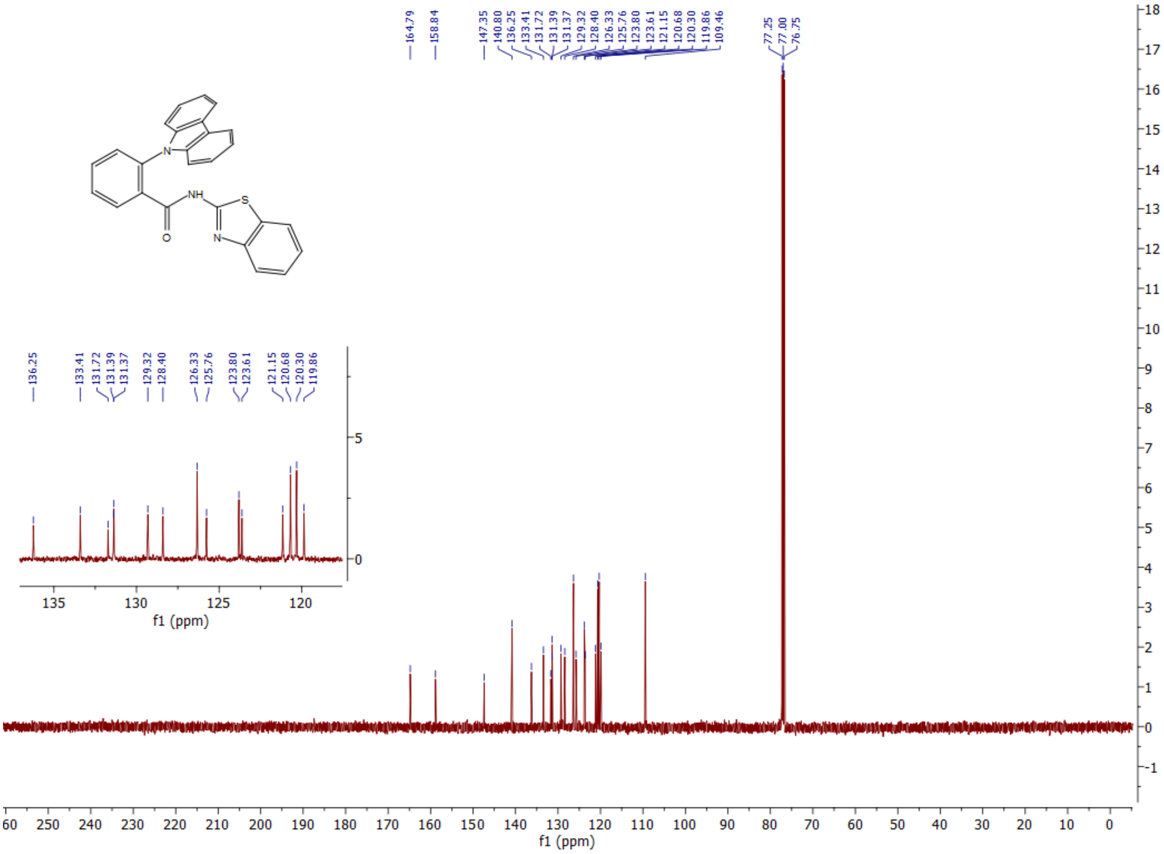

Figure S36. $^{13}C\{H\}$ NMR (125 MHz, $CDCl_3$) spectrum of compound **10**.

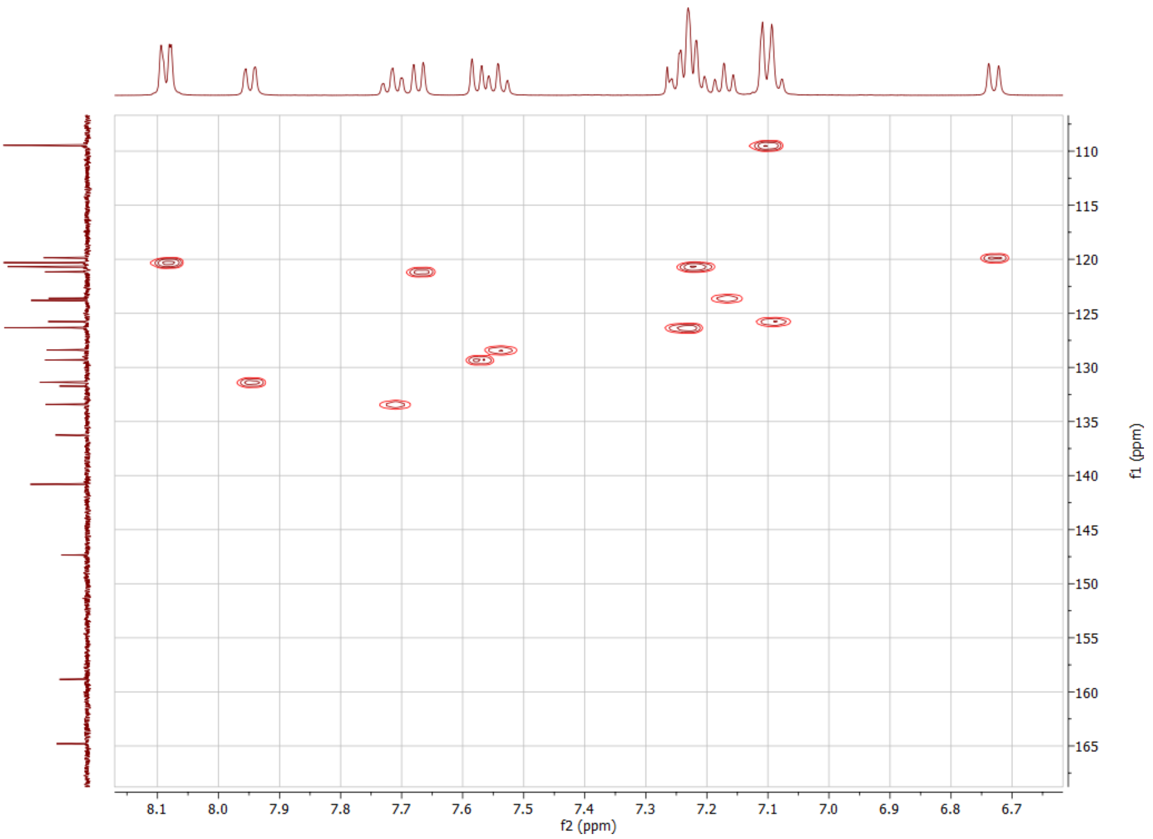

Figure S37. ^{1}H–^{13}C HSQC NMR (125 MHz, $CDCl_3$) spectrum of compound **10**.

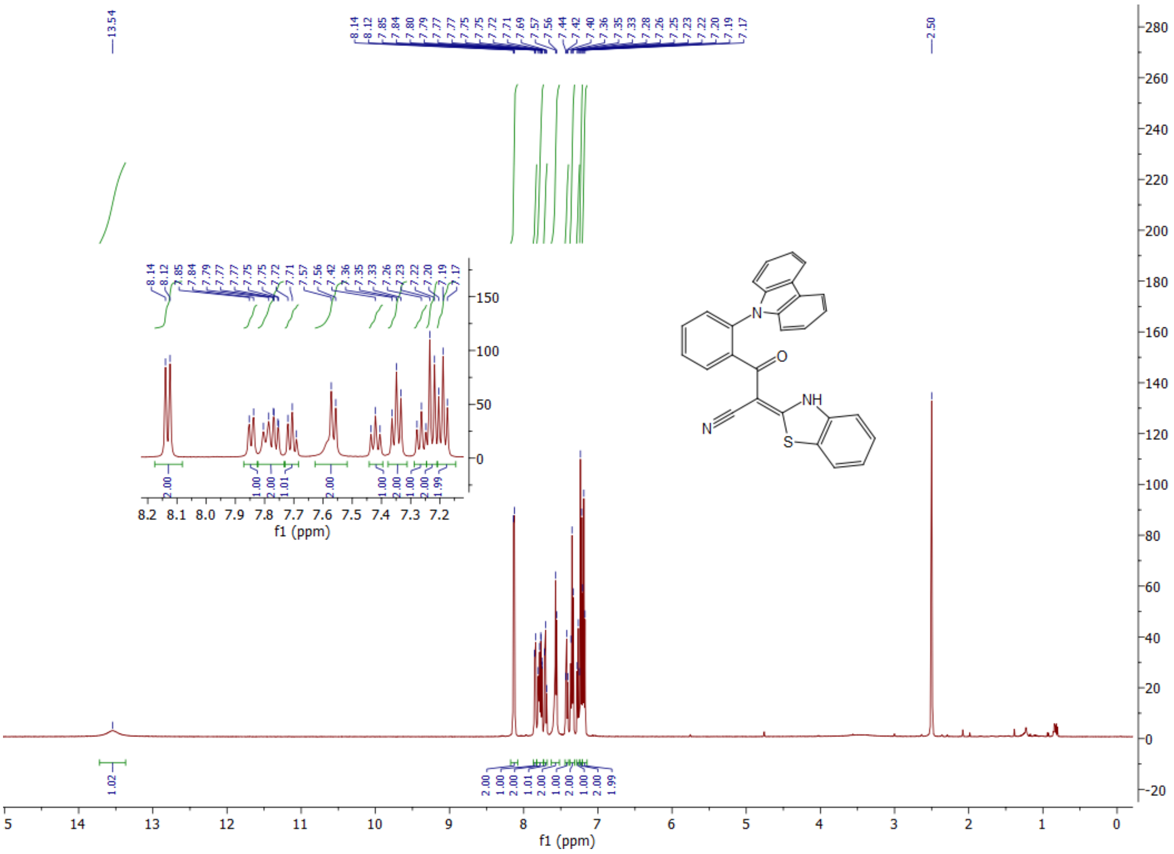

Figure S38. ^{1}H NMR (500 MHz, DMSO-d_6) spectrum of compound **11**.

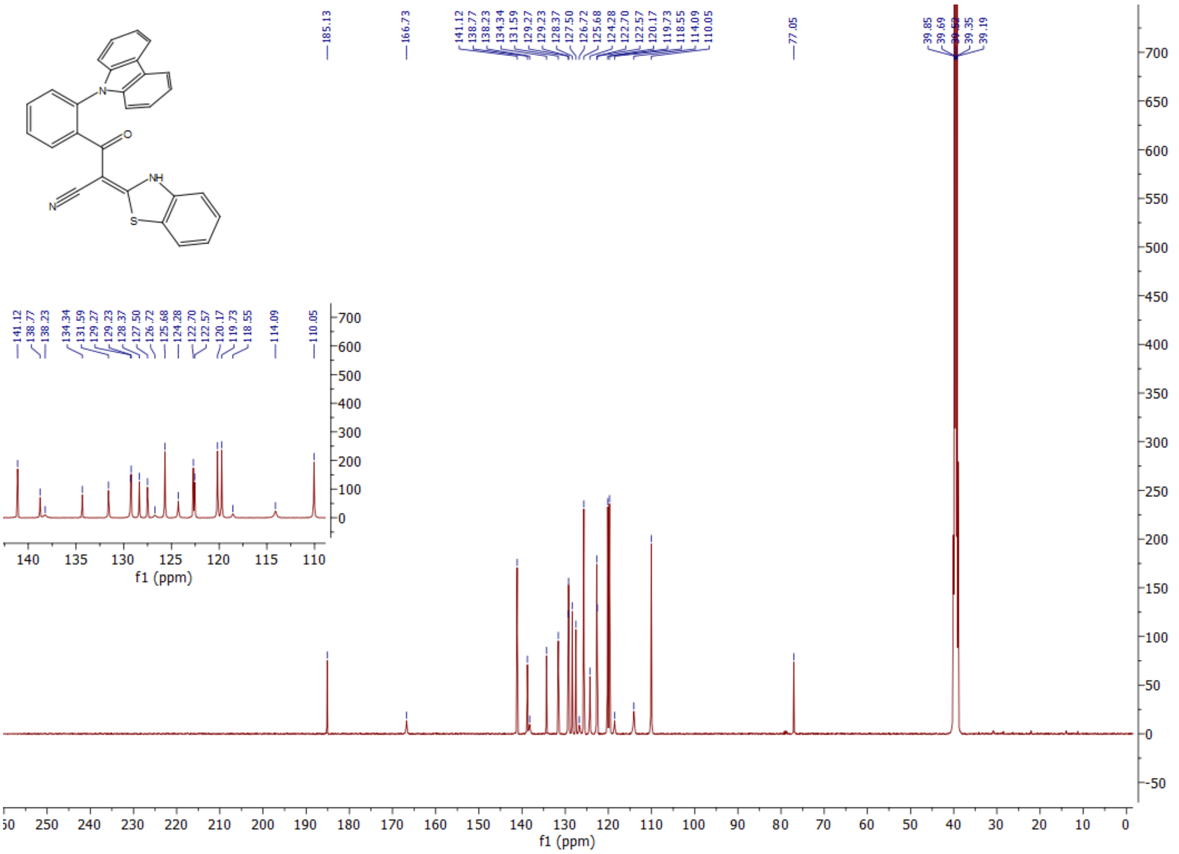

Figure S39. ^{13}C{H} NMR (125 MHz, DMSO-d_6) spectrum of compound **11**.

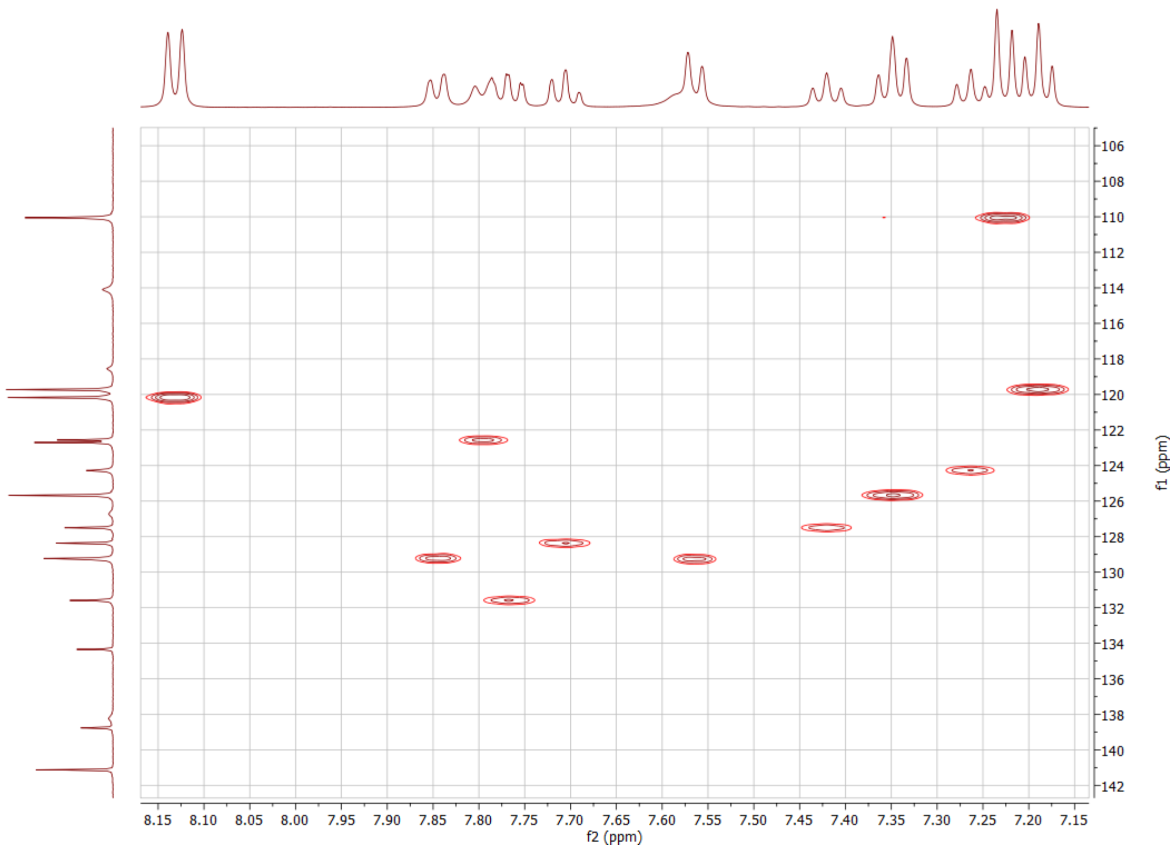

Figure S40. ^{1}H–^{13}C HSQC NMR (125 MHz, DMSO-d_6) spectrum of compound **11**.

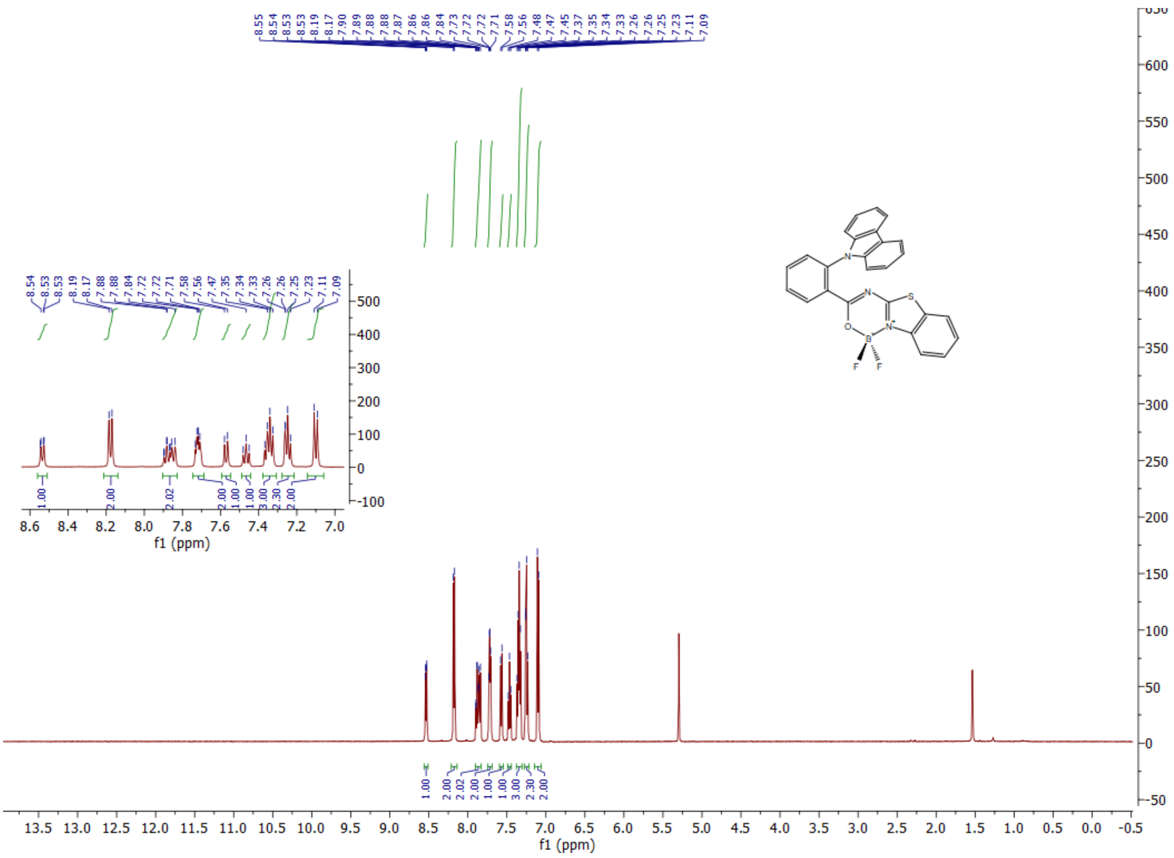

Figure S41. ^{1}H NMR (500 MHz, $CDCl_3$) spectrum of compound **1**.

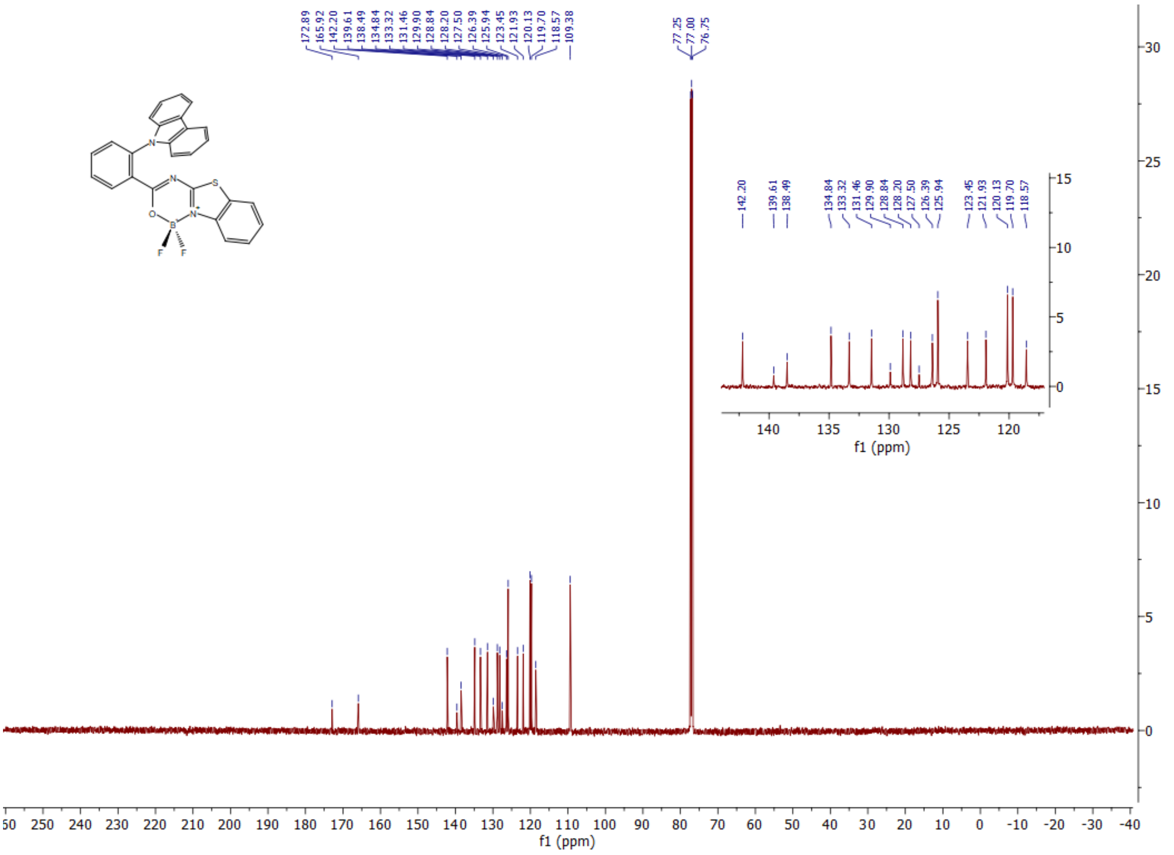

Figure S42. ^{13}C{H} NMR (125 MHz, $CDCl_3$) spectrum of compound **1**.

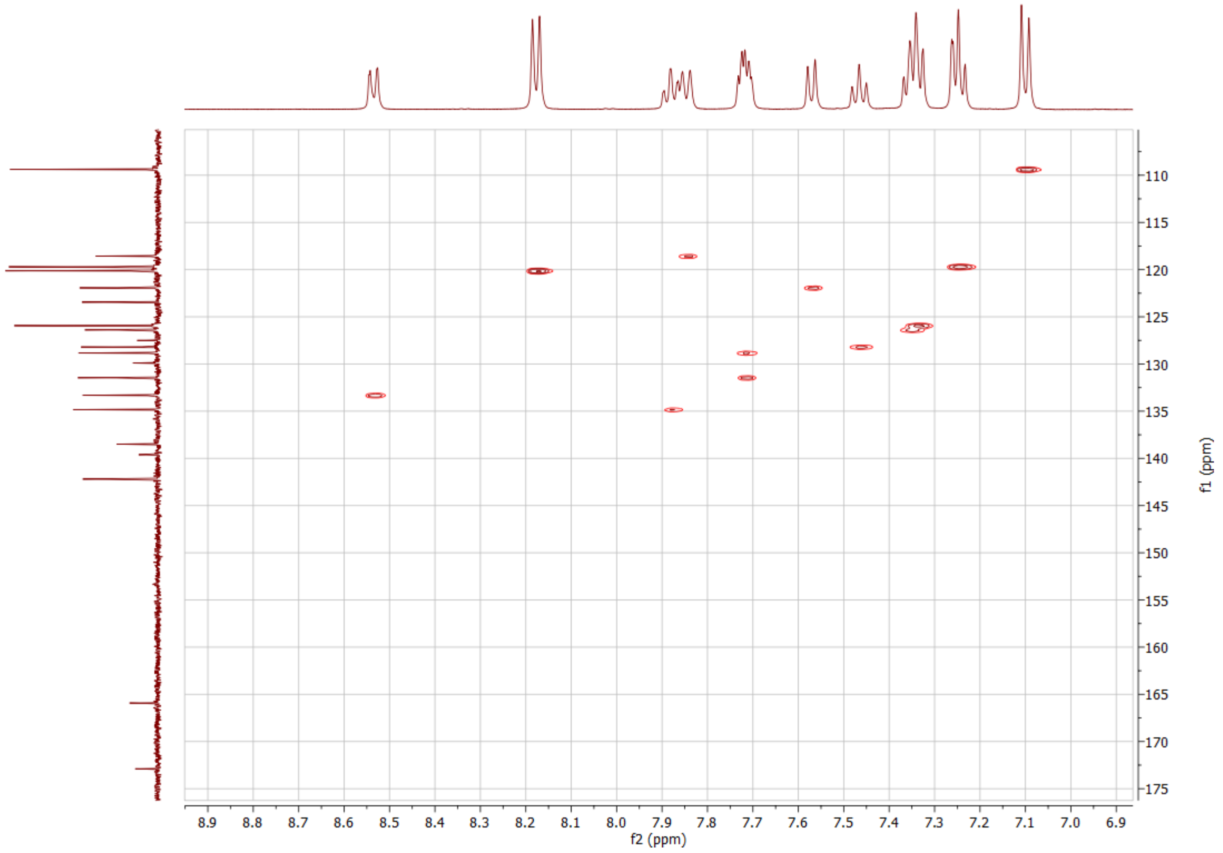

Figure S43. ^{1}H–^{13}C HSQC NMR (125 MHz, $CDCl_3$) spectrum of compound **1**.

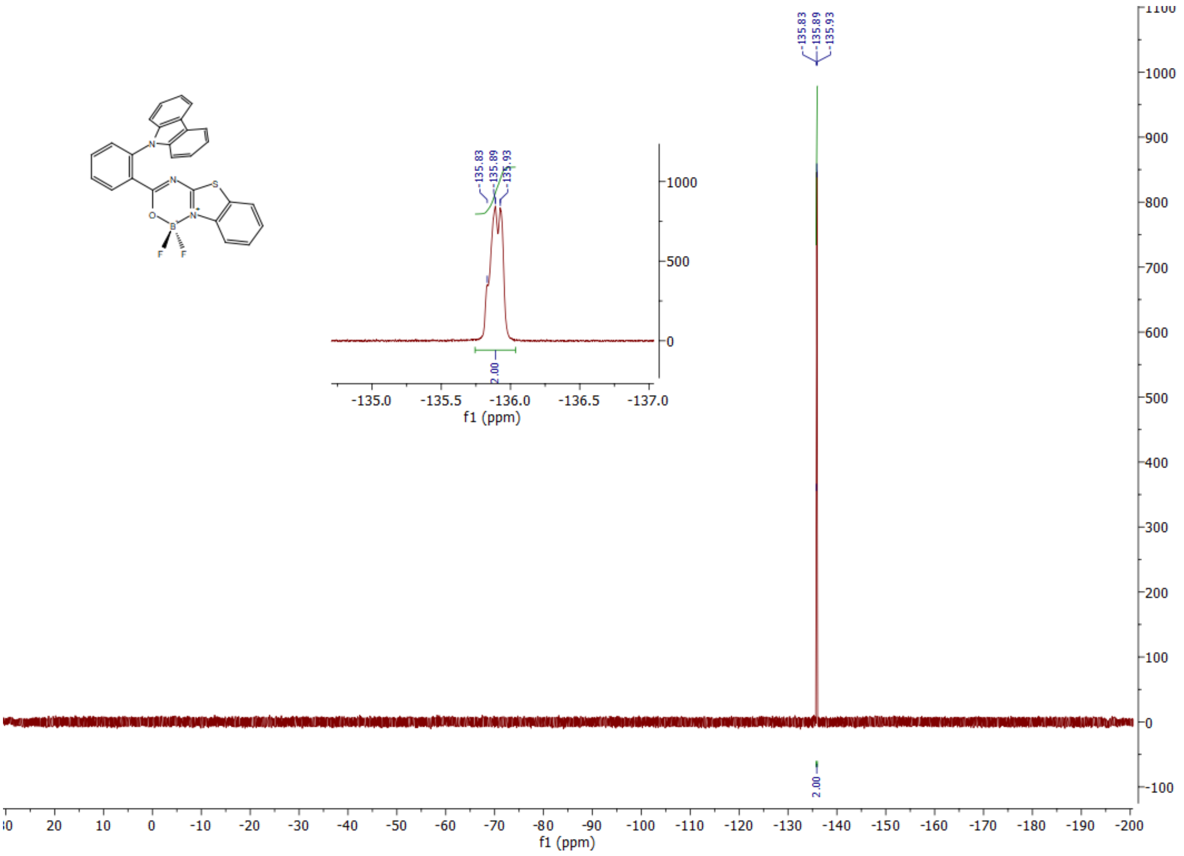

Figure S44. ^{19}F NMR (470 MHz, $CDCl_3$) spectrum of compound **1**.

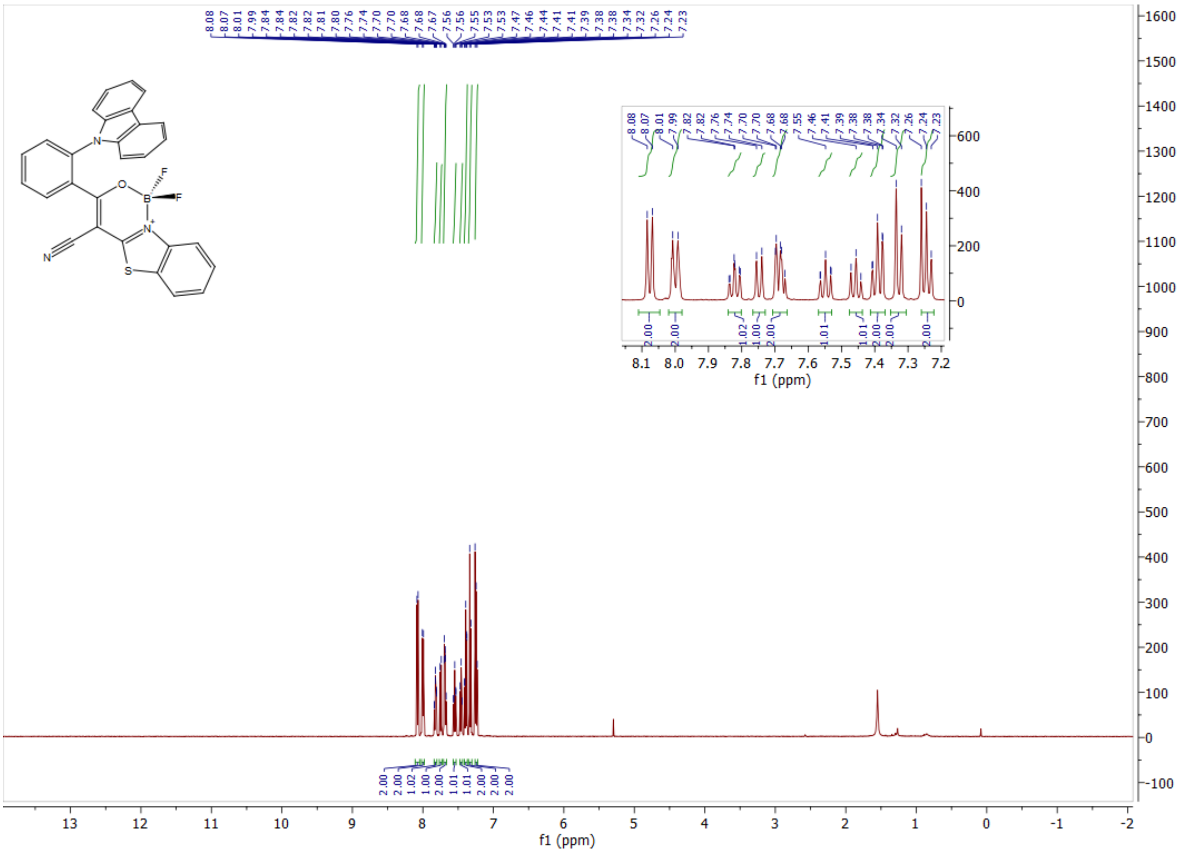

Figure S45. ^{1}H NMR (500 MHz, $CDCl_3$) spectrum of compound **2**.

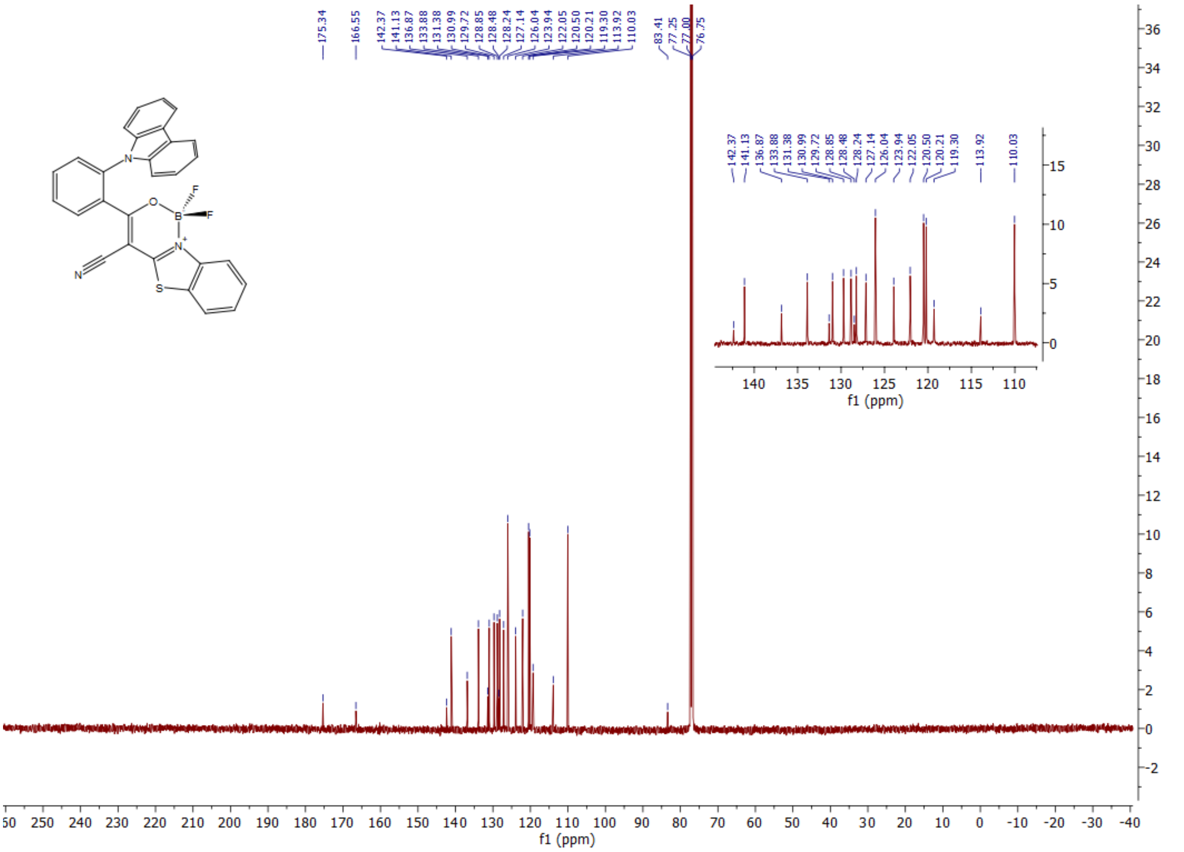

Figure S46. ^{13}C{H} NMR (125 MHz, $CDCl_3$) spectrum of compound **2**.

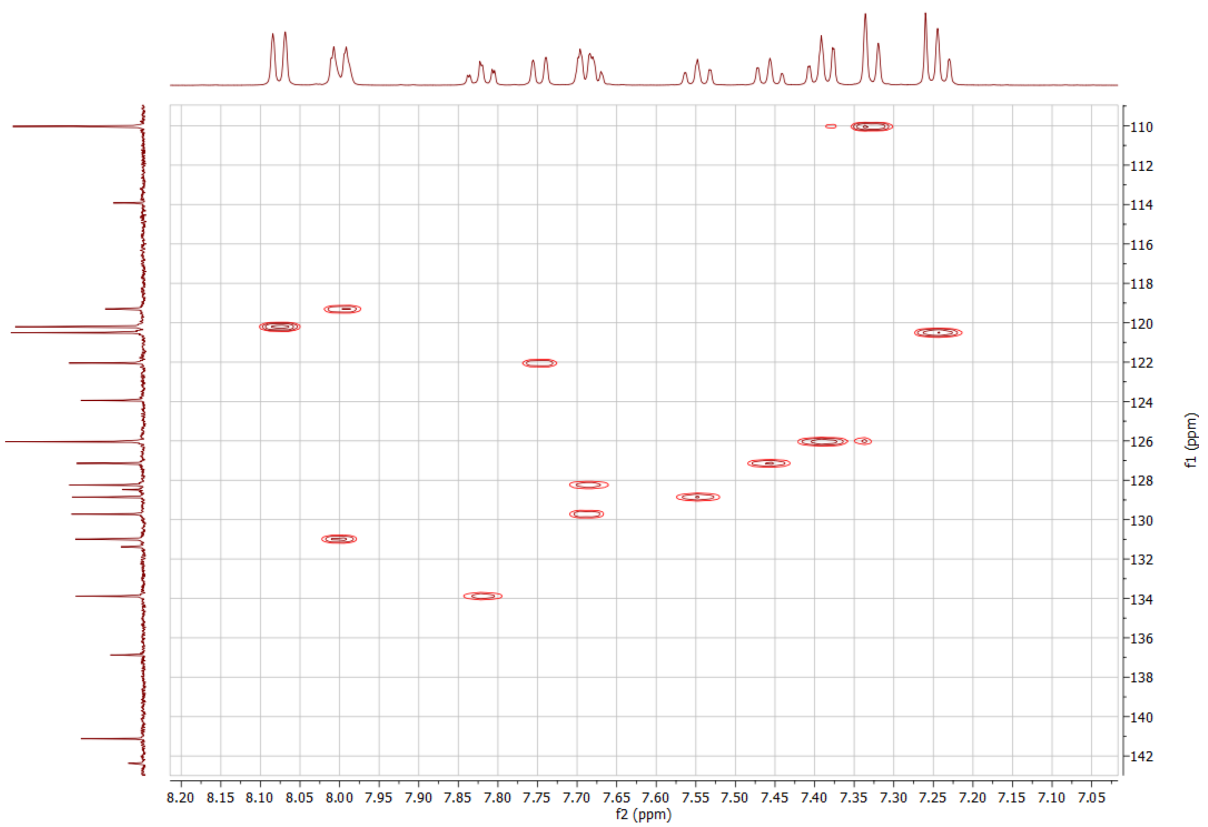

Figure S47. ^{1}H–^{13}C HSQC NMR (125 MHz, $CDCl_3$) spectrum of compound **2**.

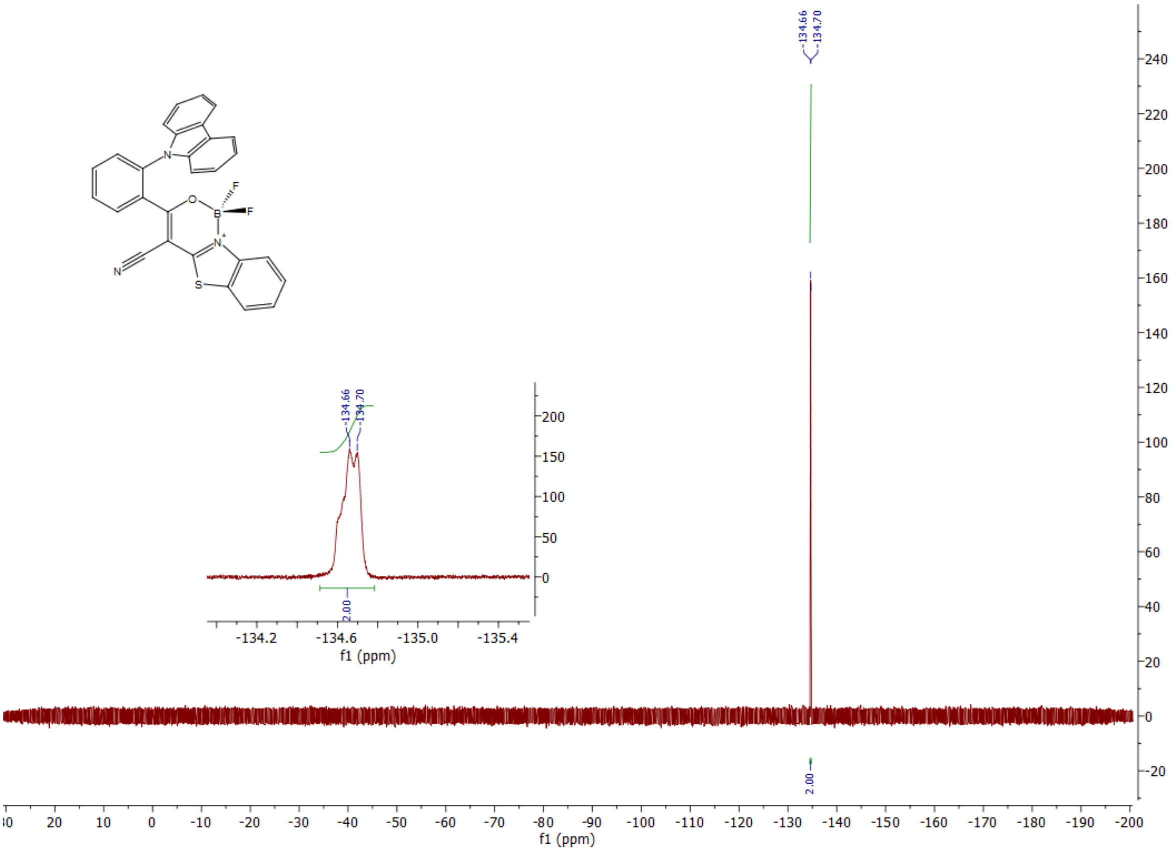

Figure S48. ^{19}F NMR (470 MHz, $CDCl_3$) spectrum of compound **2**.

9. References

[1] Zhang, Q. S.; Kuwabara, H.; Potscavage, W. J.; Huang, S. P.; Hatae, Y.; Shibata, T.; Adachi, C. Anthraquinone-Based Intramolecular Charge-Transfer Compounds: Computational Molecular Design, Thermally Activated Delayed Fluorescence, and Highly Efficient Red Electroluminescence. *J. Am. Chem. Soc.* **2014**, *136*, 18070–18081.

[2] Nikolaenko, A. E.; Cass, M.; Bourcet, F.; Mohamad, D.; Roberts, M. Thermally Activated Delayed Fluorescence in Polymers: A New Route toward Highly Efficient Solution Processable OLEDs. *Adv. Mater.* **2015**, *27*, 7236–7240.
